# Catalytic synthesis of new pyrazolo [3,4-*b*] pyridine via a cooperative vinylogous anomeric-based oxidation

**DOI:** 10.1038/s41598-022-17879-5

**Published:** 2022-08-19

**Authors:** Hassan Sepehrmansourie, Mahmoud Zarei, Mohammad Ali Zolfigol, Saeed Babaee, Saeid Azizian, Sadegh Rostamnia

**Affiliations:** 1 Department of Organic Chemistry, Faculty of Chemistry, Bu-Ali Sina University, Hamedan, 6517838683 Iran; 2 Department of Physical Chemistry, Faculty of Chemistry, Bu-Ali Sina University, Hamedan, 6517838683 Iran; 3 Organic and Nano Group (ONG), Department of Chemistry, Iran University of Science and Technology (IUST), PO Box 16846-13114, Tehran, Iran

**Keywords:** Catalysis, Catalyst synthesis, Heterogeneous catalysis

## Abstract

In this study, a novel nano-magnetic metal–organic frameworks based on Fe_3_O_4_ namely Fe_3_O_4_@MIL-101(Cr)-N(CH_2_PO_3_)_2_ was synthesized and fully characterized. The prepared sample was used as catalyst in the synthesis of pyrazolo [3,4-*b*] pyridines as convenient medicine by condensation reaction of aldehydes, 5-(1*H*-Indol-3-yl)- 2*H*-pyrazol-3-ylamine and 3-(cyanoacetyl)indole via a CVABO. The products were obtained with high yields at 100 °C and under solvent-free conditions.

## Introduction

Nowadays, rational design, synthesis and catalytic systems is an important topic in the research and development (R&D) of the chemical industries^1–6^. Nanoarchitectonics are methods for introducing nanoporous materials such as magnetic-based ones with high surface area^7–9^. Metal–organic frameworks (MOFs) as nanoporous materials have been widely applied in magnetic resonance imaging (MRI)^10,11^, catalysis^12–24^, biotechnology^25^, gas separation^26^, adsorption^27^, purification^28^, drug delivery^29^, etc. The heterogeneous materials based on metallic nanoparticles can play a crucial role in organic synthesis, and have unique properties such as high surface area, easy separation and support for liquid tags materials^30–33^. Therefore, metal–organic frameworks (MOFs) are one of the basic materials for the catalytic preparation of many important molecules in organic synthesis^34–39^. Recently, the synthesis of Fe_3_O_4_ nanomagnetic-based metal–organic frameworks (MOFs) gained attention^40,41^. Because these nanomagnetic-based MOFs catalytic systems have no limitations such as tedious separation and purification^42–45^.

In recent years, many quests have been proceeded to investigate the biological and pharmaceutical properties of the nucleus with indole moieties. We have reviewed the reported methodologies for the synthesis of bis- and trisindolylmethanes^46^. Shiri has comprehensively reviewed the application of indoles in the multicomponent processes^47^. In this regard, fused *N*-heterocycles such as pyridines containing of indole moieties may be suitable candidates for biological and pharmacological chemistry investigations^48,49^. Because, *N*-heterocycles have been used as a drug candidate for antimicrobial, cancer, malaria, anticonvulsant, antifungal, HIV, anti-tumor, antioxidant, antihypertension and urinary incontinence treatment^50,51^. Furthermore, pyridine structure kernels are present in pharmaceutical materials and natural products^52^. Among the *N*-heterocycle compounds, pyrazolo[3,4-*b*] pyridine scaffold is the valuable scaffold of material drugs and KDR kinase inhibition^53,54^. Recent research in material chemistry confirmed that pyrazolo[3,4-*b*] pyridine compounds are key intermediates in industry, semiconductors and organic light-emitting diodes^55^.

**Figure 1:**
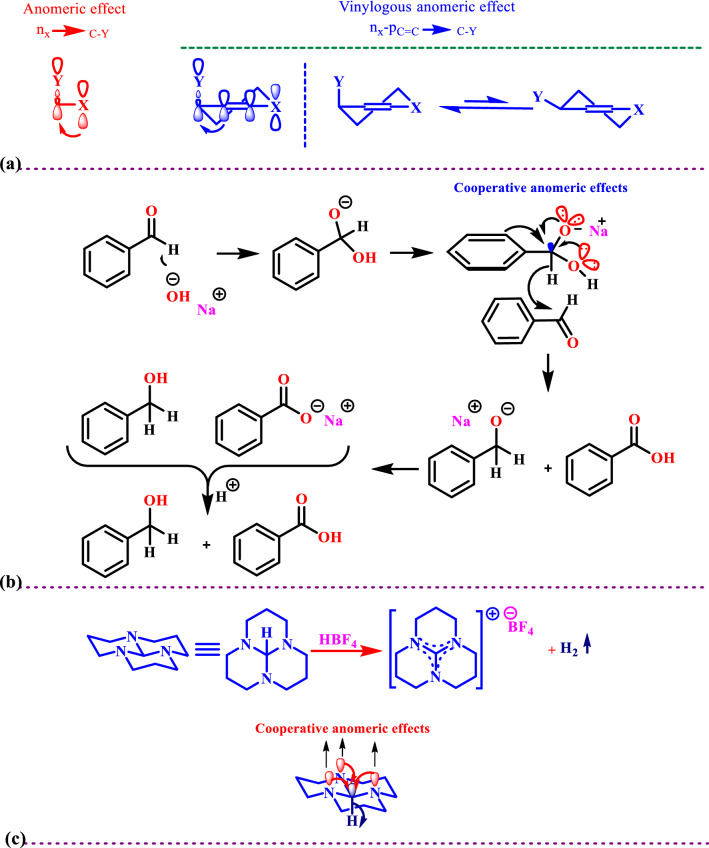
(**a**) Anomeric effect versus the vinylogous anomeric effect. (**b**) A (CABO) leads to hydride transfer in the mechanism of the Cannizzaro reaction. (**c**) Hydrogen releasing supported via a cooperative anomeric effect in the orthoformamide (perhydro-3a,6a,9a-triazaphenalene).

Anomeric effect (AE) as a fundamental example of stereoelectronic interaction has a great educational and research importance. It was discovered in 1955 by J. T. Edward in his studies of carbohydrate chemistry. Historically, this phenomenon was introduced to explain unusual conformational preferences in carbohydrates where the presence of endocyclic oxygen in a glycoside leads to an “abnormal” axial conformational preference for certain substituents at the anomeric carbon^56,57^. In an extension of the classic anomeric effect, a vinyl heteroatom is able to donate electron density from its lone pair through an adjacent π orbital and into the accepting σ* orbital on the other end of the double bond (Fig. 1a).

The anomeric effect can be used as a powerful tool for the justification and interpretation of unusual molecular activities. For example, the forced alignment of three nitrogen non-bonding orbitals with the central antibonding σ*_C–H_ orbital within the ortho formamide and cooperative anomeric effect weakens the bond and leads to an “unusual” hydride transfer under acidic conditions (Fig. 1b)^58–61^.

Recently the term ABO has been introduced^62,63^ and reviewed^64,65^. A famous example of ABO is the Cannizzaro reaction via the addition of hydroxide (OH^−^) to the carbonyl group of aldehydes which do not have *α*-hydrogen (Fig. 1c), both of the lone pair’s electrons of oxygen atoms within the tetrahedral carbon sharing their electrons into the anti-bonding orbital of C–H bond (n_N_→σ^*^_C–H_) and weakened it. The resulting labile hydride acts as a powerful nucleophile that attacks to the second molecule of aldehyde. Finally, this reaction produced equal amounts of the corresponding alcohol and acid.

**Figure 2:**
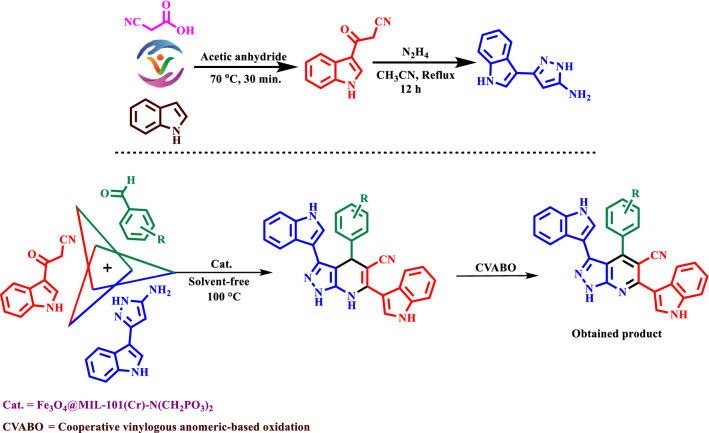
Preparation of novel mono and bis and tris pyrazolo[3,4-*b*] pyridine using Fe_3_O_4_@MIL-101(Cr)-N(CH_2_PO_3_)_2_ as catalyst.

According to the strategy of expanding compounds with biological activities, we have introduced a novel Fe_3_O_4_@MIL-101(Cr)-N(CH_2_PO_3_)_2_ as a catalyst. This magnetic metal–organic framework was used in the synthesis of novel mono, bis and tris pyrazolo [3,4-*b*] pyridines with both indole and pyrazole moieties at 100 °C in solvent-free conditions (Fig. 2).

## Experimental

### Synthesis of Fe_3_O_4_@MIL-101(Cr)-N(CH_2_PO_3_)_2_ as catalyst

**Figure 3:**
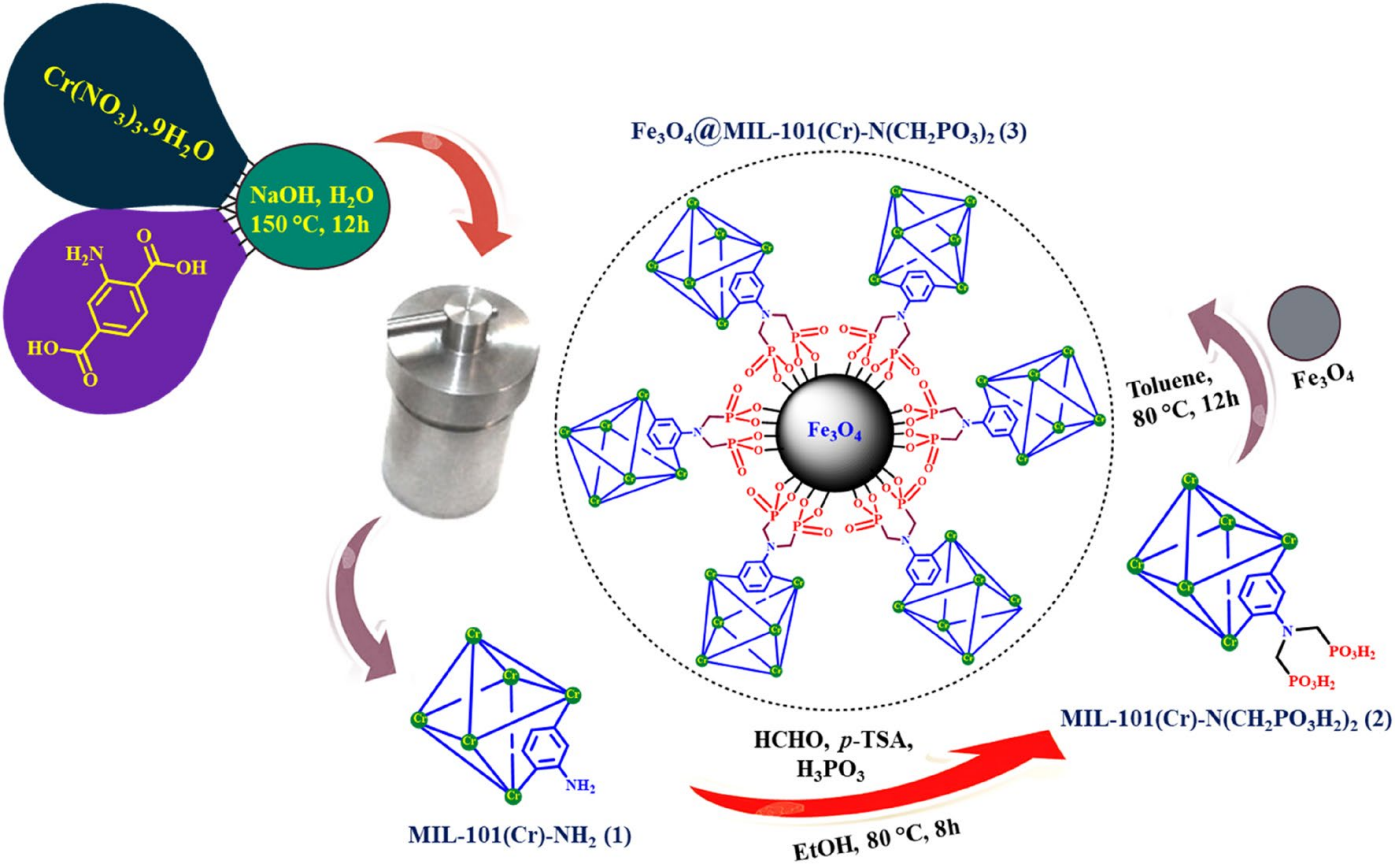
Preparation of nano-magnetic metal–organic frameworks catalyst.

According to the previously reported methods, MIL-101(Cr)-NH_2_ and MIL-101(Cr)-N(CH_2_PO_3_H_2_)_2_ were synthesized^66–68^. Then a mixture of MIL-101(Cr)-N(CH_2_PO_3_H_2_)_2_ (1.3 g) and Fe_3_O_4_ (1 g) were dispersed in toluene at 80 °C for 12 h. After cooling the reaction mixture, the nano-magnetic metal–organic framework Fe_3_O_4_@MIL-101(Cr)-N(CH_2_PO_3_)_2_ was separated by an external magnet and washed with ethanol for several times (Fig. 3).

### General method for the preparation of pyrazolo[3,4-*b*] pyridines

Firstly, the raw materials 3-(cyanoacetyl) indole and 5-(1*H*-Indol-3-yl)-2*H*-pyrazol-3-ylamine were synthesized according to the previously literature reported procedures (Fig. 2)^69,70^. Then, a mixture of aldehyde derivatives (1 mmol), 5-(1*H*-Indol-3-yl)-2*H*-pyrazol-3-ylamine (0.198 g, 1 mmol), 3-(cyanoacetyl) indole (0.184 g, 1 mmol) and nano-magnetic metal–organic frameworks Fe_3_O_4_@MIL-101(Cr)-N(CH_2_PO_3_)_2_ as catalyst (20 mg) were mixed and stirred at 100 °C. Progress of the reaction was followed by applying the TLC technique. After completing the reaction, the reaction system was cooled to 25 °C. Then, hot ethanol was added to the reaction mixture, and the catalyst was removed from the reaction mixture with an external magnet. The ethanol was evaporated, and finally, the pyrazolopyridine product was purified by plate chromatography (EtOAc/*n*-hexane:1/1). ^1^H NMR (400 MHz, DMSO-*d*_*6*_), ^13^C NMR (100 MHz, DMSO-*d*_*6*_), FT-IR (KBr, cm^−1^) and melting point analysis were used for all synthesized molecules.

## Result and discussion

Nowadays, synthesized molecules bearing pyridine-based scaffolds with indole moieties are attracted the attention of pharmaceutical chemistry researchers due to their building block’s ability in pharmaceuticals and modern drug design approaches. So, there still is a great demand for the introducing of more practical and facile procedures and catalytic systems for multicomponent reactions in the organic synthesis field. On the other hand, the joining of indole, pyridine, and pyrazole moieties within a single molecule is our main research proposal in this investigation. We think that combining of above-mentioned units in a single structure can open up a new and promising insight in the course of rational design, synthesis and applications of drug candidate compounds. According to our recent findings^71–78^, we believed that the stereoelectronic effect has a major role in the last step of the proposed mechanism. To the best of our knowledge and literature surveys, there is no report on the synthesis of described pyridines. Therefore, herein we wish to report the first catalytic and multicomponent method for the preparation of new pyridines with both pyrazole and indole aromatic moieties via CVABO.

In order to extend the field of magnetic metal–organic frameworks catalysts, our research group has presented and synthesized a novel MIL-101(Cr)-N(CH_2_PO_3_H_2_)_2_ connected to Fe_3_O_4_ tags (Fig. 2). The structure of Fe_3_O_4_@MIL-101(Cr)-N(CH_2_PO_3_)_2_ as a magnetic metal–organic frameworks catalyst was characterized by FT-IR, XRD, SEM, EDX, SEM-elemental mapping, TG, DTG and *N*_*2*_ adsorption–desorption isotherm (BET analysis). Then, the magnetic metal–organic frameworks catalyst was applied for the preparation of novel pyrazolo[3,4-*b*] pyridine derivatives as biological candidates.

**Figure 4:**
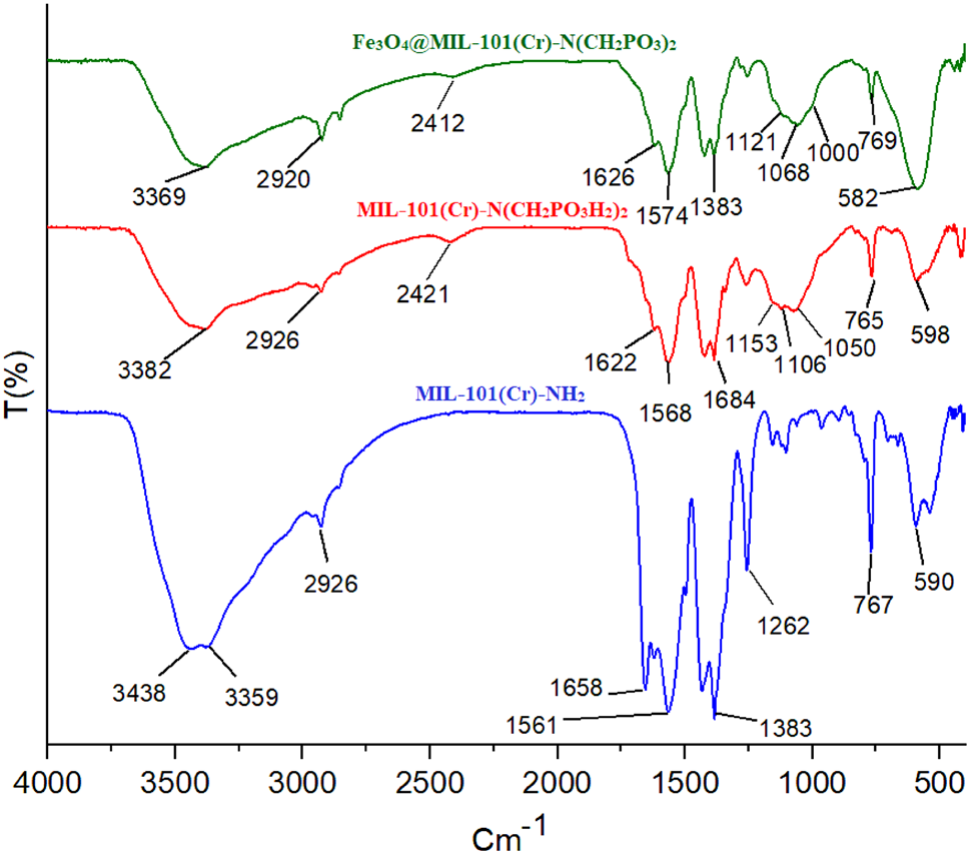
FT-IR spectra of catalyst and starting materials.

The FT-IR spectra of Fe_3_O_4_@MIL-101(Cr)-N(CH_2_PO_3_)_2_ as the catalyst, MIL-101(Cr)-N(CH_2_PO_3_H_2_)_2_ and MIL-101(Cr)-NH_2_ were shown in Fig. 4. The broadband at 2600–3500 cm^−1^ is related to OH of PO_3_H_2_ functional groups in MIL-101(Cr)-N(CH_2_PO_3_H_2_)_2_. The peaks P–O and P=O bond stretching are shown in 1000 cm^−1^, 1068 cm^−1^ and 1121 cm^−1^ respectively. Also, the absorption peaks at 2920 and 1626 cm^−1^ are related to aromatic C–H and C=C stretching bands. Furthermore, the peak at 582 cm^−1^ is related to the stretching vibrational modes of Fe–O groups in Fe_3_O_4_. The FT-IR difference between starting materials and Fe_3_O_4_@MIL-101(Cr)-N(CH_2_PO_3_)_2_ as catalyst verified the scaffold of the catalyst.

**Figure 5:**
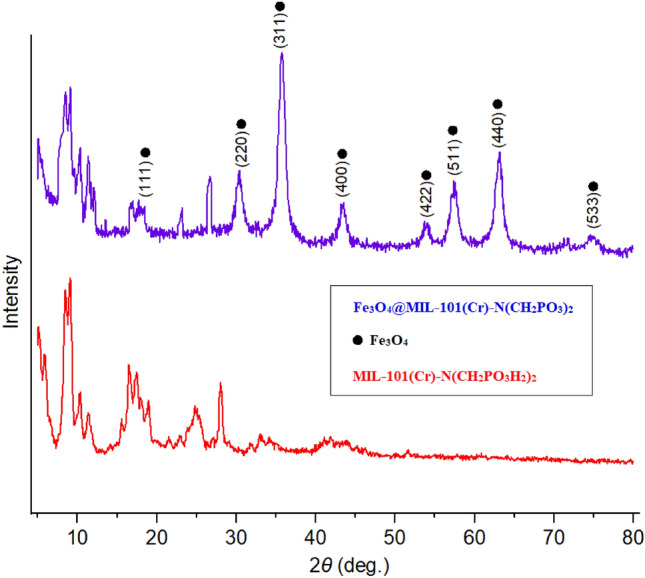
Comparison XRD pattern of catalyst and MIL-101(Cr)-N(CH_2_PO_3_H_2_)_2_ as starting material.

The particle size and phase of Fe_3_O_4_@MIL-101(Cr)-N(CH_2_PO_3_)_2_ as catalyst and MIL-101(Cr)-N(CH_2_PO_3_H_2_)_2_ were investigated by XRD at the range of 5–80°, Fig. 5. The XRD patterns demonstrated diffraction lines of high crystalline nature at 2θ = 18.0°, 30.3°, 35.5°, 43.6°, 54.0°, 57.3°, 62.7° and 74.6° correspond to the Fe_3_O_4_ diffraction lines (111), (220), (311), (400), (422), (511), (440) and (533) (Fig. 5)^79^. Also, the Scherer equation and Bragg equation were used for the averaged inter planer distance and sizes of crystal, which are determined 5.1–26.4 nm (Table 1).

**Table 1:** XRD data of catalyst.

Entry	2θ	Peak width (degree)	Size [nm]	Inter planer distance [nm]
1	8.20	0.30	26.4	1.074
2	23.15	0.55	14.6	0.383
3	26.80	0.55	14.7	0.331
4	30.55	1.6	5.1	0.291
5	35.70	1.15	7.2	0.251
6	43.40	0.67	12.6	0.208
7	57.55	1	9.0	0.160
8	63.05	1	9.2	0.147

**Figure 6:**
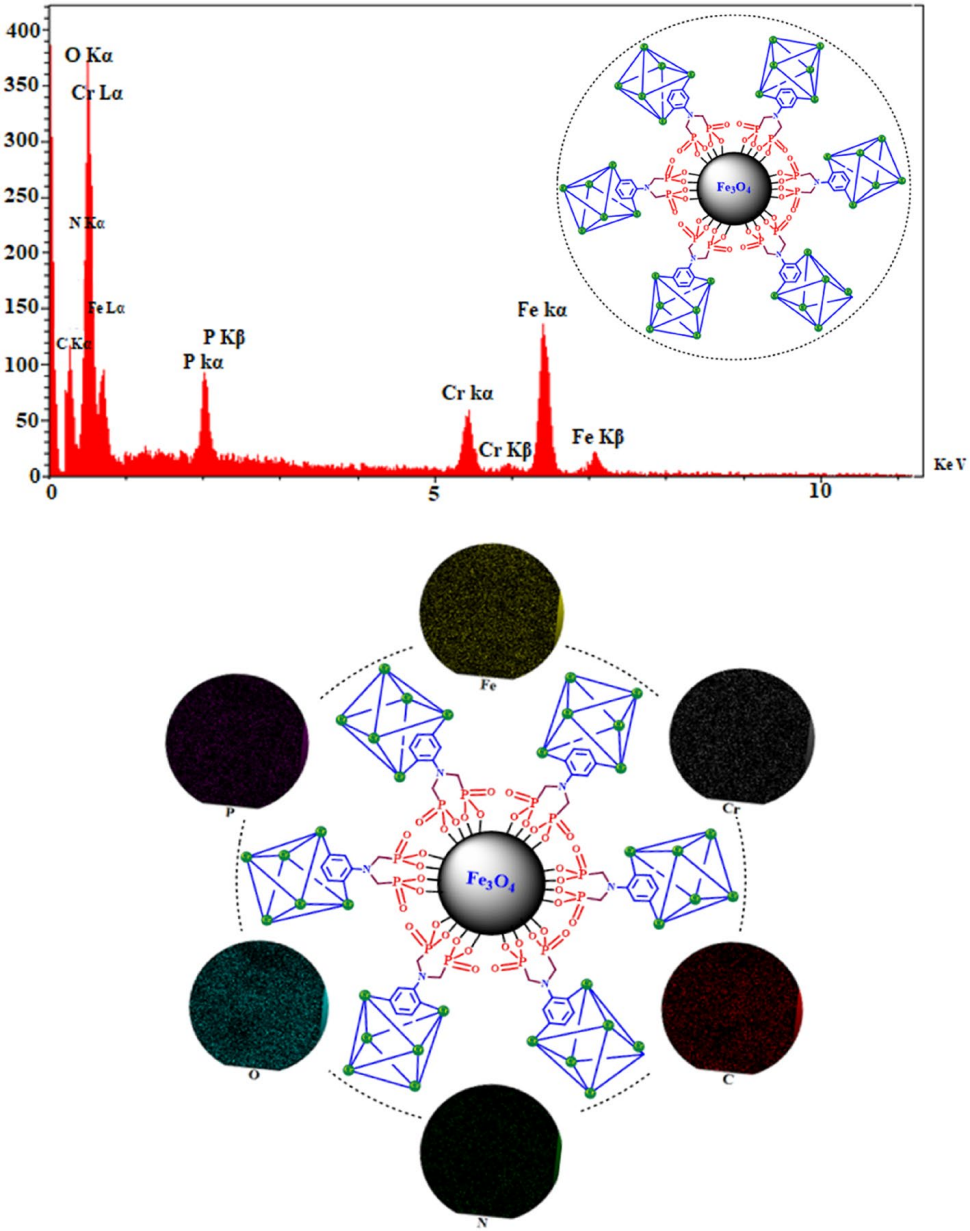
Fe_3_O_4_@MIL-101(Cr)-N(CH_2_PO_3_)_2_: **(a)** EDX analysis. **(b)** Elemental mapping of C (red), O (blue), N (green), Fe (yellow), P (purple) and Cr (gray) atoms.

For further verification of the scaffold and elemental analysis in the prepared catalyst, the energy dispersive X-ray analysis (EDX) analysis was used (Fig. 6a). The constituents of the catalyst were verified with the existence of Fe, N, Cr, O, C and P atoms. Figure 6b shows that the elementals of Fe_3_O_4_@MIL-101(Cr)-N(CH_2_PO_3_)_2_ are distributed uniformly.

**Figure 7:**
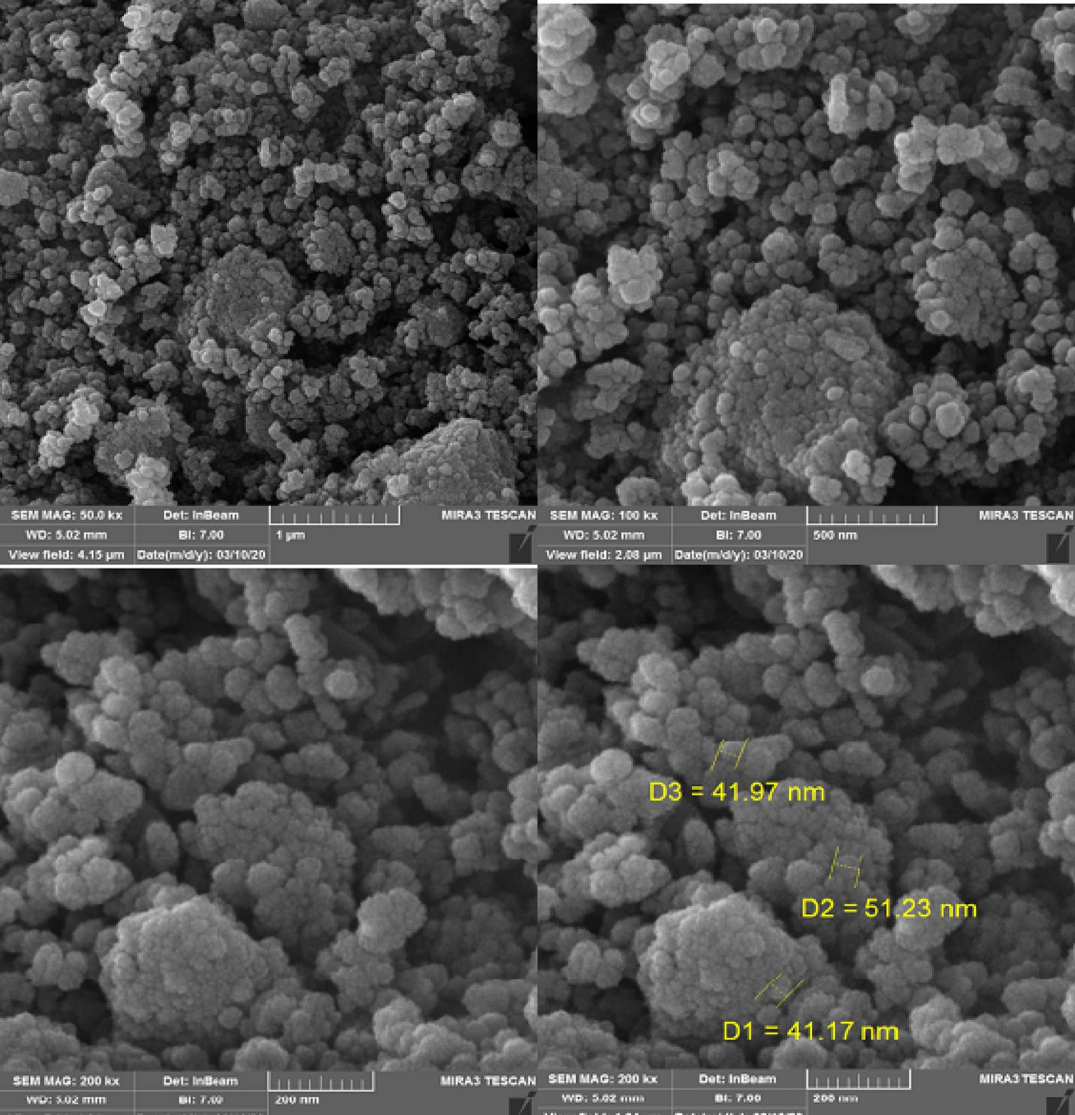
Scanning electron microscope (SEM) images of catalyst.

In another study, the particle size and morphology of Fe_3_O_4_@MIL-101(Cr)-N(CH_2_PO_3_)_2_ as catalyst were studied by scanning electron microscope (SEM) (Fig. 7). As shown in Fig. 7 the particles have spherical shape in the nanoscale size.

**Figure 8:**
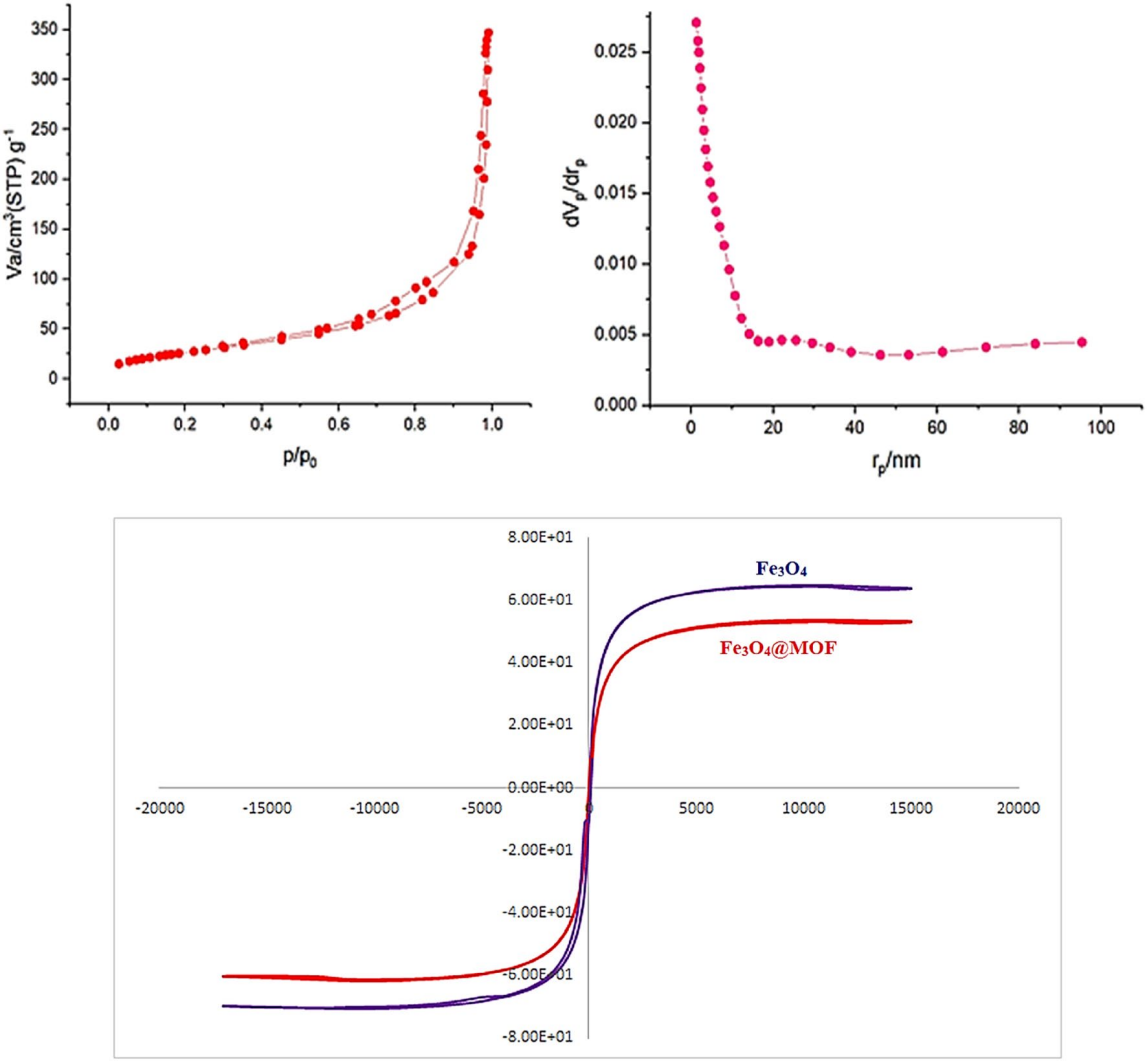
Fe_3_O_4_@MIL-101(Cr)-N(CH_2_PO_3_)_2_: (**a**) BET and BJH isotherms, (**b**) VSM.

The *N*_*2*_ adsorption/desorption isotherms were utilized to analyze the textural features of Fe_3_O_4_@MIL-101(Cr)-N(CH_2_PO_3_)_2_ (Fig. 8a). A hysteresis loop is observed, indicating the presence of mesopores in the structure of the sample. The calculated surface area (BET) and the total pore volume are 100.03 m^2^ g^−1^ and 0.518 cm^3^ g^−1^, respectively. The plot of the pore size distribution of Fe_3_O_4_@MIL-101(Cr)-N(CH_2_PO_3_)_2_ obtained by the BJH method is presented in (Fig. 8a). This plot reveals that the catalyst possesses both micropores (size < 2 nm) and mesopores (2 < size < 50 nm), however, the radius of most of the pores is less than 10 nm. Figure 8b depicts the magnetic measurements of Fe_3_O_4_@MIL-101(Cr)-N(CH_2_PO_3_)_2_ and Fe_3_O_4_. The vibrating sample magnetometer (VSM) of Fe_3_O_4_ and Fe_3_O_4_@MIL-101(Cr)-N(CH_2_PO_3_)_2_ were compared, the vibrating sample magnetometer of pure Fe_3_O_4_ reduced from 64.5 up to 53.5 mug^−1^ for the Fe_3_O_4_@MIL-101(Cr)-N(CH_2_PO_3_)_2_. Therefore, this reduction is for the coating of Fe_3_O_4_@MIL-101(Cr)-N(CH_2_PO_3_)_2_ onto the surface of Fe_3_O_4_.

**Figure 9:**
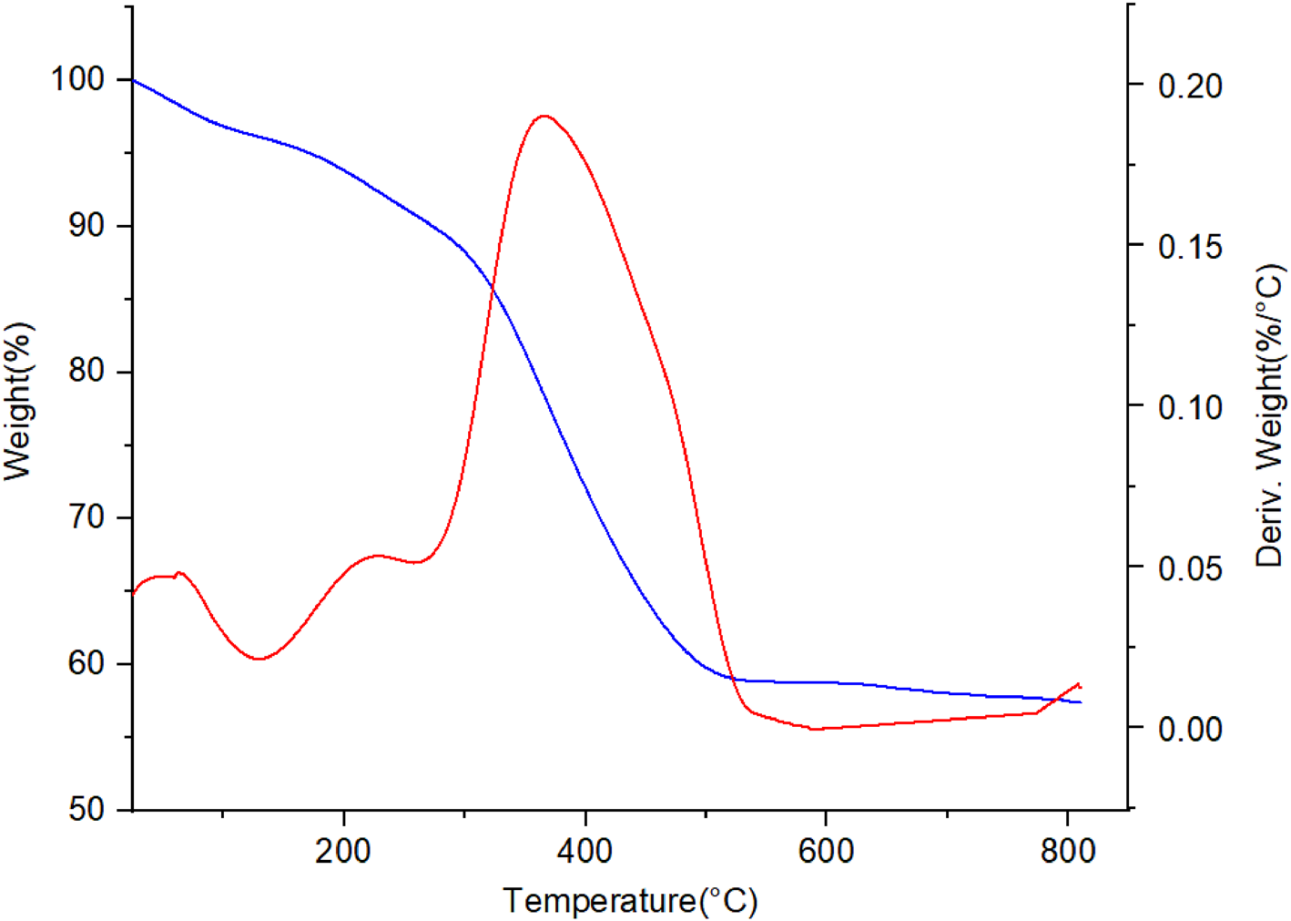
Thermal gravimetric (TG) and derivative thermal gravimetric (DTG) analysis of Fe_3_O_4_@MIL-101(Cr)-N(CH_2_PO_3_)_2_.

The thermal and behavioral stability of nano-magnetic metal–organic frameworks Fe_3_O_4_@MIL-101(Cr)-N(CH_2_PO_3_)_2_ were studied by thermal gravimetric (TG) and derivative thermal gravimetric (DTG) techniques (Fig. 9). The first step is the weight loss, which took place between 25 and 100 °C, associated with the removal of solvents (organic and water). The main stage of weight loss, disrupts the structure of Fe_3_O_4_@MIL-101(Cr)-N(CH_2_PO_3_)_2_, took place at 380 °C, and includes about 35% weight loss. Therefore, this catalyst can be used up to 300 °C.

After the synthesis and characterization of Fe_3_O_4_@MIL-101(Cr)-N(CH_2_PO_3_)_2_, it was used for the synthesis of new pyrazolo [3,4-*b*] pyridine derivatives with indole and pyrazole tags. The mentioned compounds were prepared by reaction of 4-chloro benzaldehyde (0.141 g, 1.0 mmol), 5-(1*H*-Indol-3-yl)-2*H*-pyrazol-3-ylamine (0.198 g, 1 mmol) and 3-(cyanoacetyl) indole (0.184 g, 1 mmol) as a suitable model for the optimization the reaction conditions. The results are assembled in Table 2, the best option for the preparation of compound C2 was achieved in the presence of 20 mg Fe_3_O_4_@MIL-101(Cr)-N(CH_2_PO_3_)_2_ in solvent-free conditions (Table 2, entry 2). The target reaction was also examined by using several solvents such as DMF, CH_3_CN, MeOH, H_2_O, EtOH, CH_2_Cl_2_, CHCl_3_, and EtOAc (5 mL) in the presence of 20 mg of catalyst. The reaction results did not improve (Table 2, entries 9–17). These interesting results encouraged us to synthesis a wide range of pyrazolo [3,4-*b*] pyridine compounds in solvent-free conditions.

**Table 2:** Optimization parameter on the synthesis **C2** compound.


Entry	Solvent	Catalyst (mg)	Temp. (°C)	Time	Yield (%)
1	–	20	110	60 (min)	80
2	–	20	100	60 (min)	87
3	–	20	90	60 (min)	82
4	–	20	50	60 (min)	10
5	–	20	25	60 (min)	–
6	–	30	100	60 (min)	85
7	–	100	100	60 (min)	56
8	–	5	100	60 (min)	25
9	DMF	20	Reflux	7 (h)	83
10	DMF	20	100	8 (h)	82
11	CH_3_CN	20	Reflux	10 (h)	38
12	MeOH	20	Reflux	8 (h)	15
13	H_2_O	20	Reflux	9 (h)	10
14	EtOH	20	Reflux	10 (h)	45
15	CH_2_Cl_2_	20	Reflux	12 (h)	–
16	CHCl_3_	20	Reflux	12 (h)	–
17	EtOAc	20	Reflux	10 (h)	–

As above-mentioned, after optimizing the reaction conditions, catalyst (20 mg) is used for the preparation of new biomolecules products using widespread aldehydes such as bearing electron-donating, electron-withdrawing groups, iso-trephetaldehyde and tris-aldehyde, 5-(1*H*-Indol-3-yl)-2*H*-pyrazol-3-ylamine and 3-(cyanoacetyl)indole. The results can be seen in Table 3, the obtained results show that the prepared catalyst is suitable for the synthesis of mono, bis and tris products with high yields (70–90%) and short reaction time (35–60 min).

**Table 3:**
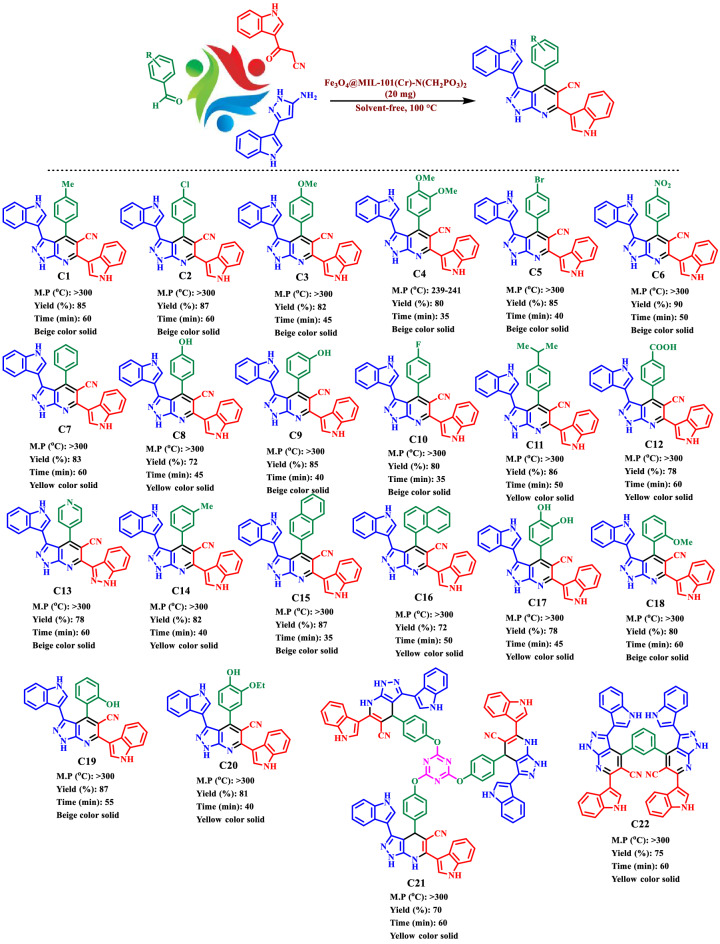
Synthesis of pyrazolo[3,4-*b*] pyridine derivatives (**C1**–**C22**) using Fe_3_O_4_@MIL-101(Cr)-N(CH_2_PO_3_)_2_ as a catalyst.

**Figure 10:**
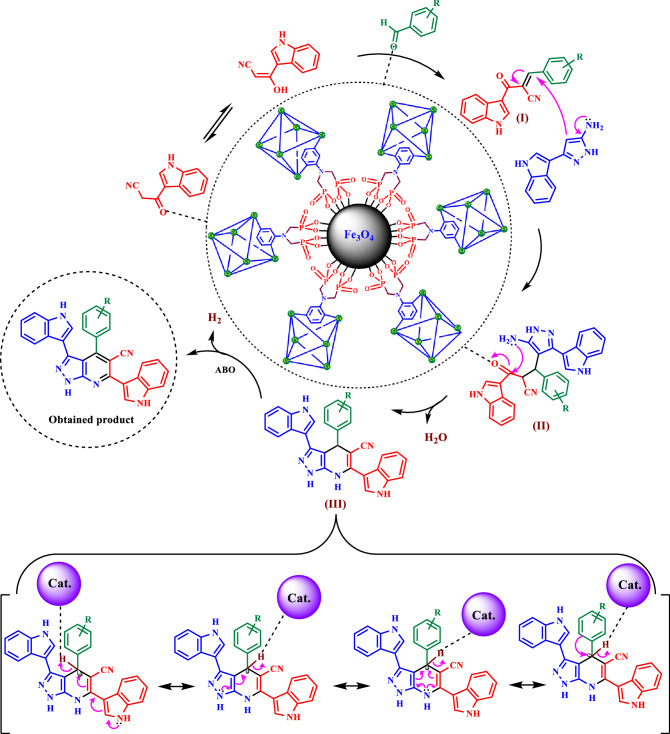
Proposed mechanism for the synthesis of pyrazolo[3,4-*b*]pyridine derivatives using Fe_3_O_4_@MIL-101(Cr)-N(CH_2_PO_3_)_2_ as catalyst.

**Figure 11:**
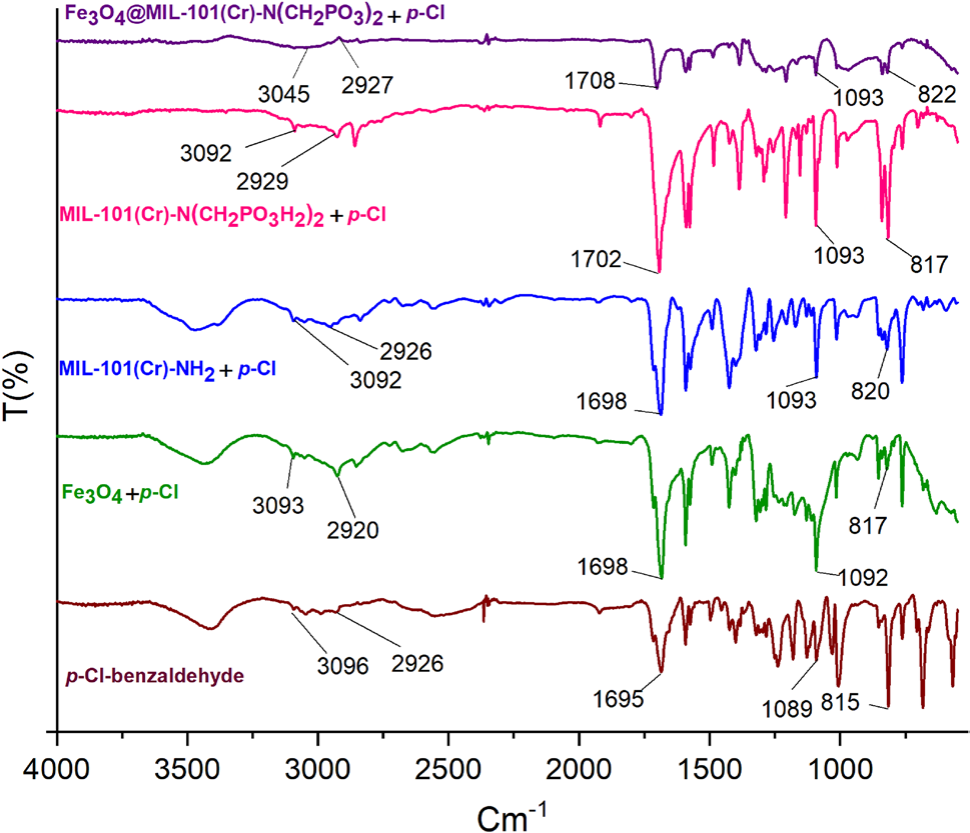
FT-IR spectra of *p*-Cl-benzaldehyde in percent of Fe_3_O_4_, MIL-101(Cr)-NH_2_, MIL-101(Cr)-N(CH_2_PO_3_H_2_)_2_ and Fe_3_O_4_@MIL-101(Cr)-N(CH_2_PO_3_).

In the suggested mechanism, the catalyst activates the carbonyl group of aldehyde. Firstly, intermediate (**I**) as a Michael acceptor is produced by the reaction of activated aldehyde and 3-(cyanoacetyl)indole. In the next step, intermediate (**II**) is prepared from reactions of 5-(1*H*-Indol-3-yl)-2*H*-pyrazol-3-ylamine and intermediate (**I**). In the third step, intermediate (**III**) is obtained by cyclization and losing one molecule of H_2_O. Then, intermediate (**III**) via interaction of lone pair electrons of N atoms from C=C bonds causes the release hydride and H_2_. Finally, the 1,4-dihydropyridines convert to their corresponding pyrazolo [3,4-*b*] pyridine derivatives via a CVABO and release a hydrogen molecule (–H_2_)^80,81^. The optimization of model reaction under argon and N_2_ atmospheres are also verified the desired product (Fig. 10). To investigate the activation of aldehyde by catalyst, *p*-chloro benzaldehyde was activated with Fe_3_O_4_@MIL-101(Cr)-N(CH_2_PO_3_)_2_ at room temperature. Then the FT-IR spectra of the reaction mixtures were examined. The absorption band of C=O of the *p*-chloro benzaldehyde at 1708 cm^−1^ was changed to 1702, 1698, 1698 cm^−1^ in the presence of MIL-101(Cr)-N(CH_2_PO_3_)_2_, MIL-101(Cr)-NH_2_ and Fe_3_O_4_ (Fig. 11).

To compare the efficiency of the synthesized catalyst in the preparation of pyrazolo [3,4-*b*] pyridine derivatives, the model reaction (compound C2) was tested using various inorganic and organic catalysts under optimal conditions (Table 4). As Table 4 shows, nano-magnetic metal–organic frameworks Fe_3_O_4_@MIL-101(Cr)-N(CH_2_PO_3_)_2_ is the best catalyst for the preparation of the desired product. Also, to investigate the heterogeneous nature of the protocols and palladium leaching, ICP/MS results proved that no Fe and Cr leaching was detected in the filtrate (Fe: 2.5 × 10^−6^ and Cr: 1.9 × 10^−5^ mol g^−1^ respectively) upon reaction completion, which indicates the high stability of the prepared catalyst.

**Table 4:** Compare of various catalyst for the synthesis of pyrazolo [3,4-*b*] pyridine derivatives in comparison with Fe_3_O_4_@MIL-101(Cr)-N(CH_2_PO_3_)_2_.

Entry	Catalyst	(Mol %)	(Time/min.)	Yield (%)
1	FeCl_3_	10	120	–
2	[PVI-SO_3_H]FeCl_4_^82^	10 mg	120	48
3	CF_3_SO_3_H	10	120	15
4	H_2_SO_4_	10	120	42
5	[Py-SO_3_H]Cl^83^	10 mg	120	36
6	Fe_3_O_4_	10 mg	120	–
7	K_2_CO_3_	10	120	–
8	SSA^84,85^	10 mg	120	20
9	Et_3_N	10	120	–
10	NaOH	10	120	–
11	MIL-100(Cr)/NHEtN(CH_2_PO_3_H_2_)_2_^86^	10 mg	120	52
12	*p-*TSA	10	120	25
13	GTBSA^87^	10	120	40
14	MHMHPA^72^	10	120	32
15	Fe_3_O_4_@MIL-101(Cr)-N(CH_2_PO_3_)_2_ (this work)	20 mg	60	87

**Figure 12:**
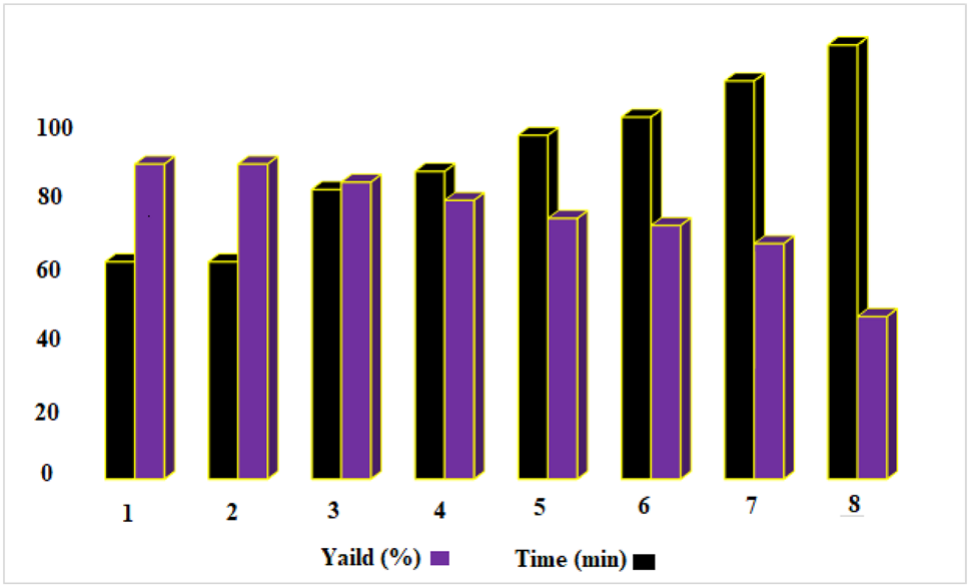
Recyclability of catalyst for the synthesis of pyrazolo[3,4-*b*]pyridine derivatives.

Finally, for the reusability of the nano-magnetic metal–organic frameworks Fe_3_O_4_@MIL-101(Cr)-N(CH_2_PO_3_)_2_ in the synthesis of pyrazolo[3,4-*b*] pyridine, this catalyst was examined in 3-(cyanoacetyl) indole (0.184 g, 1 mmol), 4-chloro benzaldehyde (0.141 g, 1 mmol) and 5-(1*H*-Indol-3-yl)-2*H*-pyrazol-3-ylamine (0.198 g, 1 mmol) (compound C2) as model reactions. The results show that the prepared catalyst can be reused up to 7 times without noticeable reduction (Fig. 12).

## Conclusion

In summary, a magnetic metal–organic frameworks Fe_3_O_4_@MIL-101(Cr)-N(CH_2_PO_3_)_2_ as nano-catalyst was designed and synthesized and identified by various techniques such as FT-IR, XRD, SEM, EDX, Mapping, BET and VSM analysis. This catalyst was tested for the preparation of novel pyrazolo[3,4-*b*]pyridines according to (CVABO) concept. The advantages of this work include, synthesis of new pyrazolo[3,4-*b*]pyridines as biological candidates, short reaction time, clean profile of the reaction and catalyst recyclability.

## Supplementary Information

Supplementary Information.

## Data Availability

The datasets used and/or analyzed during the current study available from the corresponding author on reasonable request.
