# Supplementary material for: Catalytic synthesis of new pyrazolo [3,4-b] pyridine via a cooperative vinylogous anomeric-based oxidation
Source: Sci Rep. 2022 Aug 19;12:14145. doi: 10.1038/s41598-022-17879-5 (PMC9391453; doi:10.1038/s41598-022-17879-5)
Supplement: Supplementary file 1 — Supplementary Information. [file 41598_2022_17879_MOESM1_ESM.docx]

**Catalytic synthesis of new pyrazolo [3,4-*b*] pyridine *via* a cooperative vinylogous anomeric-based oxidation**

Hassan Sepehrmansourie, ^a^ Mahmoud Zarei, ^a^* Mohammad Ali Zolfigol, ^a^* Saeed Babaee,^a^ Saeid Azizian ^b^ and Sadegh Rostamnia ^C^

^a^ Department of Organic Chemistry, Faculty of Chemistry, Bu-Ali Sina University, Hamedan 6517838683, Tel: +988138282807, Fax: +988138380709 Iran. E-Mail: mahmoud8103@yahoo.com, zolfi@basu.ac.ir & [mzolfigol@yahoo.com](mailto:mzolfigol@yahoo.com) (M. A. Zolfigol).

^b^ Department of Physical Chemistry, Faculty of Chemistry, Bu-Ali Sina University, Hamedan, 6517838683, Iran

^c^ Organic and Nano Group (ONG), Department of Chemistry Iran University of Science and Technology (IUST), PO Box 16846-13114, Tehran, Iran. E-Mail: rostamnia@iust.ac.ir

**CONTENT**

*Spectral data of pyrazolo [3,4-b] pyridine derivatives....................................................................1*

[^1^H-NMR spectrum of 3-(1H-indol-3-yl)-1H-pyrazol-5-amine. 10](#_Toc73460067)

[^13^C-NMR spectrum of 3-(1H-indol-3-yl)-1H-pyrazol-5-amine. 11](#_Toc73460068)

[^1^H-^13^C NMR (HSQC) spectrum of 3-(1H-indol-3-yl)-1H-pyrazol-5-amine. 12](#_Toc73460069)

[FT-IR spectrum of 3,6-di(1H-indol-3-yl)-4-(p-tolyl)-1H-pyrazolo[3,4-b]pyridine-5-carbonitrile (C1). 13](#_Toc73460070)

[^1^H-NMR spectrum of 3,6-di(1H-indol-3-yl)-4-(p-tolyl)-1H-pyrazolo[3,4-b]pyridine-5-carbonitrile (C1). 14](#_Toc73460071)

[^13^C-NMR spectrum of 3,6-di(1H-indol-3-yl)-4-(p-tolyl)-1H-pyrazolo[3,4-b]pyridine-5-carbonitrile (C1). 15](#_Toc73460072)

[^1^H-NMR (D_2_O exchange) spectrum of 3,6-di(1H-indol-3-yl)-4-(p-tolyl)-1H-pyrazolo[3,4-b]pyridine-5-carbonitrile (C1). 16](#_Toc73460073)

[^1^H-^13^C NMR (HSQC) spectrum of 3,6-di(1H-indol-3-yl)-4-(p-tolyl)-1H-pyrazolo[3,4-b]pyridine-5-carbonitrile (C1). 17](#_Toc73460074)

[^1^H-^1^H NMR (COSY) spectrum of 3,6-di(1H-indol-3-yl)-4-(p-tolyl)-1H-pyrazolo[3,4-b]pyridine-5-carbonitrile (C1). 18](#_Toc73460075)

[Mass spectrum of 3,6-di(1H-indol-3-yl)-4-(p-tolyl)-1H-pyrazolo[3,4-b]pyridine-5-carbonitrile (C1). 19](#_Toc73460076)

[FT-IR spectrum of 4-(4-chlorophenyl)-3,6-di(1H-indol-3-yl)-1H-pyrazolo[3,4-b]pyridine-5-carbonitrile (C2). 20](#_Toc73460077)

[^1^H-NMR spectrum of 4-(4-chlorophenyl)-3,6-di(1H-indol-3-yl)-1H-pyrazolo[3,4-b]pyridine-5-carbonitrile (C2). 21](#_Toc73460078)

[^13^C-NMR spectrum of 4-(4-chlorophenyl)-3,6-di(1H-indol-3-yl)-1H-pyrazolo[3,4-b]pyridine-5-carbonitrile (C2). 22](#_Toc73460079)

[FT-IR spectrum of 3,6-di(1H-indol-3-yl)-4-(4-methoxyphenyl)-1H-pyrazolo[3,4-b]pyridine-5-carbonitrile (C3). 23](#_Toc73460080)

[^1^H-NMR spectrum of 3,6-di(1H-indol-3-yl)-4-(4-methoxyphenyl)-1H-pyrazolo[3,4-b]pyridine-5-carbonitrile (C3). 24](#_Toc73460081)

[^13^C-NMR spectrum of 3,6-di(1H-indol-3-yl)-4-(4-methoxyphenyl)-1H-pyrazolo[3,4-b]pyridine-5-carbonitrile (C3). 25](#_Toc73460082)

[FT-IR spectrum of 4-(3,4-dimethoxyphenyl)-3,6-di(1H-indol-3-yl)-1H-pyrazolo[3,4-b]pyridine-5-carbonitrile (C4). 26](#_Toc73460083)

[^1^H-NMR spectrum of 4-(3,4-dimethoxyphenyl)-3,6-di(1H-indol-3-yl)-1H-pyrazolo[3,4-b]pyridine-5-carbonitrile (C4). 27](#_Toc73460084)

[^13^C-NMR spectrum of 4-(3,4-dimethoxyphenyl)-3,6-di(1H-indol-3-yl)-1H-pyrazolo[3,4-b]pyridine-5-carbonitrile (C4). 28](#_Toc73460085)

[FT-IR spectrum of 4-(4-bromophenyl)-3,6-di(1H-indol-3-yl)-1H-pyrazolo[3,4-b]pyridine-5-carbonitrile (C5). 29](#_Toc73460086)

[^1^H-NMR spectrum of 4-(4-bromophenyl)-3,6-di(1H-indol-3-yl)-1H-pyrazolo[3,4-b]pyridine-5-carbonitrile (C5). 30](#_Toc73460087)

[^13^C-NMR spectrum of 4-(4-bromophenyl)-3,6-di(1H-indol-3-yl)-1H-pyrazolo[3,4-b]pyridine-5-carbonitrile (C5). 31](#_Toc73460088)

[FT-IR spectrum of 3,6-di(1H-indol-3-yl)-4-(4-nitrophenyl)-1H-pyrazolo[3,4-b]pyridine-5-carbonitrile (C6). 32](#_Toc73460089)

[^1^H-NMR spectrum of 3,6-di(1H-indol-3-yl)-4-(4-nitrophenyl)-1H-pyrazolo[3,4-b]pyridine-5-carbonitrile (C6). 33](#_Toc73460090)

[^13^C-NMR spectrum of 3,6-di(1H-indol-3-yl)-4-(4-nitrophenyl)-1H-pyrazolo[3,4-b]pyridine-5-carbonitrile (C6). 34](#_Toc73460091)

[FT-IR spectrum of 3,6-di(1H-indol-3-yl)-4-phenyl-1H-pyrazolo[3,4-b]pyridine-5-carbonitrile (C7). 35](#_Toc73460092)

[^1^H-NMR spectrum of 3,6-di(1H-indol-3-yl)-4-phenyl-1H-pyrazolo[3,4-b]pyridine-5-carbonitrile (C7). 36](#_Toc73460093)

[^13^C-NMR spectrum of 3,6-di(1H-indol-3-yl)-4-phenyl-1H-pyrazolo[3,4-b]pyridine-5-carbonitrile (C7). 37](#_Toc73460094)

[FT-IR spectrum of 4-(4-hydroxyphenyl)-3,6-di(1H-indol-3-yl)-1H-pyrazolo[3,4-b]pyridine-5-carbonitrile (C8). 38](#_Toc73460095)

[^1^H-NMR spectrum of 4-(4-hydroxyphenyl)-3,6-di(1H-indol-3-yl)-1H-pyrazolo[3,4-b]pyridine-5-carbonitrile (C8). 39](#_Toc73460096)

[^13^C-NMR spectrum of 4-(4-hydroxyphenyl)-3,6-di(1H-indol-3-yl)-1H-pyrazolo[3,4-b]pyridine-5-carbonitrile (C8). 40](#_Toc73460097)

[FT-IR spectrum of 4-(3-hydroxyphenyl)-3,6-di(1H-indol-3-yl)-1H-pyrazolo[3,4-b]pyridine-5-carbonitrile (C9). 41](#_Toc73460098)

[^1^H-NMR spectrum of 4-(3-hydroxyphenyl)-3,6-di(1H-indol-3-yl)-1H-pyrazolo[3,4-b]pyridine-5-carbonitrile (C9). 42](#_Toc73460099)

[^13^C-NMR spectrum of 4-(3-hydroxyphenyl)-3,6-di(1H-indol-3-yl)-1H-pyrazolo[3,4-b]pyridine-5-carbonitrile (C9). 43](#_Toc73460100)

[FT-IR spectrum of 4-(4-fluorophenyl)-3,6-di(1H-indol-3-yl)-1H-pyrazolo[3,4-b]pyridine-5-carbonitrile (C10). 44](#_Toc73460101)

[^1^H-NMR spectrum of 4-(4-fluorophenyl)-3,6-di(1H-indol-3-yl)-1H-pyrazolo[3,4-b]pyridine-5-carbonitrile (C10). 45](#_Toc73460102)

[^13^C-NMR spectrum of 4-(4-fluorophenyl)-3,6-di(1H-indol-3-yl)-1H-pyrazolo[3,4-b]pyridine-5-carbonitrile (C10). 46](#_Toc73460103)

[FT-IR spectrum of 3,6-di(1H-indol-3-yl)-4-(4-isopropylphenyl)-1H-pyrazolo[3,4-b]pyridine-5-carbonitrile (C11). 47](#_Toc73460104)

[^1^H-NMR spectrum of 3,6-di(1H-indol-3-yl)-4-(4-isopropylphenyl)-1H-pyrazolo[3,4-b]pyridine-5-carbonitrile (C11). 48](#_Toc73460105)

[^13^C-NMR spectrum of 3,6-di(1H-indol-3-yl)-4-(4-isopropylphenyl)-1H-pyrazolo[3,4-b]pyridine-5-carbonitrile (C11). 49](#_Toc73460106)

[FT-IR spectrum of 4-(5-cyano-3,6-di(1H-indol-3-yl)-1H-pyrazolo[3,4-b]pyridin-4-yl)benzoic acid (C12). 50](#_Toc73460107)

[^1^H-NMR spectrum of 4-(5-cyano-3,6-di(1H-indol-3-yl)-1H-pyrazolo[3,4-b]pyridin-4-yl)benzoic acid (C12). 51](#_Toc73460108)

[FT-IR spectrum of 3,6-di(1H-indol-3-yl)-4-(pyridin-4-yl)-1H-pyrazolo[3,4-b]pyridine-5-carbonitrile (C13). 52](#_Toc73460109)

[^1^H-NMR spectrum of 3,6-di(1H-indol-3-yl)-4-(pyridin-4-yl)-1H-pyrazolo[3,4-b]pyridine-5-carbonitrile (C13). 53](#_Toc73460110)

[^13^C-NMR spectrum of 3,6-di(1H-indol-3-yl)-4-(pyridin-4-yl)-1H-pyrazolo[3,4-b]pyridine-5-carbonitrile (C13). 54](#_Toc73460111)

[FT-IR spectrum of 3,6-di(1H-indol-3-yl)-4-(m-tolyl)-1H-pyrazolo[3,4-b]pyridine-5-carbonitrile (C14). 55](#_Toc73460112)

[^1^H-NMR spectrum of 3,6-di(1H-indol-3-yl)-4-(m-tolyl)-1H-pyrazolo[3,4-b]pyridine-5-carbonitrile (C14). 56](#_Toc73460113)

[^13^C-NMR spectrum of 3,6-di(1H-indol-3-yl)-4-(m-tolyl)-1H-pyrazolo[3,4-b]pyridine-5-carbonitrile (C14). 57](#_Toc73460114)

[FT-IR spectrum of 3,6-di(1H-indol-3-yl)-4-(naphthalen-2-yl)-1H-pyrazolo[3,4-b]pyridine-5-carbonitrile (C15). 58](#_Toc73460115)

[^1^H-NMR spectrum of 3,6-di(1H-indol-3-yl)-4-(naphthalen-2-yl)-1H-pyrazolo[3,4-b]pyridine-5-carbonitrile (C15). 59](#_Toc73460116)

[^13^C-NMR spectrum of 3,6-di(1H-indol-3-yl)-4-(naphthalen-2-yl)-1H-pyrazolo[3,4-b]pyridine-5-carbonitrile (C15). 60](#_Toc73460117)

[FT-IR spectrum of 3,6-di(1H-indol-3-yl)-4-(naphthalen-1-yl)-1H-pyrazolo[3,4-b]pyridine-5-carbonitrile (C16). 61](#_Toc73460118)

[^1^H-NMR spectrum of 3,6-di(1H-indol-3-yl)-4-(naphthalen-1-yl)-1H-pyrazolo[3,4-b]pyridine-5-carbonitrile (C16). 62](#_Toc73460119)

[^13^C-NMR spectrum of 3,6-di(1H-indol-3-yl)-4-(naphthalen-1-yl)-1H-pyrazolo[3,4-b]pyridine-5-carbonitrile (C16). 63](#_Toc73460120)

[FT-IR spectrum of 4-(3,4-dihydroxyphenyl)-3,6-di(1H-indol-3-yl)-1H-pyrazolo[3,4-b]pyridine-5-carbonitrile (C17). 64](#_Toc73460121)

[^1^H-NMR spectrum of 4-(3,4-dihydroxyphenyl)-3,6-di(1H-indol-3-yl)-1H-pyrazolo[3,4-b]pyridine-5-carbonitrile (C17). 65](#_Toc73460122)

[^13^C-NMR spectrum of 4-(3,4-dihydroxyphenyl)-3,6-di(1H-indol-3-yl)-1H-pyrazolo[3,4-b]pyridine-5-carbonitrile (C17). 66](#_Toc73460123)

[FT-IR spectrum of 3,6-di(1H-indol-3-yl)-4-(2-methoxyphenyl)-1H-pyrazolo[3,4-b]pyridine-5-carbonitrile (C18). 67](#_Toc73460124)

[^1^H-NMR spectrum of 3,6-di(1H-indol-3-yl)-4-(2-methoxyphenyl)-1H-pyrazolo[3,4-b]pyridine-5-carbonitrile (C18). 68](#_Toc73460125)

[^13^C-NMR spectrum of 3,6-di(1H-indol-3-yl)-4-(2-methoxyphenyl)-1H-pyrazolo[3,4-b]pyridine-5-carbonitrile (C18). 69](#_Toc73460126)

[FT-IR spectrum of 4-(2-hydroxyphenyl)-3,6-di(1H-indol-3-yl)-1H-pyrazolo[3,4-b]pyridine-5-carbonitrile (C19). 70](#_Toc73460127)

[^1^H-NMR spectrum of 4-(2-hydroxyphenyl)-3,6-di(1H-indol-3-yl)-1H-pyrazolo[3,4-b]pyridine-5-carbonitrile (C19 71](#_Toc73460128)

[^13^C-NMR spectrum of 4-(2-hydroxyphenyl)-3,6-di(1H-indol-3-yl)-1H-pyrazolo[3,4-b]pyridine-5-carbonitrile (C19). 72](#_Toc73460129)

[FT-IR spectrum of 4-(3-ethoxy-4-hydroxyphenyl)-3,6-di(1H-indol-3-yl)-1H-pyrazolo[3,4-b]pyridine-5-carbonitrile (C20). 73](#_Toc73460130)

[^1^H-NMR spectrum of 4-(3-ethoxy-4-hydroxyphenyl)-3,6-di(1H-indol-3-yl)-1H-pyrazolo[3,4-b]pyridine-5-carbonitrile (C20). 74](#_Toc73460131)

[^13^C-NMR spectrum of 4-(3-ethoxy-4-hydroxyphenyl)-3,6-di(1H-indol-3-yl)-1H-pyrazolo[3,4-b]pyridine-5-carbonitrile (C20). 75](#_Toc73460132)

[FT-IR spectrum of 4,4',4''-(((1,3,5-triazine-2,4,6-triyl)tris(oxy))tris(benzene-4,1-diyl))tris(3,6-di(1H-indol-3-yl)-4,7-dihydro-1H-pyrazolo[3,4-b]pyridine-5-carbonitrile) (C21). 76](#_Toc73460133)

[^1^H-NMR spectrum of 4,4',4''-(((1,3,5-triazine-2,4,6-triyl)tris(oxy))tris(benzene-4,1-diyl))tris(3,6-di(1H-indol-3-yl)-4,7-dihydro-1H-pyrazolo[3,4-b]pyridine-5-carbonitrile) (C21). 77](#_Toc73460134)

[^13^C-NMR spectrum of 4,4',4''-(((1,3,5-triazine-2,4,6-triyl)tris(oxy))tris(benzene-4,1-diyl))tris(3,6-di(1H-indol-3-yl)-4,7-dihydro-1H-pyrazolo[3,4-b]pyridine-5-carbonitrile) (C21). 78](#_Toc73460135)

[FT-IR spectrum of 4,4'-(1,3-phenylene)bis(3,6-di(1H-indol-3-yl)-1H-pyrazolo[3,4-b]pyridine-5-carbonitrile) (C22). 79](#_Toc73460136)

[^1^H-NMR spectrum of 4,4'-(1,3-phenylene)bis(3,6-di(1H-indol-3-yl)-1H-pyrazolo[3,4-b]pyridine-5-carbonitrile) (C22). 80](#_Toc73460137)

[^13^C-NMR spectrum of 4,4'-(1,3-phenylene)bis(3,6-di(1H-indol-3-yl)-1H-pyrazolo[3,4-b]pyridine-5-carbonitrile) (C22). 81](#_Toc73460138)

**Spectral data of pyrazolo [3,4-*b*] pyridine derivatives (C1-C22)**

***5-(1H-Indol-3-yl)- 2H-pyrazol-3-ylamine***

Beige color solid, M.p.: >300 ˚C; ^1^H NMR (400 MHz, DMSO-*d_6_*): δ (ppm): 11.31 (s, 2H), 7.88 (d, *J*: 7.8 Hz, 1H), 7.67 (d, *J*: 2.5 Hz, 1H), 7.44 (d, *J*: 7.9 Hz, 1H), 7.15 (dd, *J*: 11.0, 4.1 Hz, 1H), 7.12 – 7.07 (m, 1H), 5.78 (s, 1H), 4.17 (s, 2H). ^13^C NMR 154.2, 136.2, 124.6, 122.6, 121.4, 119.7, 119.4, 111.7, 87.8.

**Spectral data of compound C1**

Beige color solid, M.p.: >300 ˚C; FT-IR: 3405, 3394, 2920, 2223, 1555; ^1^H NMR (400 MHz, DMSO-*d_6_*): δ (ppm): 14.06 (s, 1H), 11.82 (d, *J*: 2.9 Hz, 1H), 11.03 (d, *J:* 2.8 Hz, 1H), 8.54 (dd, *J:* 7.2, 1.8 Hz, 1H), 8.39 (d, *J:* 3.0 Hz, 1H), 8.09 (d, *J:* 7.9 Hz, 1H), 7.56 – 7.53 (m, 1H), 7.37-7.35 (m, 2H), 7.32 (d, *J*: 6.8 Hz, 1H), 7.29 – 7.23 (m, 4H), 7.13 – 7.09 (m, 1H), 7.08 – 7.03 (m, 1H), 5.79 (d, *J*: 2.7 Hz, 1H), 2.43 (s, 3H). ^13^C NMR (100 MHz, DMSO-*d_6_*): δ (ppm): 154.8, 152.5, 152.3, 141.5, 139.1, 136.2, 135.5, 132.4, 129.1, 128.8, 128.7, 126.1, 125.9, 122.4, 121.9, 121.5, 120.9, 120.5, 119.4, 119.4, 112.7, 112.0, 111.2, 108.1, 106.9, 98.4, 21.04.

***^1^H-NMR (D_2_O exchange) of* compound C1**

^1^H NMR (400 MHz, DMSO-*d_6_*): δ (ppm): *(****D_2_O exchange****)*: 8.46 (d, *J:* 7.7 Hz, 1H), 8.33 (s, 1H), 7.99 (d, *J:* 7.9 Hz, 1H), 7.54 (d, *J:* 7.7 Hz, 1H), 7.34 – 7.24 (m, 5H), 7.21 – 7.17 (m, 2H), 7.10 (t, *J:* 7.5 Hz, 1H), 7.03 (t, *J:* 7.5 Hz, 1H), 5.82 (s, 1H), 2.36 (s, 3H).

***^1^H-^1^H NMR (COSY) of* compound C1**

Beige color solid, M.p.: >300 ˚C; (400, 400) MHz, DMSO-*d_6_*) (14.10 14.10), (11.83 8.64), (11.75 11.75), (11.02 6.05), (11.02 11.02), (8.53 8.53), (8.53 7.45), (8.53 7.20), (8.53 8.54), (8.38 11.89), (8.08 8.08), (8.08 7.26), (8.05 7.01), (8.05 8.01), (7.54 7.87), (7.54 7.40), (7.54 7.18), (7.54 7.64), (7.38 7.71), (7.30 7.30), (7.30 6.98), (7.30 7.64), (7.26 8.78), (7.25 7.88), (7.23 8.53), (7.10 7.40), (7.09 7.64), (7.04 7.27), (7.04 8.34), (7.04 8.08), (7.04 7.04), (5.90 5.90), (5.80 11.23), (2.40 2.40).

***^1^H-^13^C NMR (HSQC) of* compound C1**

Beige color solid, M.p.: >300 ˚C; (400, 100) MHz, DMSO-*d_6_*) (8.64 122.4), (8.49 129.2), (8.19 121.4), (7.66 122.9), (7.65 112.4), (7.45 129.6), (7.42 111.7), (7.36 122.5), (7.35 129.3), (7.21 122.0), (7.21 111.6), (7.15 119.9), (5.89 126.4), (2.41 21.4).

**Spectral data of compound C2**

Beige color solid, M.p.: >300 ˚C; FT-IR: 3408, 3259, 2857, 2255, 1553. ^1^H NMR (400 MHz, DMSO-*d_6_*): δ (ppm): 14.13 (s, 1H), 11.84 (d, *J:* 3.0 Hz, 1H), 11.14 (s, 1H), 8.53 (dd, *J:* 6.7, 1.7 Hz, 1H), 8.39 (d, *J:* 2.9 Hz, 1H), 7.95 (d, *J:* 7.9 Hz, 1H), 7.57 – 7.54 (m, 1H), 7.52 – 7.44 (m, 4H), 7.33 (d, *J:* 8.1 Hz, 1H), 7.30 – 7.22 (m, 2H), 7.14 – 7.09 (m, 1H), 7.06 – 7.00 (m, 1H), 6.08 (d, *J:* 2.6 Hz, 1H). ^13^C NMR (100 MHz, DMSO-*d_6_*): δ (ppm): 154.8, 152.4, 151.0, 141.2, 136.3, 135.5, 134.5, 133.7, 131.2, 128.8, 128.1, 126.0, 125.9, 125.8, 122.4, 121.8, 121.5, 120.5, 119.4, 119.2, 112.7, 112.0, 111.3, 108.0, 106.9, 98.4.

**Spectral data of compound C3**

Beige color solid, M.p.: >300 ˚C; FT-IR: 3399, 3291, 3049, 2220, 1513. ^1^H NMR (400 MHz, DMSO-*d_6_*): δ (ppm): 14.05 (s, 1H), 11.82 (d, *J:* 2.9 Hz, 1H), 11.11 (d, *J:* 2.7 Hz, 1H), 8.55 – 8.51 (m, 1H), 8.39 (d, *J:* 2.9 Hz, 1H), 8.07 (d, *J:* 7.9 Hz, 1H), 7.55 (dd, *J:* 6.6, 1.3 Hz, 1H), 7.41-7.39 (m, 2H), 7.32 (d, *J*: 8.1 Hz, 1H), 7.28 – 7.21 (m, 2H), 7.14 – 7.08 (m, 1H), 7.07 – 7.03 (m, 1H), 6.99 (d, *J:* 8.7 Hz, 2H), 5.89 (d, *J:* 2.7 Hz, 1H), 3.84 (s, 3H). ^13^C NMR (100 MHz, DMSO-*d_6_*): δ (ppm): 160.2, 154.9, 152.4, 152.3, 141.5, 136.2, 135.5, 130.8, 128.7, 127.2, 126.1, 126.0, 125.9, 122.4, 121.9, 121.5, 120.9, 120.5, 119.4, 113.6, 112.8, 111.9, 111.2, 108.1, 107.0, 98.6, 55.3.

**Spectral data of compound C4**

Beige color solid, M.p.: 239-241 ˚C; FT-IR: 3395, 3109, 2915, 2220, 1515. ^1^H NMR (400 MHz, DMSO-*d_6_*): δ (ppm): 14.07 (s, 1H), 11.82 (d, *J:* 3.0 Hz, 1H), 11.13 (d, *J:* 2.7 Hz, 1H), 8.53 (dd, *J:* 7.3, 1.9 Hz, 1H), 8.40 (d, *J:* 2.9 Hz, 1H), 8.00 (d, *J:* 7.9 Hz, 1H), 7.56 (dd, *J:* 6.9, 1.8 Hz, 1H), 7.34 (d, *J:* 8.0 Hz, 1H), 7.29 – 7.22 (m, 2H), 7.14 – 7.09 (m, 2H), 7.05-7.02 (m, 2H), 6.92 (d, *J:* 2.0 Hz, 1H), 6.03 (d, *J:* 2.7 Hz, 1H), 3.83 (s, 3H), 3.33 (s, 3H). ^13^C NMR (100 MHz, DMSO-*d_6_*): δ (ppm): 155.0, 152.4, 152.2, 149.8, 148.1, 141.5, 136.2, 135.4, 128.7, 126.9, 126.1, 125.9, 125.9, 122.4, 122.1, 121.9, 121.4, 120.5, 120.5, 119.6, 119.4, 113.6, 112.8, 111.9, 111.2, 108.1, 107.15, 98.54, 55.5, 55.0.

**Spectral data of compound C5**

Beige color solid, M.p.: >300 ˚C; FT-IR: 3409, 3255, 3056, 2224, 1587. ^1^H NMR (400 MHz, DMSO-*d_6_*): δ (ppm): 14.13 (s, 1H), 11.83 (d, *J:* 2.9 Hz, 1H), 11.15 (d, *J:* 2.7 Hz, 1H), 8.54 (d, *J:* 7.0 Hz, 1H), 8.39 (d, *J:* 3.0 Hz, 1H), 7.94 (d, *J:* 7.9 Hz, 1H), 7.60 (d, *J:* 8.4 Hz, 2H), 7.57 – 7.53 (m, 1H), 7.43 (d, *J:* 8.5 Hz, 2H), 7.34 (d, *J:* 8.1 Hz, 1H), 7.29 – 7.22 (m, 2H), 7.13 (t, *J:* 8.2 Hz, 1H), 7.03 (t, *J:* 7.5 Hz, 1H), 6.09 (d, *J:* 2.7 Hz, 1H). ^13^C NMR (100 MHz, DMSO-*d_6_*): δ (ppm): 154.9, 152.4, 151.0, 141.2, 136.2, 135.5, 134.1, 131.4, 131.0, 128.8, 126.0, 125.9, 125.6, 123.1, 122.5, 121.8, 121.6, 120.6, 119.4, 119.2, 112.7, 112.0, 111.3, 108.0, 106.9, 98.3.

**Spectral data of compound C6**

Beige color solid, M.p.: >300 ˚C; FT-IR: 3444, 3386, 3272, 2217, 1533. ^1^H NMR (400 MHz, DMSO-*d_6_*): δ (ppm): 14.22 (s, 1H), 11.87 (d, *J:* 3.0 Hz, 1H), 11.04 (d, *J:* 2.7 Hz, 1H), 8.56 (dd, *J:* 7.9, 1.6 Hz, 1H), 8.41 (d, *J:* 3.0 Hz, 1H), 8.17 (d, *J:* 8.7 Hz, 2H), 7.78 (d, *J:* 8.0 Hz, 1H), 7.74 (d, *J:* 8.7 Hz, 2H), 7.57 (dd, *J:* 6.7, 1.9 Hz, 1H), 7.31 – 7.27 (m, 2H), 7.27 – 7.23 (m, 1H), 7.10 – 7.05 (m, 1H), 7.01 – 6.96 (m, 1H), 6.17 – 6.13 (m, 1H). ^13^C NMR (100 MHz, DMSO-*d_6_*): δ (ppm): 154.9, 152.4, 150.0, 147.6, 141.2, 141.0, 136.3, 135.3, 130.9, 128.8, 126.0, 126.0, 125.6, 122.8, 122.5, 121.8, 121.5, 120.6, 120.1, 119.4, 118.9, 112.7, 112.0, 111.2, 108.1, 106.6, 97.8.

**Spectral data of compound C7**

Yellow color solid, M.p.: >300 ˚C; FT-IR: 3410, 3385, 3170, 2221, 1526. ^1^H NMR (400 MHz, DMSO-*d_6_*): δ (ppm): 14.10 (s, 1H), 11.85 (s, 1H), 11.04 (d, *J:* 2.7 Hz, 1H), 8.54 (dd, *J:* 6.4, 1.4 Hz, 1H), 8.39 (s, 1H), 8.08 (d, *J:* 7.8 Hz, 1H), 7.58 – 7.52 (m, 2H), 7.50 – 7.43 (m, 4H), 7.30 – 7.22 (m, 3H), 7.13 – 7.08 (m, 1H), 7.07 – 7.02 (m, 1H), 5.78 (d, *J:* 2.6 Hz, 1H). ^13^C NMR (100 MHz, DMSO-*d_6_*): δ (ppm): 154.8, 152.3, 136.2, 135.5, 135.2, 129.5, 129.2, 128.7, 128.2, 126.0, 125.8, 125.7, 122.4, 121.9, 121.5, 120.8, 120.5, 119.4, 119.2, 112.7, 112.0, 111.2, 108.0, 98.4.

**Spectral data of compound C8**

Yellow color solid, M.p.: >300 ˚C; FT-IR: 3409, 3209, 2923, 2219, 1590. ^1^H NMR (400 MHz, DMSO-*d_6_*): δ (ppm): 14.04 (s, 1H), 11.82 (d, *J:* 2.9 Hz, 1H), 11.16 (d, *J:* 2.8 Hz, 1H), 9.95 (s, 1H), 8.54 (dd, *J:* 7.2, 2.0 Hz, 1H), 8.39 (d, *J:* 2.7 Hz, 1H), 8.13 (d, *J:* 7.8 Hz, 1H), 7.56 (dd, *J:* 6.8, 1.7 Hz, 1H), 7.35 – 7.28 (m, 3H), 7.27 – 7.21 (m, 2H), 7.12 – 7.10 (m, 1H), 7.07 – 6.59 (m, 1H), 6.82 (d, *J:* 8.4 Hz, 2H), 5.96 (d, *J:* 2.6 Hz, 1H). ^13^C NMR (100 MHz, DMSO-*d_6_*): δ (ppm): 162.3, 158.6, 154.9, 152.7, 152.4, 141.6, 136.2, 135.5, 130.8, 128.7, 126.1, 125.8, 125.6, 122.4, 121.9, 121.5, 121.0, 120.5, 119.6, 119.4, 114.9, 112.8, 111.9, 111.2, 108.1, 107.1, 98.7.

**Spectral data of compound C9**

Beige color solid, M.p.: >300 ˚C; FT-IR: 3330, 3054, 2931, 2216, 1654. ^1^H NMR (400 MHz, DMSO-*d_6_*): δ (ppm): 14.07 (s, 1H), 11.86 (s, 1H), 11.14 (d, *J:* 2.7 Hz, 1H), 9.85 (s, 1H), 8.55 (dd, *J:* 7.1, 1.9 Hz, 1H), 8.40 (s, 1H), 8.21 (d, *J:* 7.7 Hz, 1H), 7.59 – 7.55 (m, 1H), 7.32 (d, *J:* 7.9 Hz, 2H), 7.26 – 7.18 (m, 2H), 7.15 – 7.06 (m, 2H), 6.99 (dd, *J:* 8.3, 2.5 Hz, 1H), 6.92 – 6.87 (m, 2H), 5.88 (d, *J:* 2.6 Hz, 1H). ^13^C NMR (100 MHz, DMSO-*d_6_*): δ (ppm): 162.2, 157.34, 154.7, 152.3, 141.6, 136.7, 136.2, 135.6, 129.6, 128.7, 126.1, 125.8, 125.7, 123.2, 122.4, 122.2, 121.9, 121.5, 121.2, 120.9, 120.5, 119.7, 119.5, 119.2, 116.5, 115.8, 112.74, 112.40, 112.0, 111.2, 107.8, 107.0, 98.3.

**Spectral data of compound C10**

Beige color solid, M.p.: >300 ˚C; FT-IR: 3393, 3261, 3059, 2225, 1597. ^1^H NMR (400 MHz, DMSO-*d_6_*): δ (ppm): 14.12 (s, 1H), 11.83 (d, *J:* 3.0 Hz, 1H), 11.13 (d, *J:* 2.7 Hz, 1H), 8.54 (dd, *J:* 7.3, 1.9 Hz, 1H), 8.40 (d, *J:* 3.0 Hz, 1H), 7.98 (d, *J:* 7.9 Hz, 1H), 7.57 – 7.51 (m, 3H), 7.33 (d, *J:* 8.0 Hz, 1H), 7.28 – 7.22 (m, 4H), 7.14 – 7.09 (m, 1H), 7.06 – 7.01 (m, 1H), 6.00 (d, *J:* 2.7 Hz, 1H). ^13^C NMR (100 MHz, DMSO-*d_6_*): δ (ppm): 163.9, 161.5, 154.8, 152.4, 151.3, 141.3, 136.3, 135.5, 131.7, 131.6, 131.3, 128.7, 126.0, 125.9, 125.7, 122.4, 121.9, 121.5, 120.6, 120.5, 119.4, 119.2, 115.2, 115.0, 112.7, 112.0, 111.30, 108.25, 106.9, 98.6.

**Spectral data of compound C11**

Yellow color solid, M.p.: >300 ˚C; FT-IR: 3395, 3292, 2958, 2223, 1569. ^1^H NMR (400 MHz, DMSO-*d_6_*): δ (ppm): 14.09 (s, 1H), 11.83 (d, *J:* 3.0 Hz, 1H), 10.96 (d, *J:* 2.7 Hz, 1H), 8.54 (dd, *J:* 6.7, 1.7 Hz, 1H), 8.40 (d, *J:* 2.9 Hz, 1H), 8.02 (d, *J:* 7.9 Hz, 1H), 7.56 (dd, *J:* 6.8, 1.7 Hz, 1H), 7.38 (d, *J:* 8.3 Hz, 2H), 7.32 (d, *J:* 7.9 Hz, 1H), 7.29 – 7.23 (m, 4H), 7.12 – 7.07 (m, 1H), 7.04 – 7.00 (m, 1H), 5.82 (d, *J:* 2.7 Hz, 1H), 2.95 (hept, *J:* 6.9 Hz, 1H), 1.28 (d, *J:* 6.9 Hz, 6H). ^13^C NMR (100 MHz, DMSO-*d_6_*): δ (ppm): 154.9, 152.49, 152.4, 150.0, 141.5, 136.2, 135.6, 132.4, 129.2, 128.7, 126.1, 126.0, 125.9, 125.6, 122.4, 121.9, 121.4, 120.8, 120.5, 119.4, 119.4, 112.8, 112.0, 111.2, 108.2, 107.1, 98.33, 33.36, 23.8.

**Spectral data of compound C12**

Yellow color solid, M.p.: >300 ˚C; FT-IR: 3413, 3252, 2672, 2226, 1698. ^1^H NMR (400 MHz, DMSO-*d_6_*): δ (ppm): 14.17 (s, 1H), 13.16 (s, 1H), 11.86 (s, 1H), 11.07 (s, 1H), 8.56 (d, *J:* 7.4 Hz, 1H), 8.41 (d, *J:* 2.9 Hz, 1H), 8.02-7.96 (m, 3H), 7.61 (d, *J:* 7.9 Hz, 2H), 7.56 (d, *J:* 7.5 Hz, 1H), 7.31-7.23 (m, 3H), 7.11 (t, *J:* 7.5 Hz, 1H), 7.04 (t, *J:* 7.5 Hz, 1H), 5.92 (s, 1H).

**Spectral data of compound C13**

Beige color solid; M.p.: >300 ˚C; FT-IR: 3386, 3284, 3045, 2216, 1587. ^1^H NMR (400 MHz, DMSO-*d_6_*): δ (ppm): 14.23 (s, 1H), 11.88 (s, 1H), 11.14 (d, *J:* 2.7 Hz, 1H), 8.62 – 8.58 (m, 2H), 8.57 – 8.53 (m, 1H), 8.40 (s, 1H), 7.89 (d, *J:* 7.9 Hz, 1H), 7.58 – 7.55 (m, 1H), 7.51 – 7.48 (m, 2H), 7.32 (d, *J:* 8.1 Hz, 1H), 7.30 – 7.23 (m, 2H), 7.13 – 7.09 (m, 1H), 7.05 – 7.01 (m, 1H), 6.17 (d, *J:* 2.6 Hz, 1H). ^13^C NMR (100 MHz, DMSO-*d_6_*): δ (ppm): 154.8, 152.5, 149.3, 149.2, 142.6, 140.9, 136.3, 135.4, 128.8, 126.0, 126.0, 125.4, 123.9, 122.5, 121.8, 121.6, 120.6, 120.3, 119.4, 118.8, 112.6, 112.0, 111.3, 107.7, 106.7, 97.7.

**Spectral data of compound C14**

Yellow color solid, M.p.: >300 ˚C; FT-IR: 3394, 3267, 3052, 2222, 1524. ^1^H NMR (400 MHz, DMSO-*d_6_*): δ (ppm): 14.12 (s, 1H), 11.87 (s, 1H), 11.08 (d, *J:* 2.6 Hz, 1H), 8.56 (dd, *J:* 6.7, 2.1 Hz, 1H), 8.41 (s, 1H), 8.02 (d, *J:* 7.9 Hz, 1H), 7.56 (dd, *J:* 6.9, 2.1 Hz, 1H), 7.34 – 7.29 (m, 4H), 7.27 – 7.22 (m, 3H), 7.11 (t, *J:* 7.5 Hz, 1H), 7.04 (t, *J:* 7.4 Hz, 1H), 5.89 (d, *J:* 2.5 Hz, 1H), 2.19 (s, 3H). ^13^C NMR (100 MHz, DMSO-*d_6_*): δ (ppm): 154.8, 152.4, 141.4, 137.4, 136.2, 135.5, 134.9, 130.0, 129.7, 128.7, 128.1, 126.6, 126.1, 126.0, 125.7, 122.4, 121.9, 121.4, 120.7, 120.5, 119.3, 119.3, 112.7, 111.9, 111.1, 109.4, 108.19, 106.98, 98.2, 20.7.

**Spectral data of compound C15**

Beige color solid, M.p.: >300 ˚C; FT-IR: 3399, 3271, 3109, 2225, 1586. ^1^H NMR (400 MHz, DMSO-*d_6_*): δ (ppm): 14.15 (s, 1H), 11.84 (d, *J:* 3.0 Hz, 1H), 10.78 (d, *J:* 2.7 Hz, 1H), 8.58 (dd, *J:* 7.7, 1.7 Hz, 1H), 8.42 (d, *J:* 2.9 Hz, 1H), 8.14 (s, 1H), 8.07– 8.03 (m, 2H), 7.95 (d, *J:* 8.5 Hz, 1H), 7.89 (d, *J:* 8.1 Hz, 1H), 7.67– 7.63 (m, 1H), 7.61 – 7.53 (m, 3H), 7.29 – 7.25 (m, 2H), 7.18 – 7.15 (m, 1H), 7.07– 6.99 (m, 2H), 5.83 (d, *J:* 2.7 Hz, 1H). ^13^C NMR (100 MHz, DMSO-*d_6_*): δ (ppm): 154.9, 152.4, 152.2, 142.2, 141.5, 136.3, 135.4, 132.9, 132.7, 132.2, 128.8, 128.3, 127.8, 127.7, 127.1, 126.6, 126.6, 126.1, 125.8, 125.5, 122.4, 121.9, 121.5, 120.8, 120.5, 119.4, 112.77, 112.03, 111.1, 108.1, 107.0, 98.7.

**Spectral data of compound C16**

Yellow color solid, M.p.: >300 ˚C; FT-IR: 3393, 3247, 2925, 2217, 1655. ^1^H NMR (400 MHz, DMSO-*d_6_*): δ (ppm): 14.19 (s, 1H), 11.93 (s, 1H), 10.79 (d, *J:* 2.7 Hz, 1H), 8.66 – 8.62 (m, 1H), 8.39 (s, 1H), 8.17 (d, *J:* 4.7 Hz, 1H), 8.10-8.06 (m, 2H), 7.65 (d, *J:* 4.0 Hz, 2H), 7.58 – 7.55 (m, 1H), 7.53 (t, *J:* 7.4 Hz, 1H), 7.46 (d, *J:* 8.4 Hz, 1H), 7.39 (t, *J:* 7.4 Hz, 1H), 7.30 – 7.25 (m, 2H), 7.15 (d, *J:* 7.9 Hz, 1H), 7.03 (t, *J:* 7.0 Hz, 1H), 6.98 (t, *J:* 7.3 Hz, 1H), 5.31 (d, *J:* 2.7 Hz, 1H). ^13^C NMR (100 MHz, DMSO-*d_6_*): δ (ppm): 154.7, 152.5, 150.7, 141.5, 136.3, 135.4, 133.4, 132.8, 130.8, 129.7, 128.7, 128.3, 127.2, 127.1, 126.4, 126.1, 125.6, 125.3, 124.6, 124.4, 122.5, 122.0, 121.5, 121.1, 120.6, 119.3, 119.02, 112.69, 112.0, 111.0, 109.2, 106.7, 99.5.

**Spectral data of compound C17**

Yellow color solid, M.p.: >300 ˚C; FT-IR: 3387, 3336, 3056, 2218, 1580. ^1^H NMR (400 MHz, DMSO-*d_6_*): δ (ppm): 14.01 (s, 1H), 11.80 (s, 1H), 11.21 (s, 1H), 9.39 (s, 2H), 8.53 (dd, *J:* 6.8, 1.7 Hz, 1H), 8.38 (s, 1H), 8.23 (d, *J:* 7.7 Hz, 1H), 7.55 (d, *J:* 6.9 Hz, 1H), 7.32 (d, *J:* 7.4 Hz, 1H), 7.29 – 7.22 (m, 2H), 7.15 – 7.07 (m, 2H), 6.87 (d, *J:* 2.1 Hz, 1H), 6.83 (d, *J:* 8.1 Hz, 1H), 6.74 (dd, *J:* 8.0, 2.1 Hz, 1H), 5.96 (s, 1H). ^13^C NMR (100 MHz, DMSO-*d_6_*): δ (ppm): 154.8, 152.8, 152.4, 146.8, 145.2, 141.8, 136.2, 135.6, 128.6, 126.2, 126.1, 125.8, 122.4, 121.9, 121.5, 121.3, 120.6, 120.4, 119.5, 119.4, 116.4, 115.4, 112.8, 111.9, 111.2, 107.9, 107.12, 98.77.

**Spectral data of compound C18**

Beige color solid, M.p.: >300 ˚C; FT-IR: 3396, 3262, 2932, 2220, 1655. ^1^H NMR (400 MHz, DMSO-*d_6_*): δ (ppm): 14.02 (s, 1H), 11.82 (d, *J:* 2.8 Hz, 1H), 11.02 (d, *J:* 2.7 Hz, 1H), 8.56 – 8.53 (m, 1H), 8.38 (d, *J:* 2.6 Hz, 1H), 8.04 (d, *J:* 7.8 Hz, 1H), 7.56 (dd, *J:* 6.9, 1.8 Hz, 1H), 7.51 (dd, *J:* 7.9, 2.1 Hz, 1H), 7.39 (dd, *J:* 7.5, 1.7 Hz, 1H), 7.31 (d, *J:* 8.1 Hz, 1H), 7.28-7.22 (m, 2H), 7.14 – 7.08 (m, 2H), 7.04 (d, *J:* 8.2 Hz, 2H), 5.93 (d, *J:* 2.6 Hz, 1H), 3.34 (s, 3H). ^13^C NMR (100 MHz, DMSO-*d_6_*): δ (ppm): (156.3, 154.7, 152.3, 149.5, 141.7, 136.2, 135.5, 131.3, 130.3, 128.5, 126.0, 126.0, 124.8, 124.0, 122.4, 121.8, 121.5, 120.6, 120.5, 120.4, 119.4, 119.1, 112.7, 112.0, 111.6, 111.2, 108.75, 106.99, 99.1, 55.3.

**Spectral data of compound C19**

Beige color solid, M.p.: >300 ˚C; FT-IR: 3365, 3213, 2932, 2220, 2658. ^1^H NMR (400 MHz, DMSO-*d_6_*): δ (ppm): 14.00 (s, 1H), 11.85 (s, 1H), 11.07 (d, *J:* 2.7 Hz, 1H), 9.95 (s, 1H), 8.55 (dd, *J:* 6.5, 1.5 Hz, 1H), 8.37 (s, 1H), 8.22 – 8.19 (m, 1H), 7.56 (dd, *J:* 7.0, 1.6 Hz, 1H), 7.40 (t, *J:* 7.9 Hz, 1H), 7.31 – 7.23 (m, 4H), 7.13 – 7.05 (m, 2H), 6.96 (d, *J:* 8.7 Hz, 1H), 6.92 (d, *J:* 7.5 Hz, 1H), 5.95 (d, *J:* 2.6 Hz, 1H). ^13^C NMR (100 MHz, DMSO-*d_6_*): δ (ppm): 162.3, 154.8, 154.6, 152.4, 150.3, 141.9, 136.2, 135.7, 130.9, 130.2, 128.4, 126.1, 125.9, 125.0, 123.0, 122.4, 121.9, 121.5, 121.2, 120.5, 119.4, 119.2, 118.9, 115.8, 112.7, 112.0, 111.22, 108.81, 107.2, 99.4.

**Spectral data of compound C20**

Yellow color solid, M.p.: >300 ˚C; FT-IR: 3400, 3186, 2223, 1539. ^1^H NMR (400 MHz, DMSO-*d_6_*): δ (ppm): 14.04 (s, 1H), 11.82 (d, *J:* 3.0 Hz, 1H), 11.18 (d, *J:* 2.8 Hz, 1H), 9.44 (s, 1H), 8.53 (d, *J:* 7.5 Hz, 1H), 8.39 (d, *J:* 2.9 Hz, 1H), 8.07 (d, *J:* 7.8 Hz, 1H), 7.55 (d, *J:* 6.9 Hz, 1H), 7.33 (d, *J:* 8.1 Hz, 1H), 7.30 – 7.22 (m, 2H), 7.14 – 7.10 (m, 1H), 7.08– 7.04 (m, 1H), 6.95 – 6.92 (m, 2H), 6.85 (d, *J:* 8.6 Hz, 1H), 6.04 (d, *J:* 2.7 Hz, 1H), 4.14(q, *J:* 7.0 Hz, 2H), 1.09 (t, *J:* 7.0 Hz, 3H). ^13^C NMR (100 MHz, DMSO-*d_6_*): δ (ppm): 154.9, 152.6, 152.4, 148.1, 146.1, 141.6, 136.2, 135.5, 128.7, 126.1, 126.0, 125.8, 125.5, 122.3, 121.9, 121.4, 120.7, 120.4, 119.7, 119.4, 115.2, 115.1, 112.8, 111.9, 111.2, 108.1, 107.17, 98.67, 63.6, 14.3

**Spectral data of compound C21**

Yellow color solid, M.p.: >300 ˚C; FT-IR: 3409, 3287, 3051, 2219, 1650. ^1^H NMR (400 MHz, DMSO-*d_6_*): δ (ppm): 14.04 (s, 3H), 11.82 (s, 3H), 11.16 (d, *J:* 2.8 Hz, 3H), 9.96 (s, 3H), 8.54 (d, *J:* 7.1 Hz, 3H), 8.39 (d, *J:* 2.5 Hz, 3H), 8.13 (d, *J:* 7.4 Hz, 3H), 7.57 – 7.54 (m, 3H), 7.35 – 7.30 (m, 5H), 7.29 – 7.27 (m, 4H), 7.26 – 7.21 (m, 5H), 7.13 (t, *J:* 7.1 Hz, 3H), 7.07 (t, *J:* 7.4 Hz, 3H), 6.82 (d, *J:* 8.2 Hz, 6H), 5.96 (d, *J:* 2.7 Hz, 3H), 4.54 – 4.37 (m, 3H). ^13^C NMR (100 MHz, DMSO-*d_6_*): δ (ppm): 158.6, 154.9, 152.7, 152.4, 141.6, 136.5, 136.2, 135.5, 135.4, 130.8, 128.6, 126.1, 125.8, 125.6, 123.3, 122.4, 122.3, 121.9, 121.5, 121.0, 120.9, 120.5, 119.6, 119.4, 114.9, 112.8, 112.4, 111.9, 111.2, 108.1, 107.0, 98.7, 56.0.

**Spectral data of compound C22**

Yellow color solid, M.p.: >300 ˚C; FT-IR: 3394, 3289, 2923, 2216, 1589. ^1^H NMR (400 MHz, DMSO-*d_6_*): δ (ppm): 14.28 (s, 2H), 11.95 (d, *J:* 2.8 Hz, 2H), 11.07 (d, *J:* 2.9 Hz, 2H), 8.71 – 8.64 (m, 3H), 8.53 (d, *J:* 2.4 Hz, 2H), 8.23 – 8.18 (m, 2H), 7.66 – 7.62 (m, 2H), 7.38 – 7.29 (m, 9H), 7.18 – 7.13 (m, 4H), 6.45 (d, *J:* 2.6 Hz, 2H). ^13^C NMR (100 MHz, DMSO-*d_6_*): δ (ppm): 155.1, 152.5, 151.1, 142.5, 142.2, 141.6, 136.3, 135.6, 135.0, 131.3, 129.8, 129.8, 128.9, 127.8, 127.5, 126.0, 125.8, 122.5, 121.9, 121.4, 120.6, 120.0, 119.4, 114.1, 112.8, 112.0, 111.35, 107.55, 106.3, 98.5.


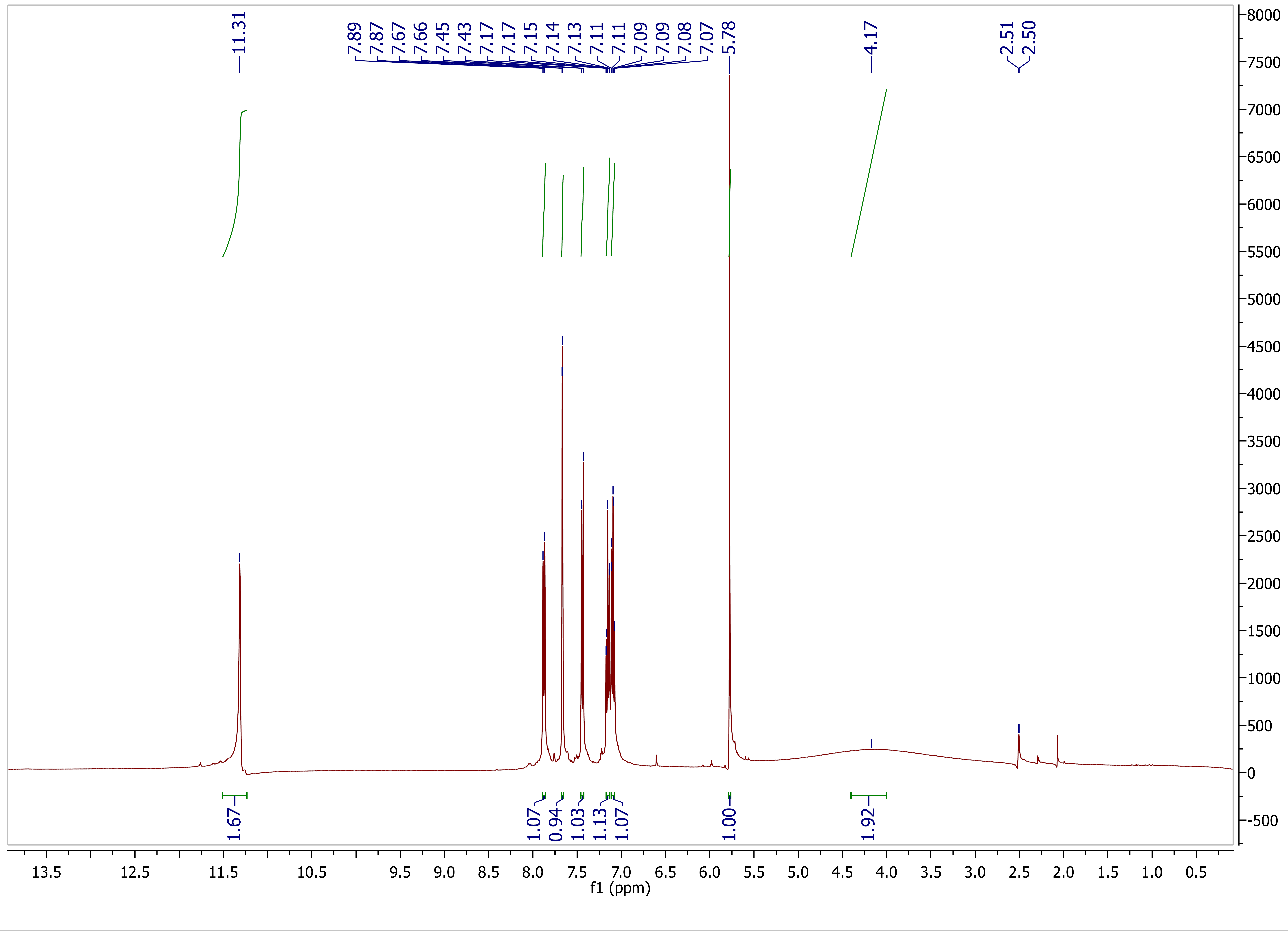


# ^1^H-NMR spectrum of 3-(1H-indol-3-yl)-1H-pyrazol-5-amine.


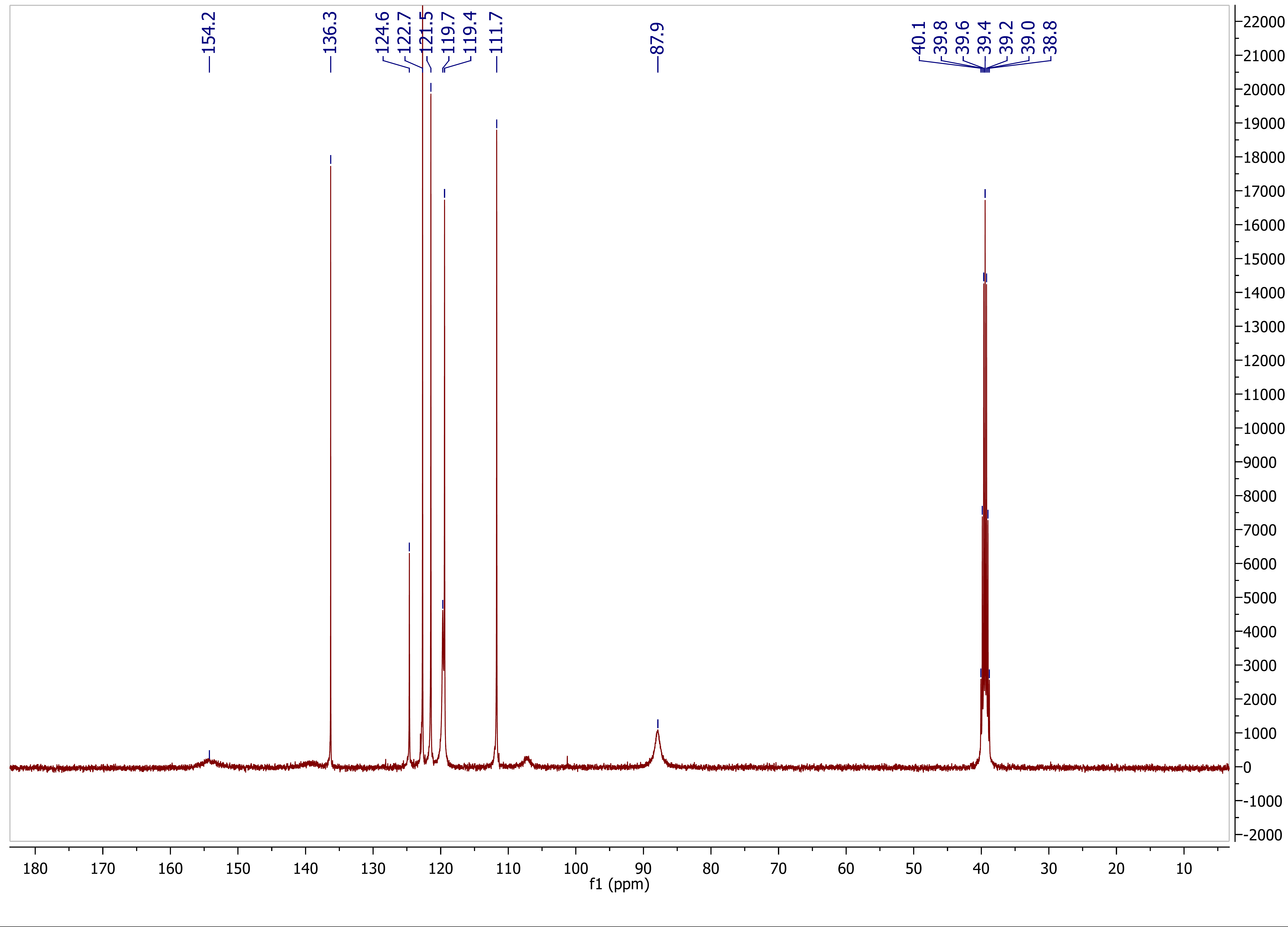


# ^13^C-NMR spectrum of 3-(1H-indol-3-yl)-1H-pyrazol-5-amine.

***
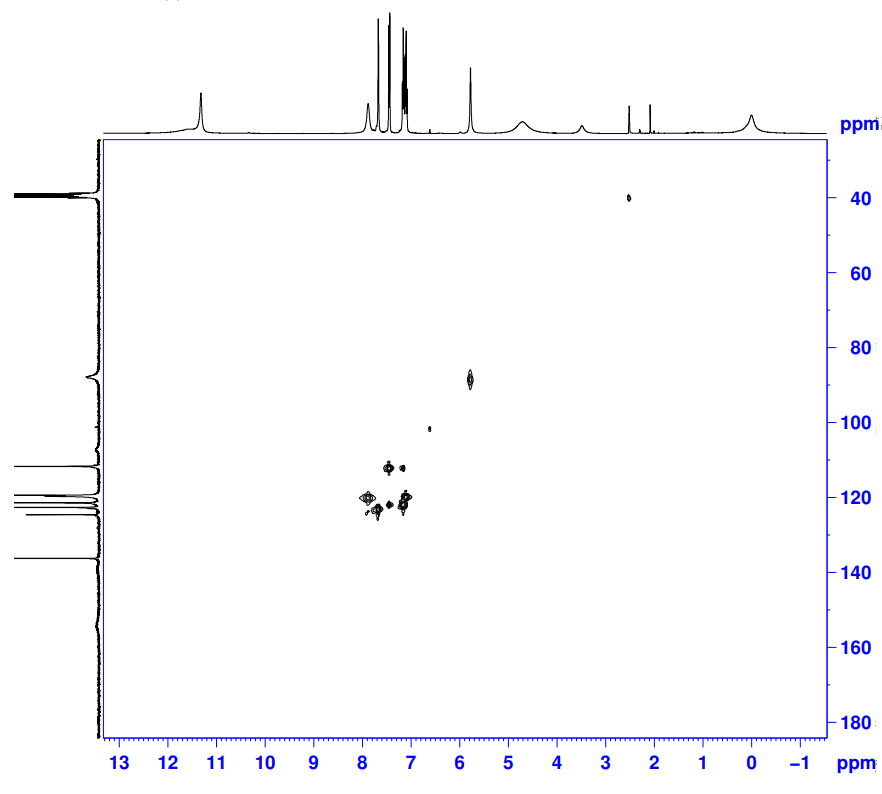
***

# ^1^H-^13^C NMR (HSQC) spectrum of 3-(1H-indol-3-yl)-1H-pyrazol-5-amine.

***
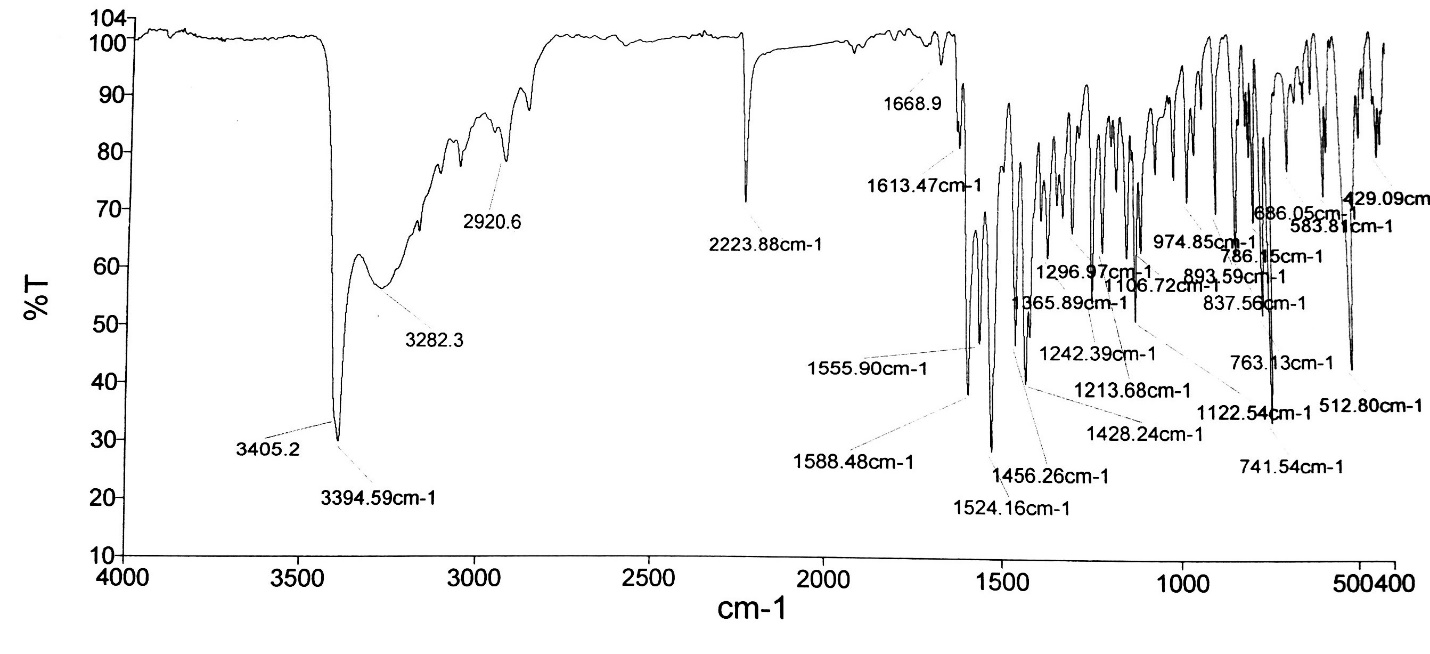
***

# FT-IR spectrum of 3,6-di(1H-indol-3-yl)-4-(p-tolyl)-1H-pyrazolo[3,4-b]pyridine-5-carbonitrile (C1).

***
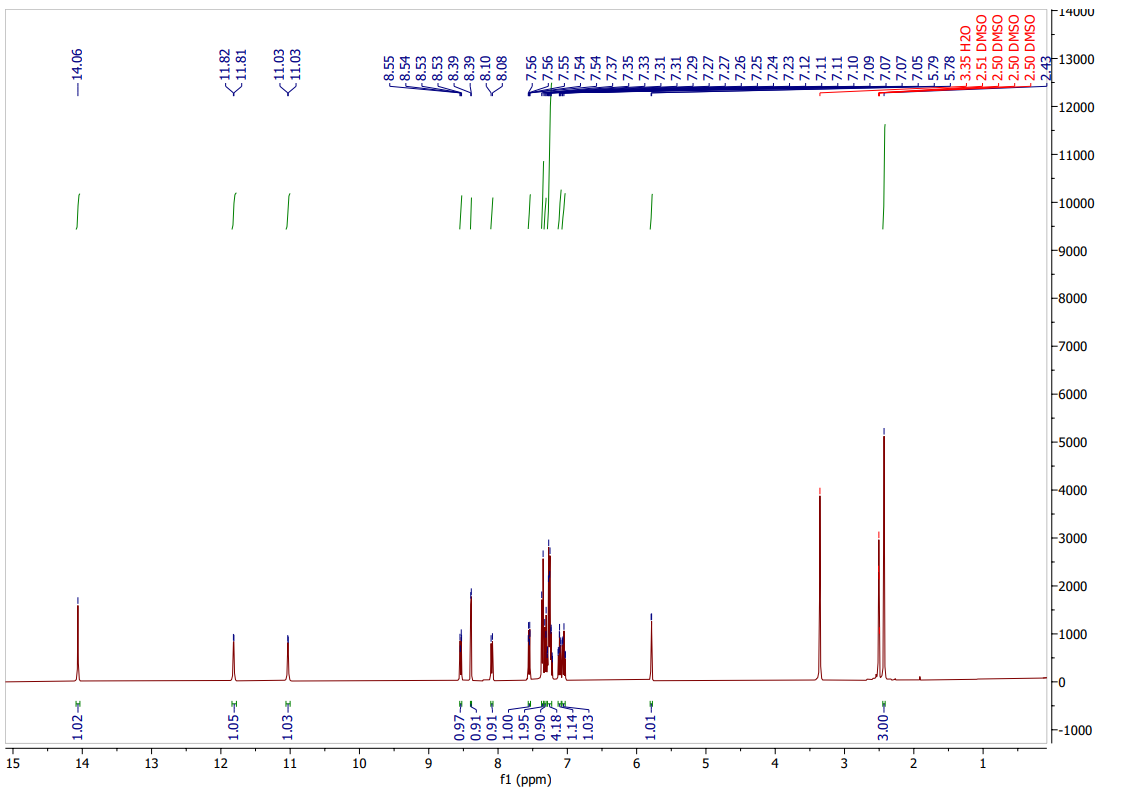
***

# ^1^H-NMR spectrum of 3,6-di(1H-indol-3-yl)-4-(p-tolyl)-1H-pyrazolo[3,4-b]pyridine-5-carbonitrile (C1).

***
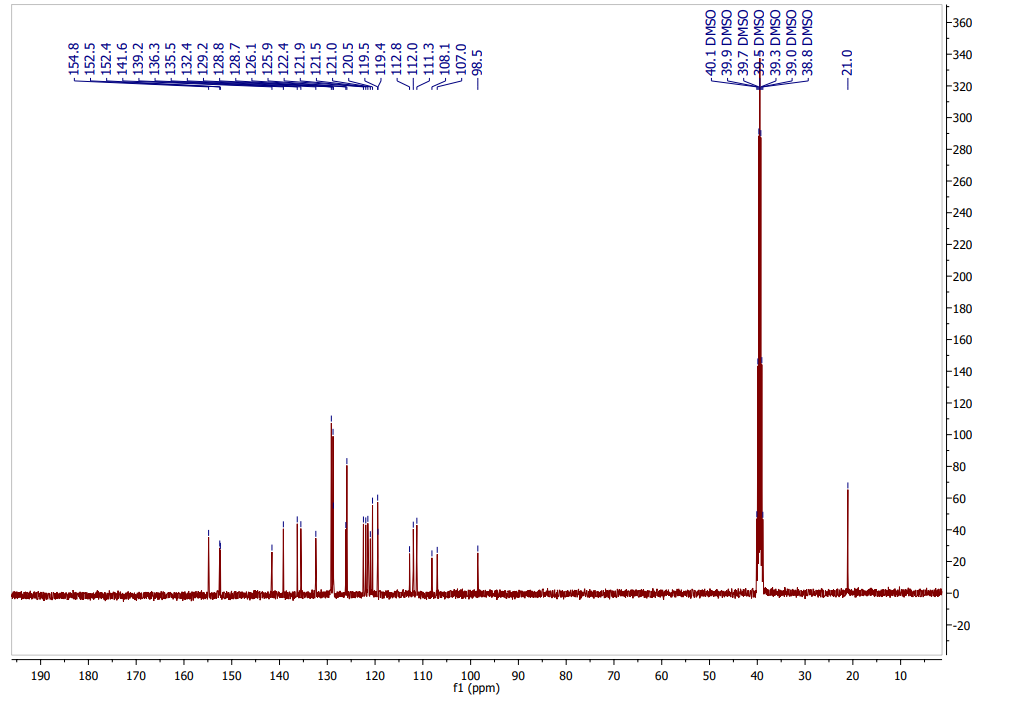
***

# ^13^C-NMR spectrum of 3,6-di(1H-indol-3-yl)-4-(p-tolyl)-1H-pyrazolo[3,4-b]pyridine-5-carbonitrile (C1).

***
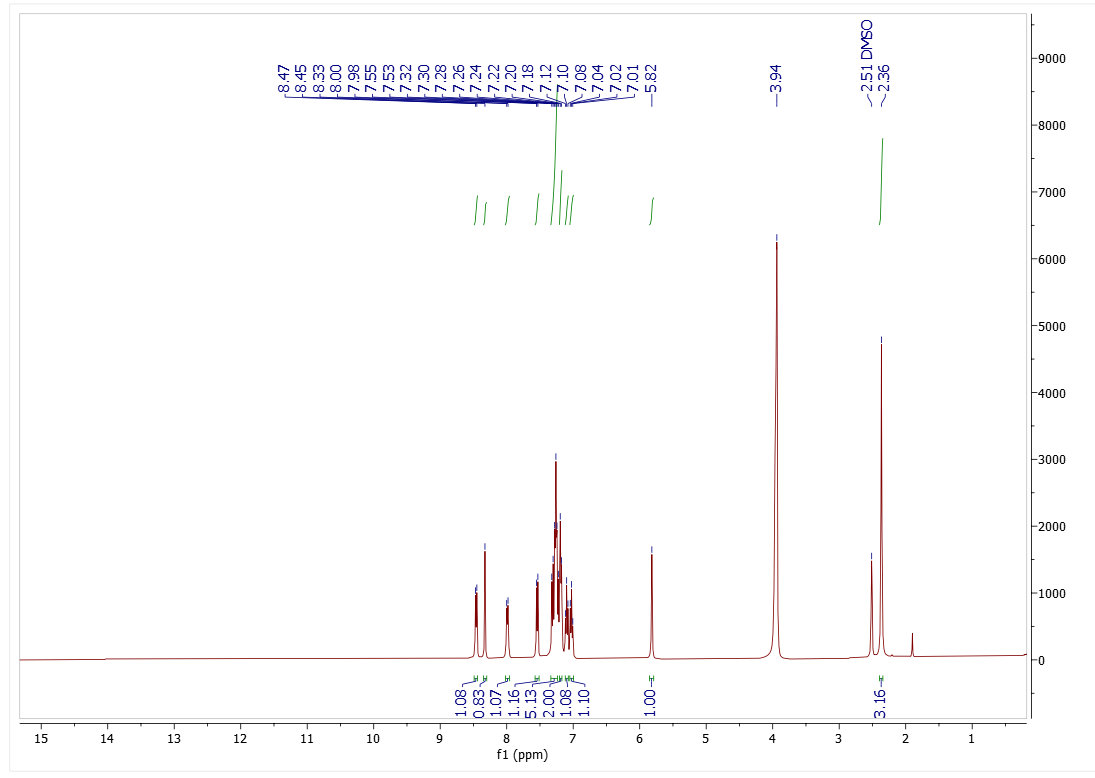
***

# ^1^H-NMR (D_2_O exchange) spectrum of 3,6-di(1H-indol-3-yl)-4-(p-tolyl)-1H-pyrazolo[3,4-b]pyridine-5-carbonitrile (C1).


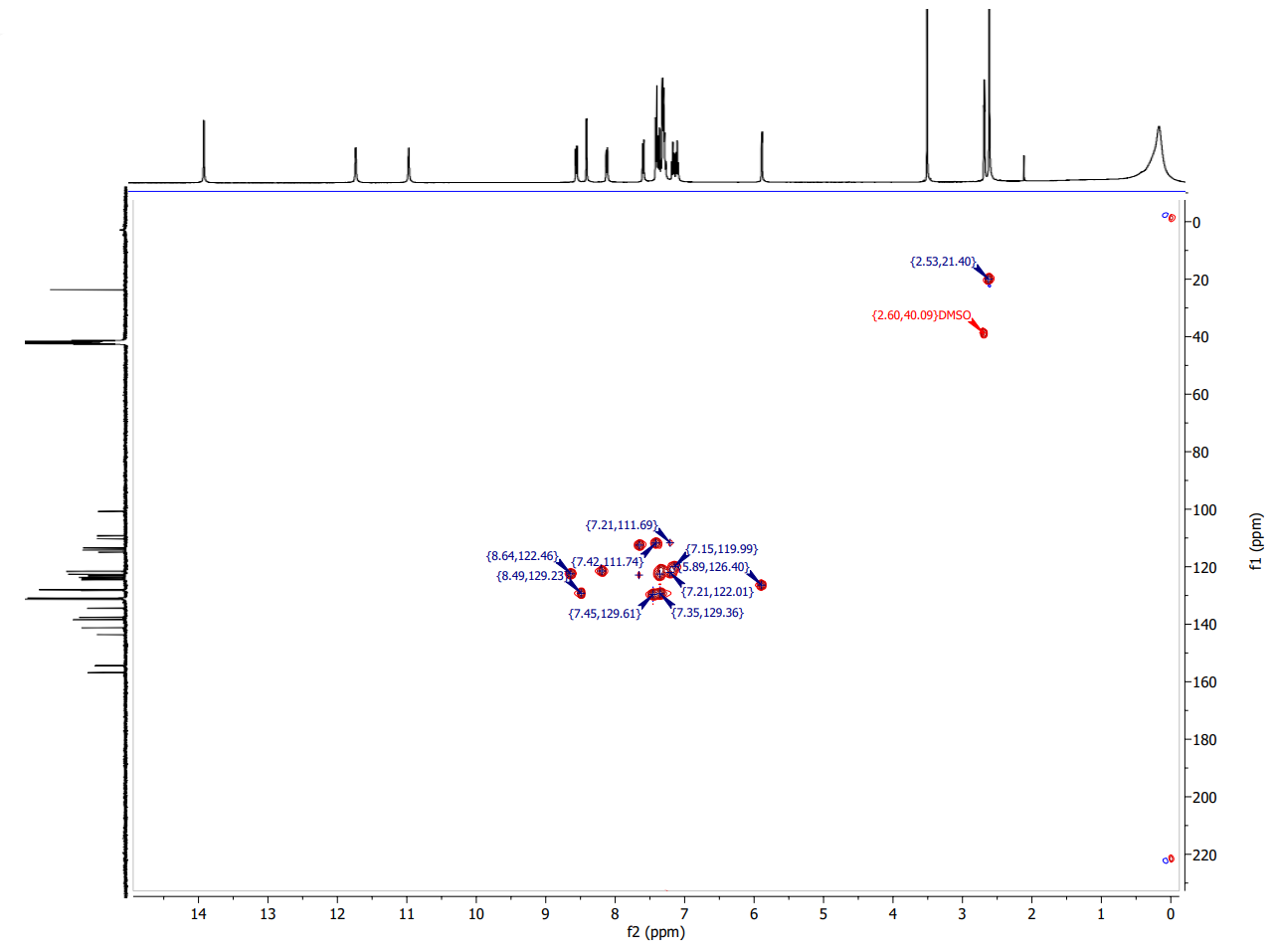


# ^1^H-^13^C NMR (HSQC) spectrum of 3,6-di(1H-indol-3-yl)-4-(p-tolyl)-1H-pyrazolo[3,4-b]pyridine-5-carbonitrile (C1).


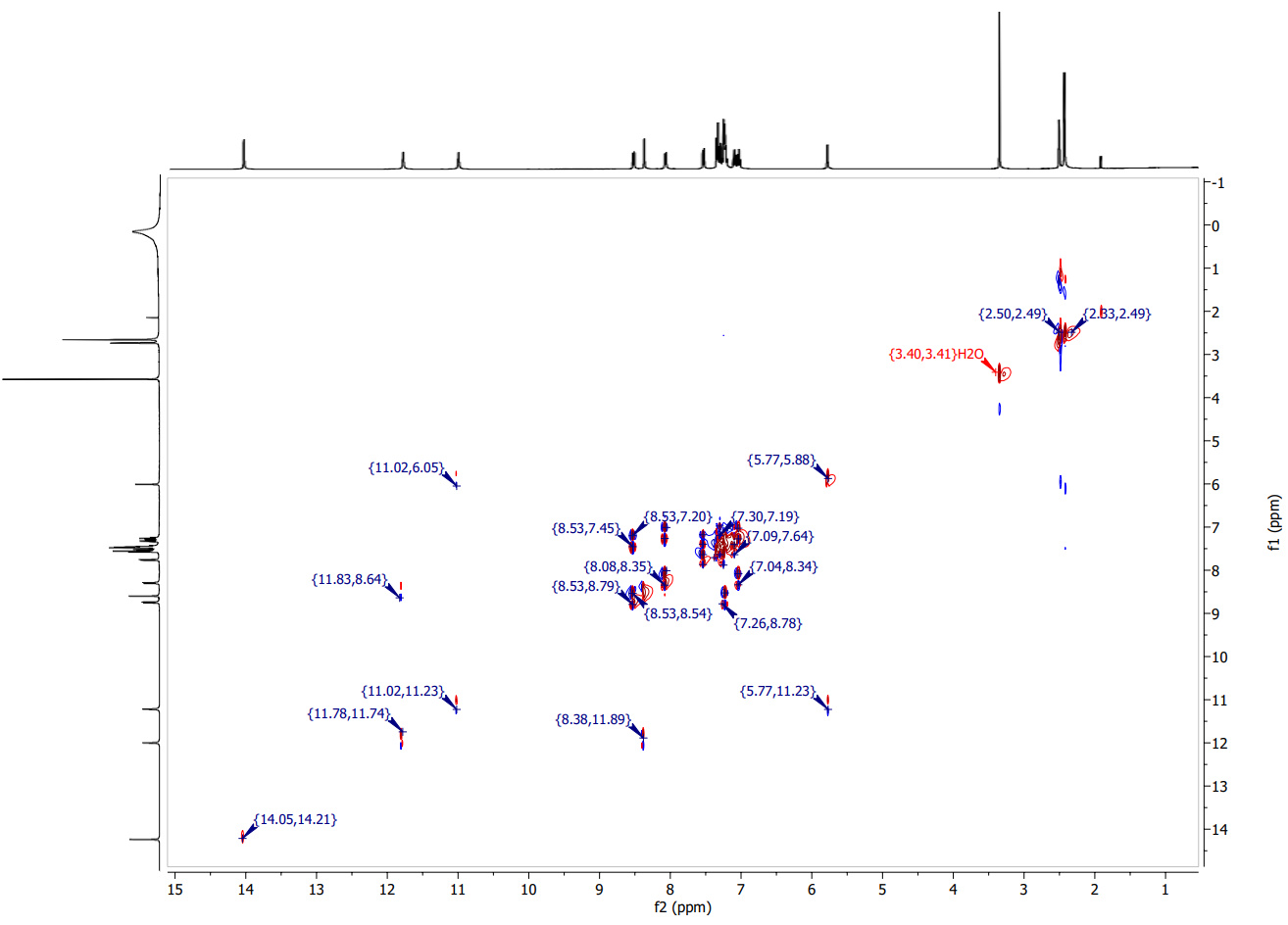


# ^1^H-^1^H NMR (COSY) spectrum of 3,6-di(1H-indol-3-yl)-4-(p-tolyl)-1H-pyrazolo[3,4-b]pyridine-5-carbonitrile (C1).

***
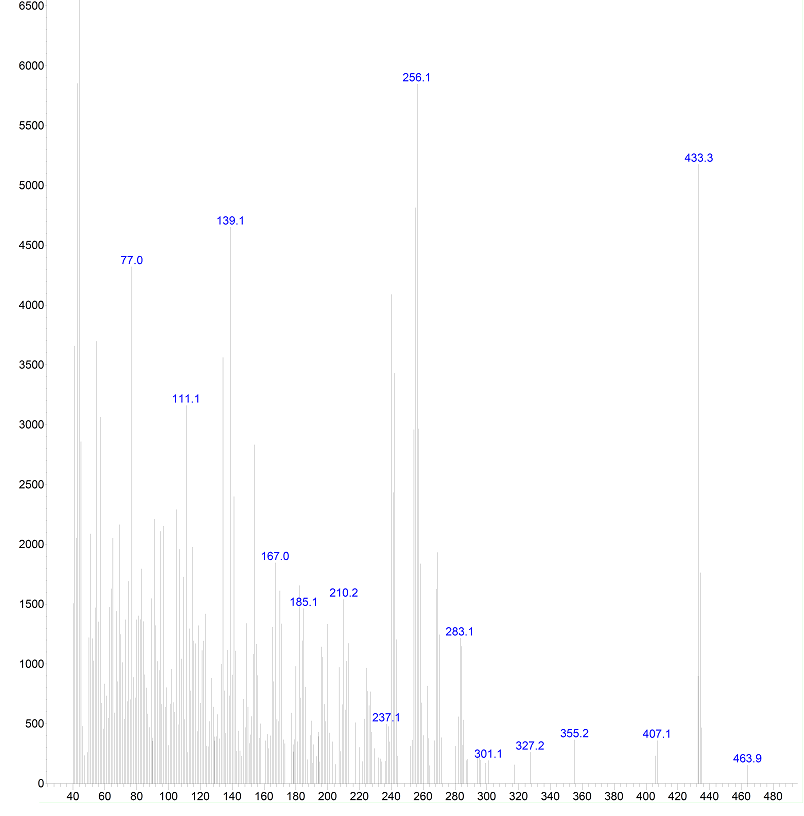
***

# Mass spectrum of 3,6-di(1H-indol-3-yl)-4-(p-tolyl)-1H-pyrazolo[3,4-b]pyridine-5-carbonitrile (C1).

***
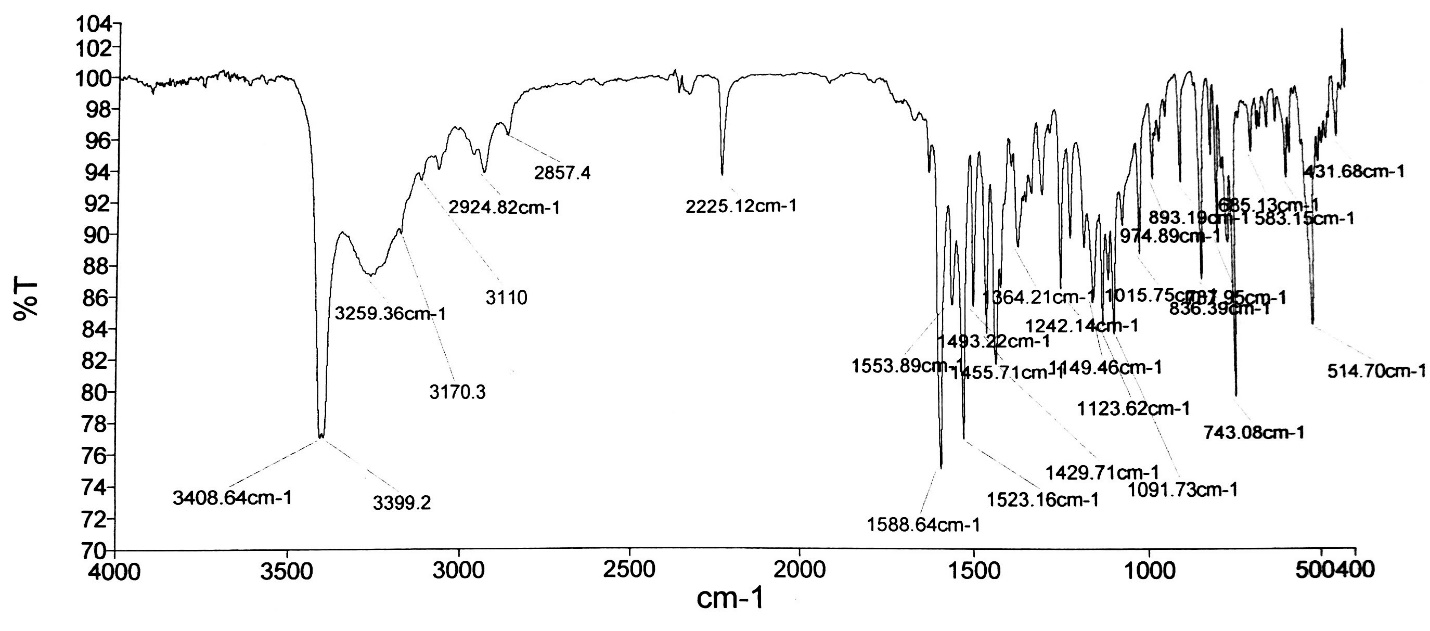
***

# FT-IR spectrum of 4-(4-chlorophenyl)-3,6-di(1H-indol-3-yl)-1H-pyrazolo[3,4-b]pyridine-5-carbonitrile (C2).

***
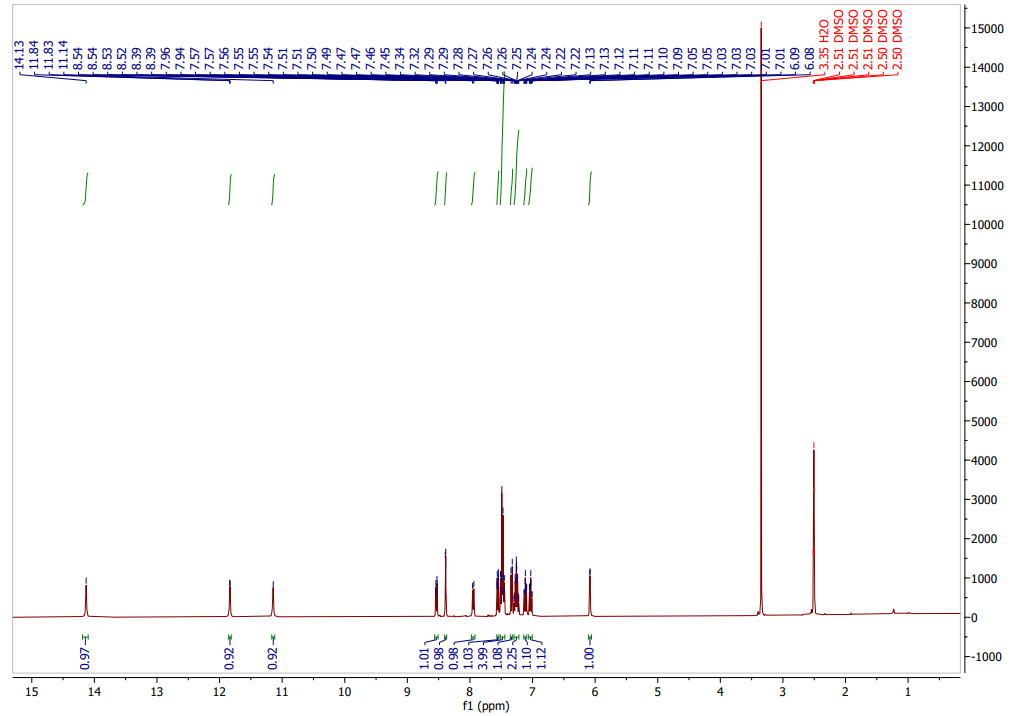
***

# ^1^H-NMR spectrum of 4-(4-chlorophenyl)-3,6-di(1H-indol-3-yl)-1H-pyrazolo[3,4-b]pyridine-5-carbonitrile (C2).

***
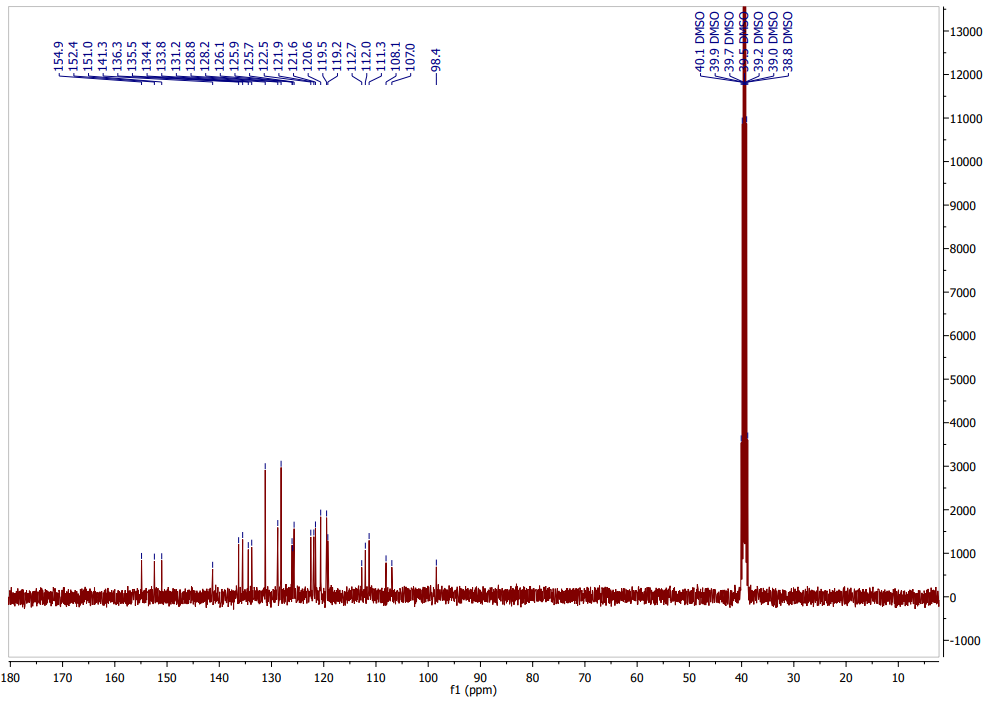
***

# ^13^C-NMR spectrum of 4-(4-chlorophenyl)-3,6-di(1H-indol-3-yl)-1H-pyrazolo[3,4-b]pyridine-5-carbonitrile (C2).

***
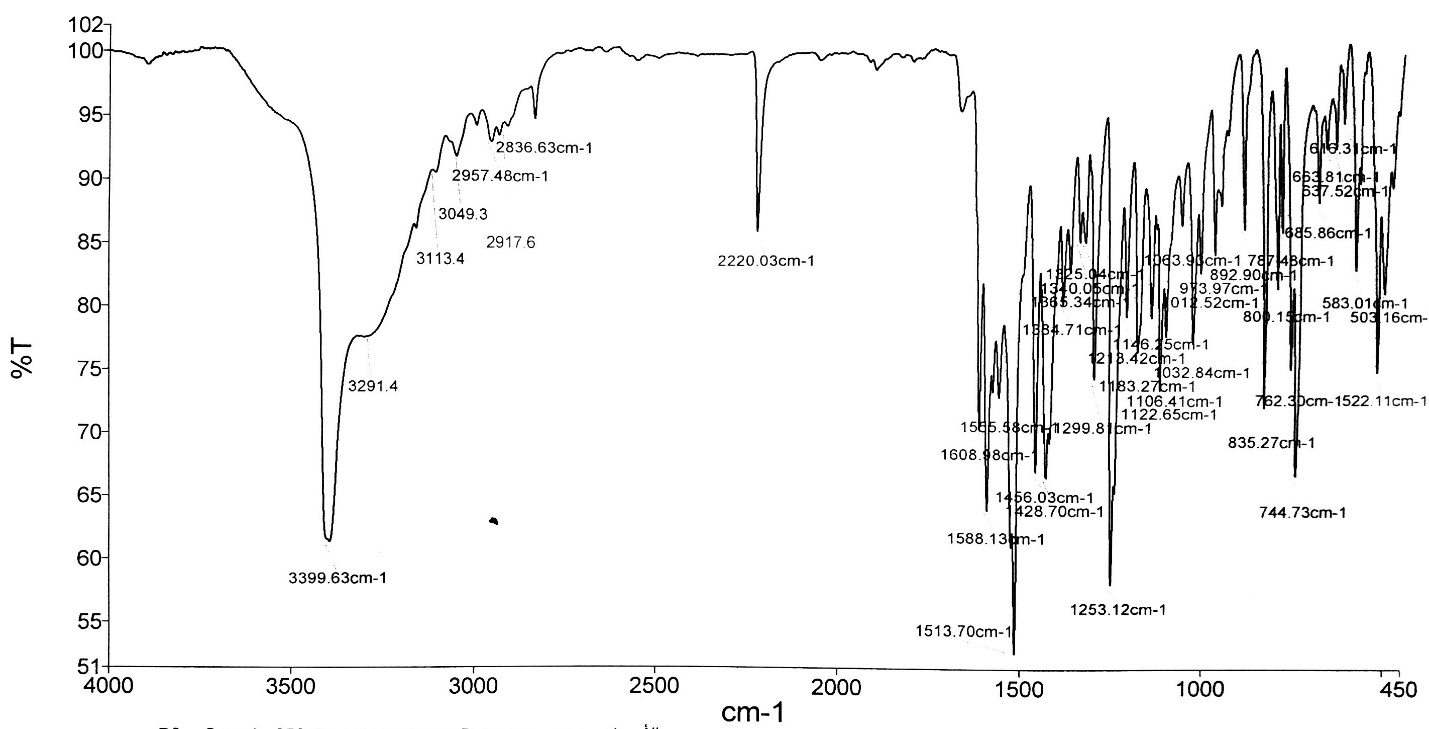
***

# FT-IR spectrum of 3,6-di(1H-indol-3-yl)-4-(4-methoxyphenyl)-1H-pyrazolo[3,4-b]pyridine-5-carbonitrile (C3).

***
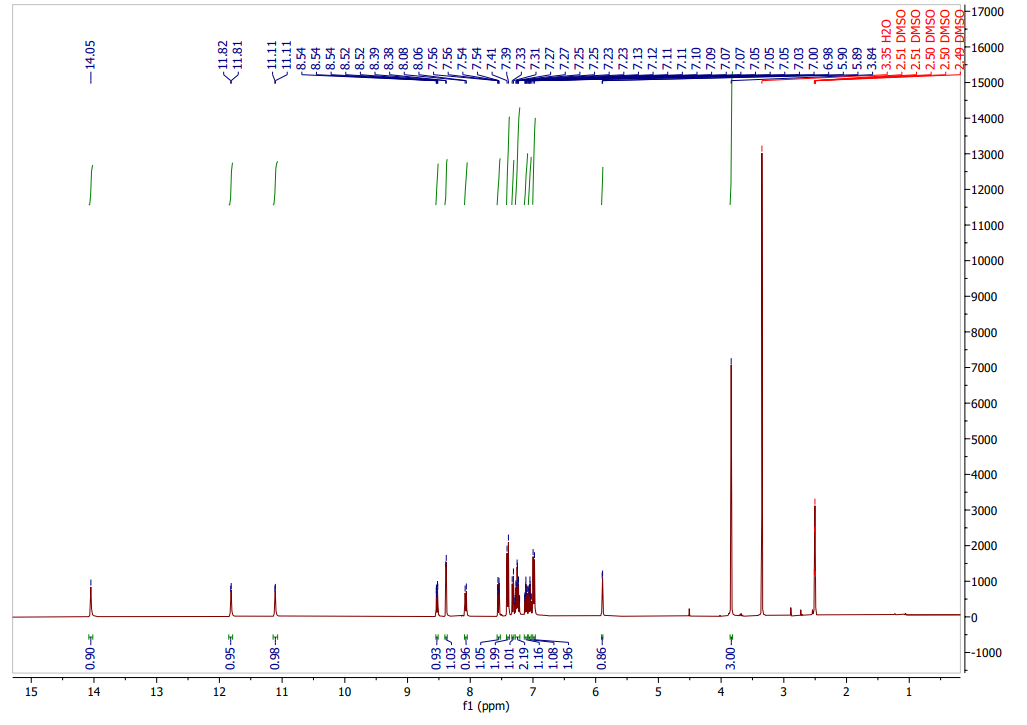
***

# ^1^H-NMR spectrum of 3,6-di(1H-indol-3-yl)-4-(4-methoxyphenyl)-1H-pyrazolo[3,4-b]pyridine-5-carbonitrile (C3).

***
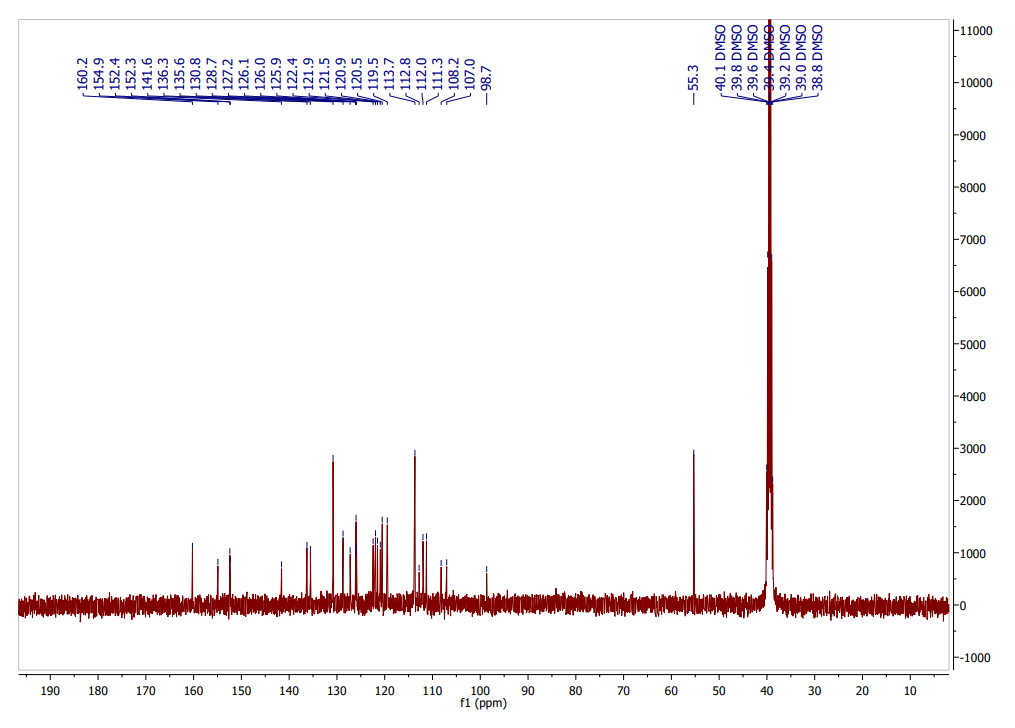
***

# ^13^C-NMR spectrum of 3,6-di(1H-indol-3-yl)-4-(4-methoxyphenyl)-1H-pyrazolo[3,4-b]pyridine-5-carbonitrile (C3).

***
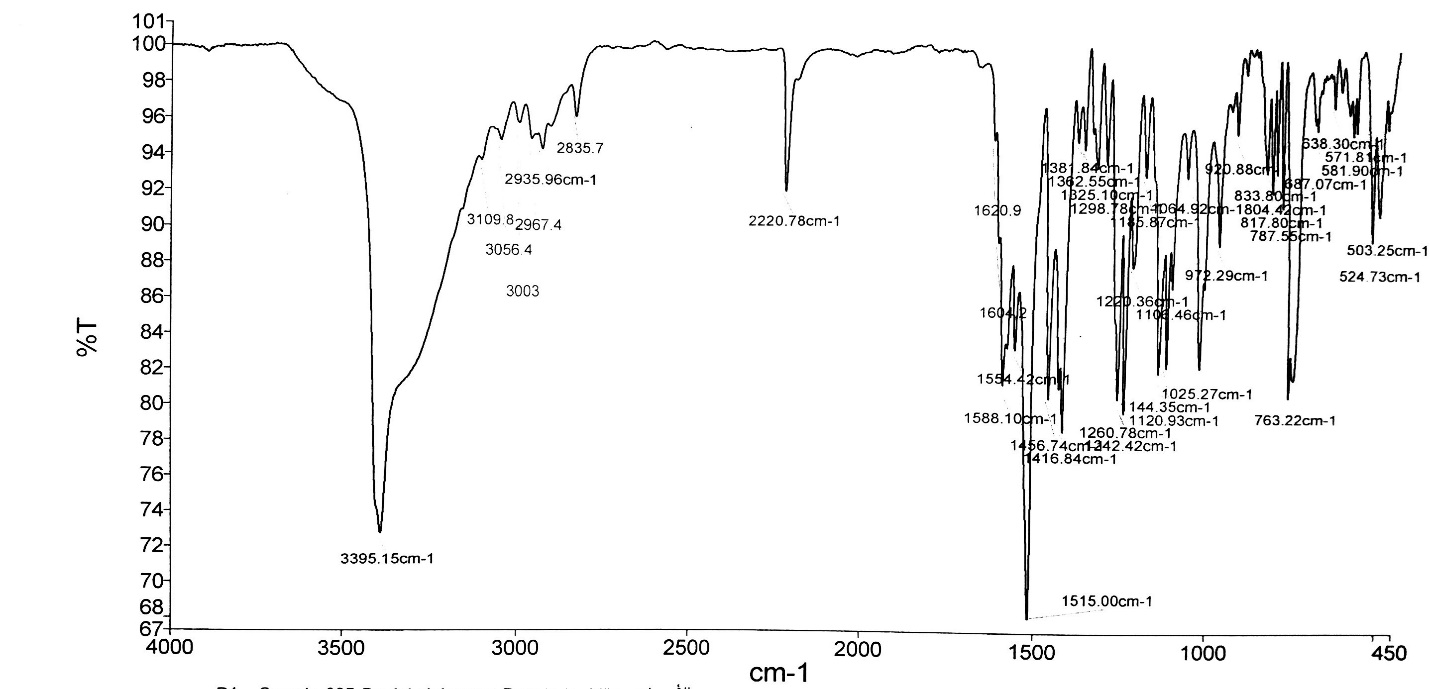
***

# FT-IR spectrum of 4-(3,4-dimethoxyphenyl)-3,6-di(1H-indol-3-yl)-1H-pyrazolo[3,4-b]pyridine-5-carbonitrile (C4).

***
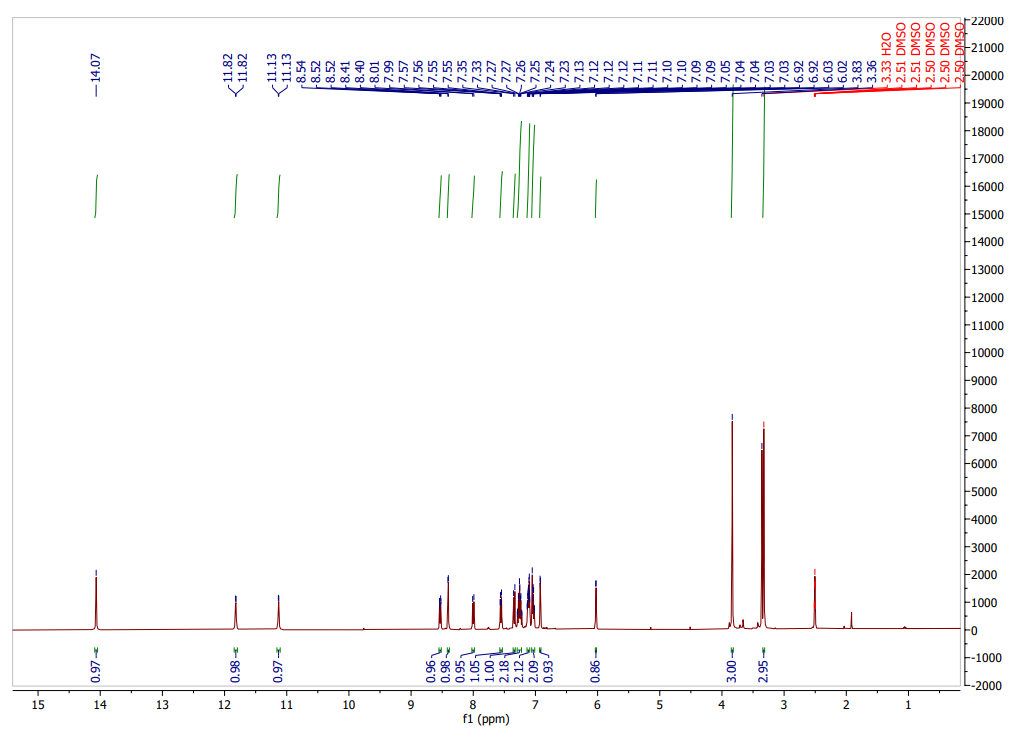
***

# ^1^H-NMR spectrum of 4-(3,4-dimethoxyphenyl)-3,6-di(1H-indol-3-yl)-1H-pyrazolo[3,4-b]pyridine-5-carbonitrile (C4).

***
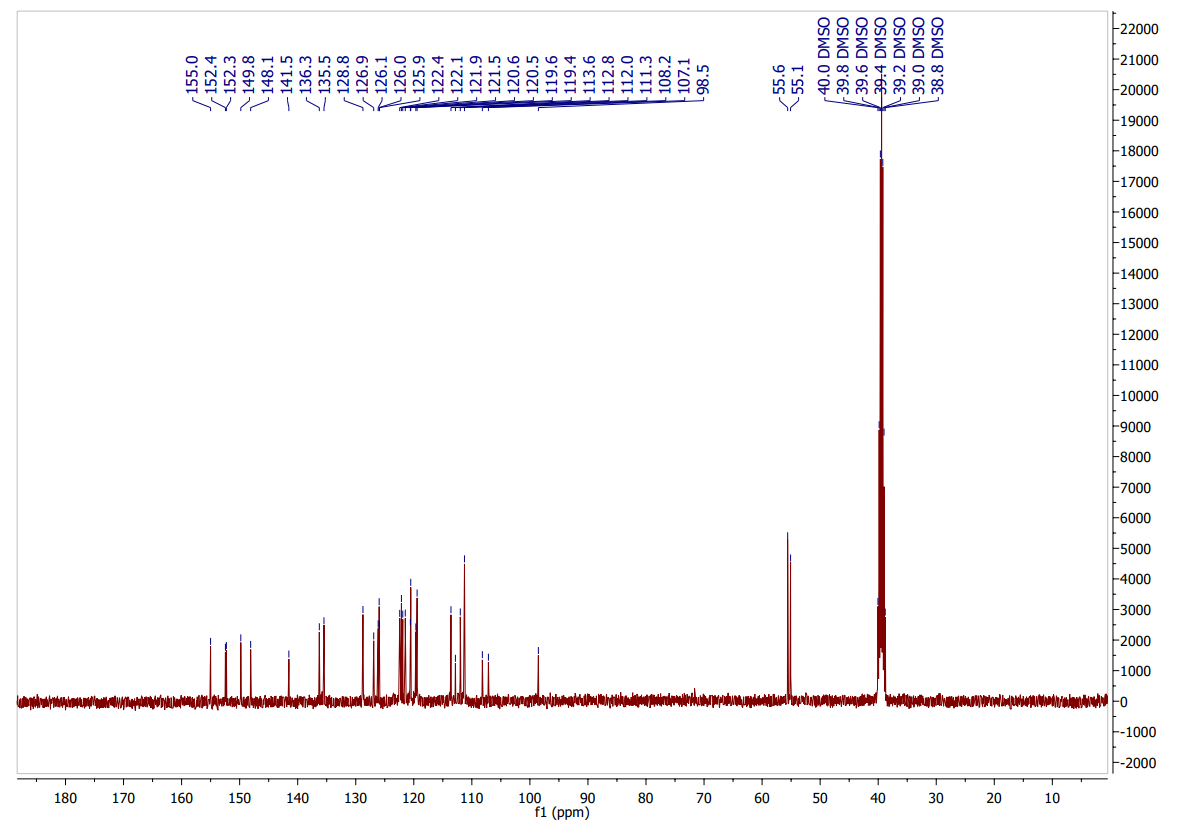
***

# ^13^C-NMR spectrum of 4-(3,4-dimethoxyphenyl)-3,6-di(1H-indol-3-yl)-1H-pyrazolo[3,4-b]pyridine-5-carbonitrile (C4).

***
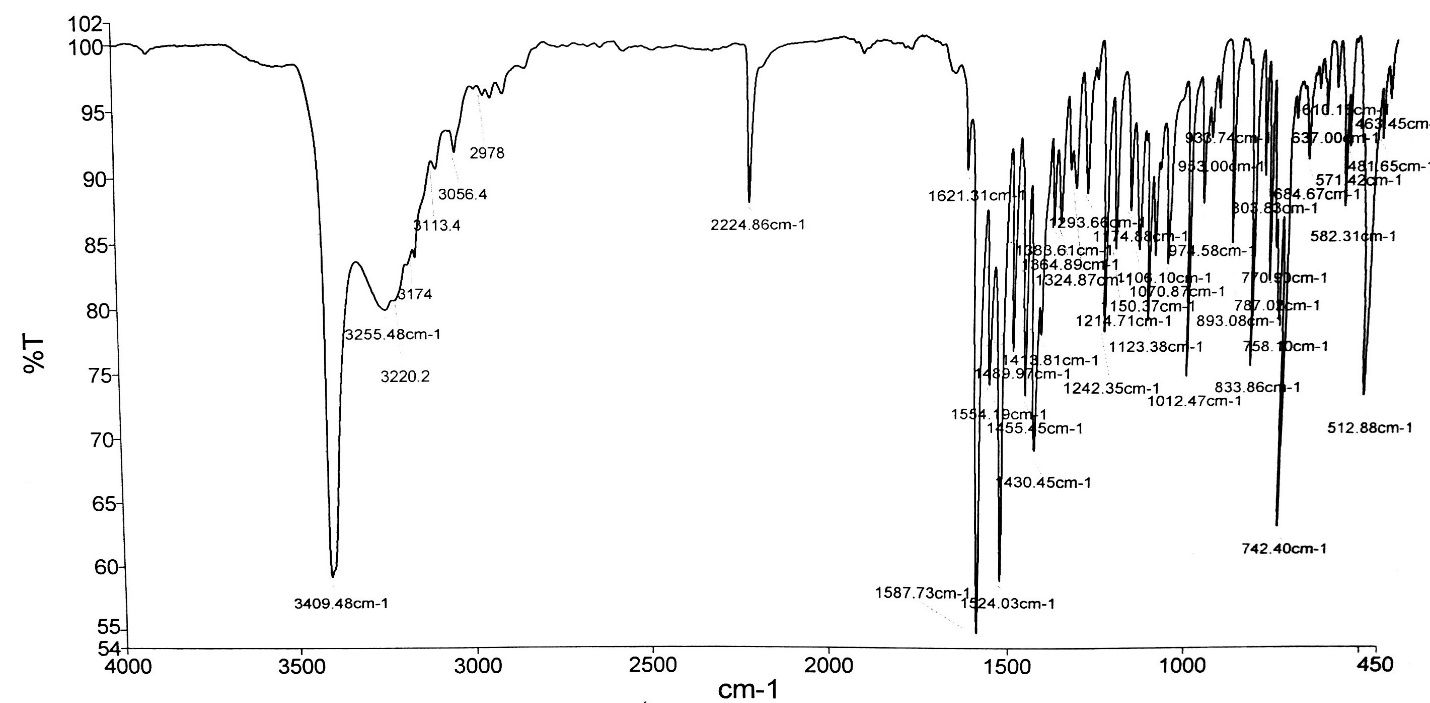
***

# FT-IR spectrum of 4-(4-bromophenyl)-3,6-di(1H-indol-3-yl)-1H-pyrazolo[3,4-b]pyridine-5-carbonitrile (C5).

***
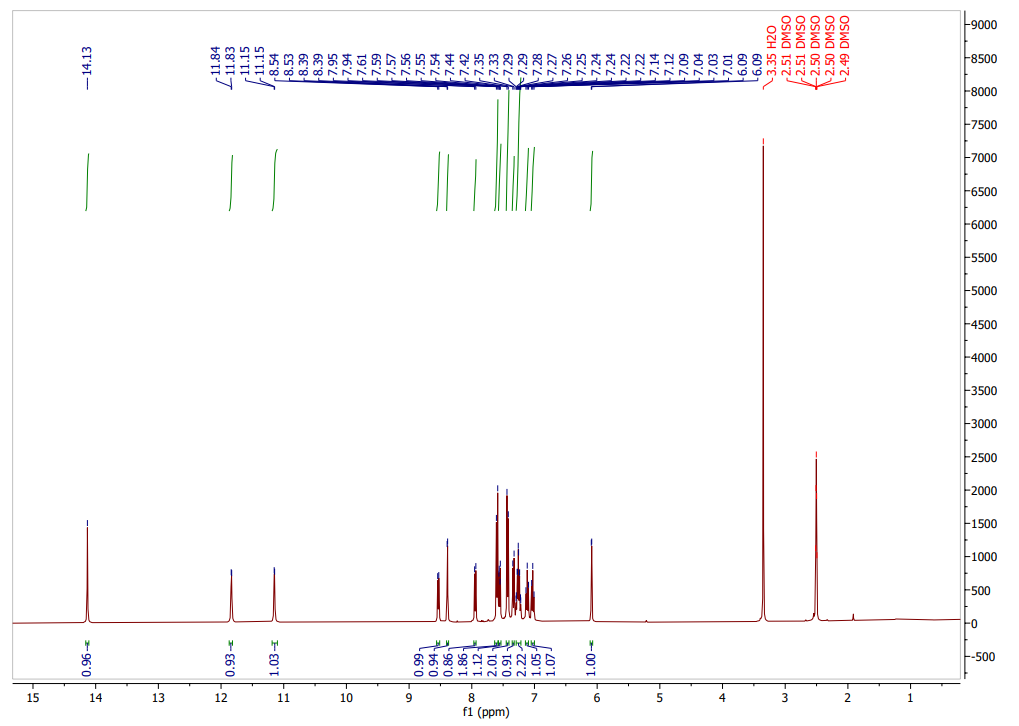
***

# ^1^H-NMR spectrum of 4-(4-bromophenyl)-3,6-di(1H-indol-3-yl)-1H-pyrazolo[3,4-b]pyridine-5-carbonitrile (C5).

***
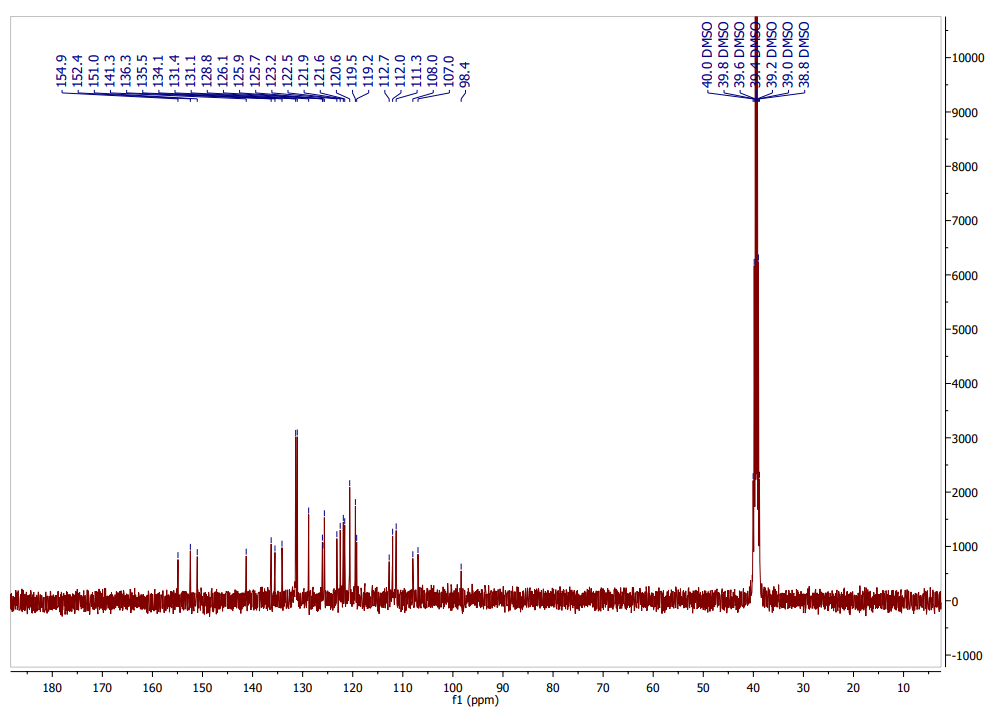
***

# ^13^C-NMR spectrum of 4-(4-bromophenyl)-3,6-di(1H-indol-3-yl)-1H-pyrazolo[3,4-b]pyridine-5-carbonitrile (C5).

***
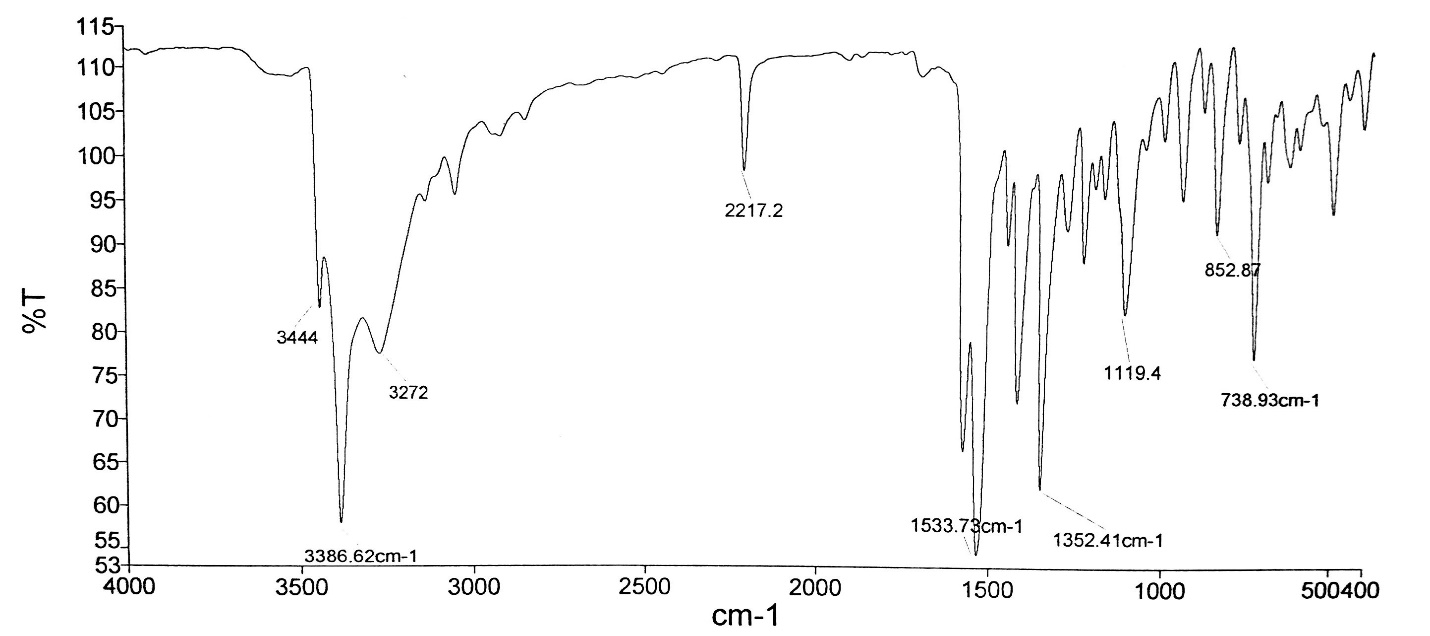
***

# FT-IR spectrum of 3,6-di(1H-indol-3-yl)-4-(4-nitrophenyl)-1H-pyrazolo[3,4-b]pyridine-5-carbonitrile (C6).

***
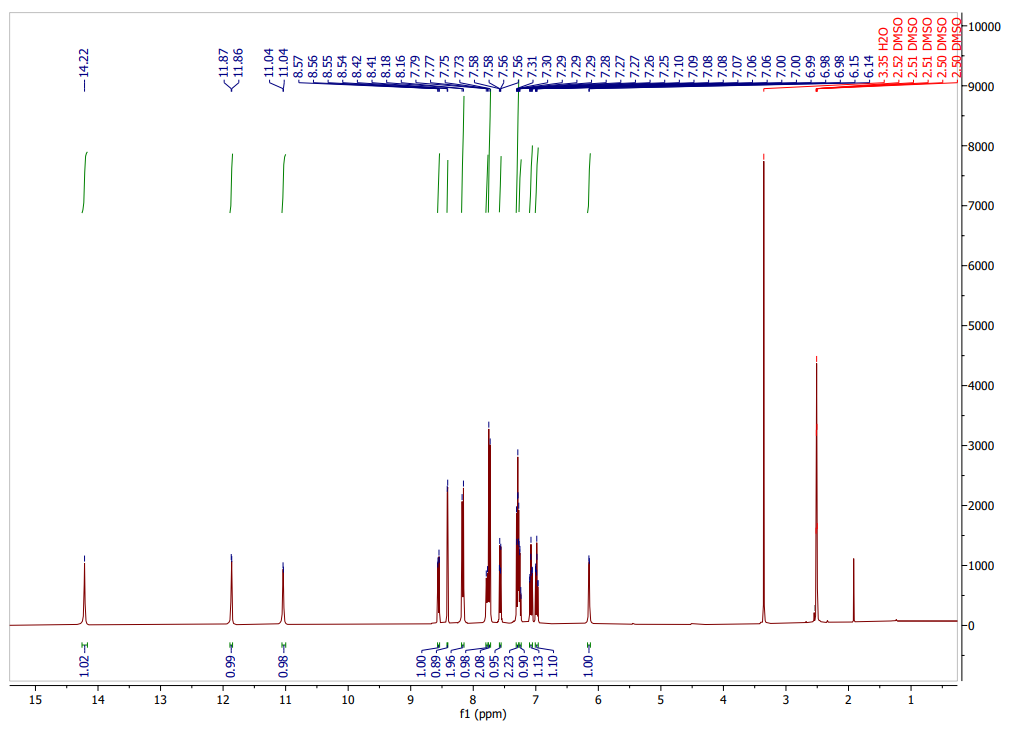
***

# ^1^H-NMR spectrum of 3,6-di(1H-indol-3-yl)-4-(4-nitrophenyl)-1H-pyrazolo[3,4-b]pyridine-5-carbonitrile (C6).

***
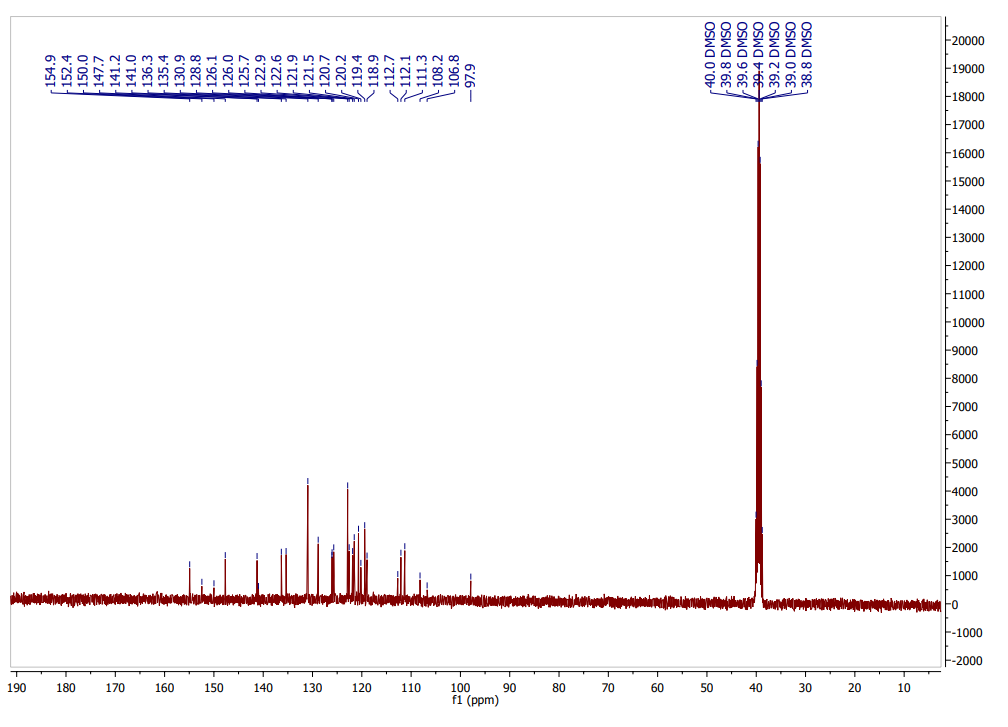
***

# ^13^C-NMR spectrum of 3,6-di(1H-indol-3-yl)-4-(4-nitrophenyl)-1H-pyrazolo[3,4-b]pyridine-5-carbonitrile (C6).

***
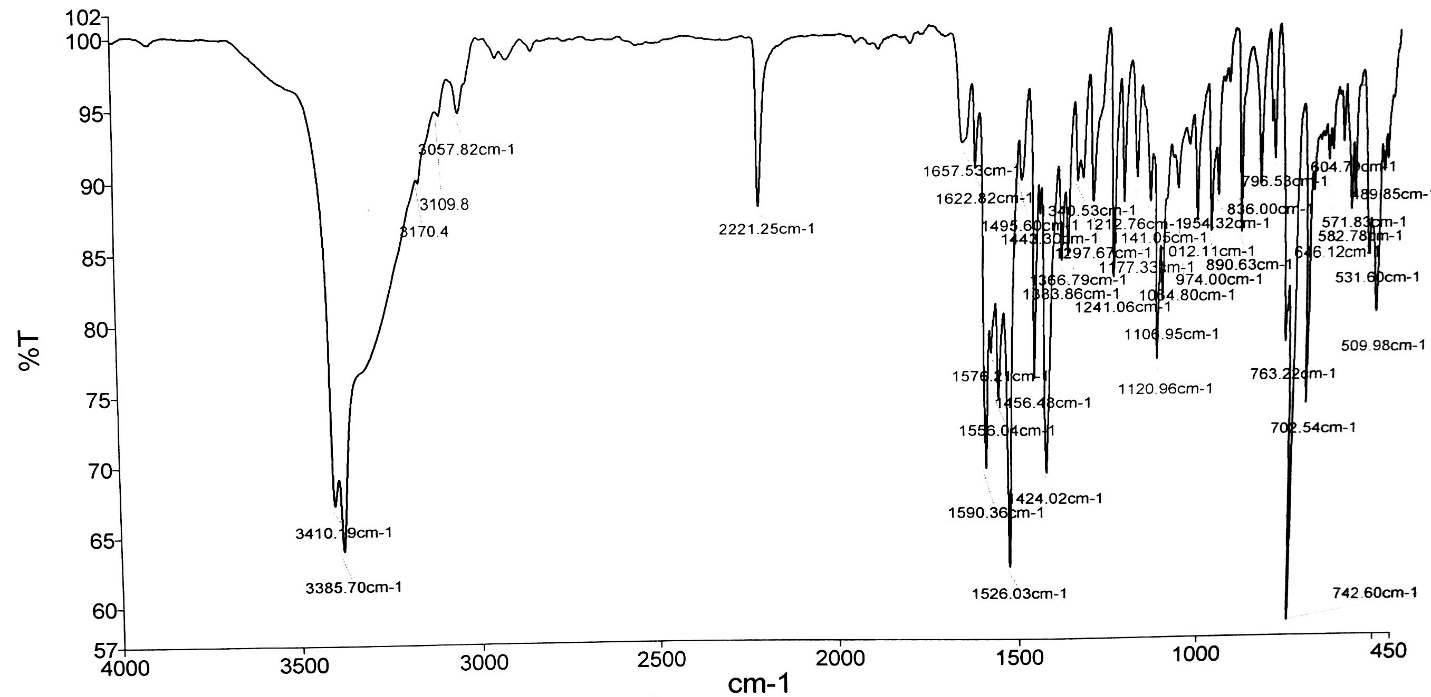
***

# FT-IR spectrum of 3,6-di(1H-indol-3-yl)-4-phenyl-1H-pyrazolo[3,4-b]pyridine-5-carbonitrile (C7).

***
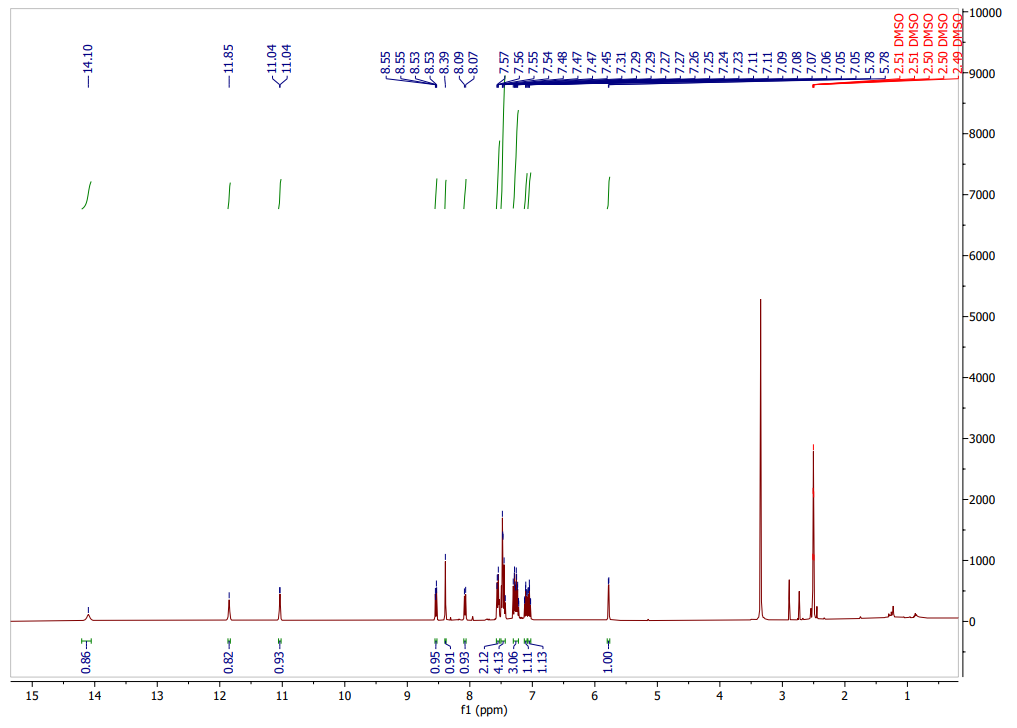
***

# ^1^H-NMR spectrum of 3,6-di(1H-indol-3-yl)-4-phenyl-1H-pyrazolo[3,4-b]pyridine-5-carbonitrile (C7).

***
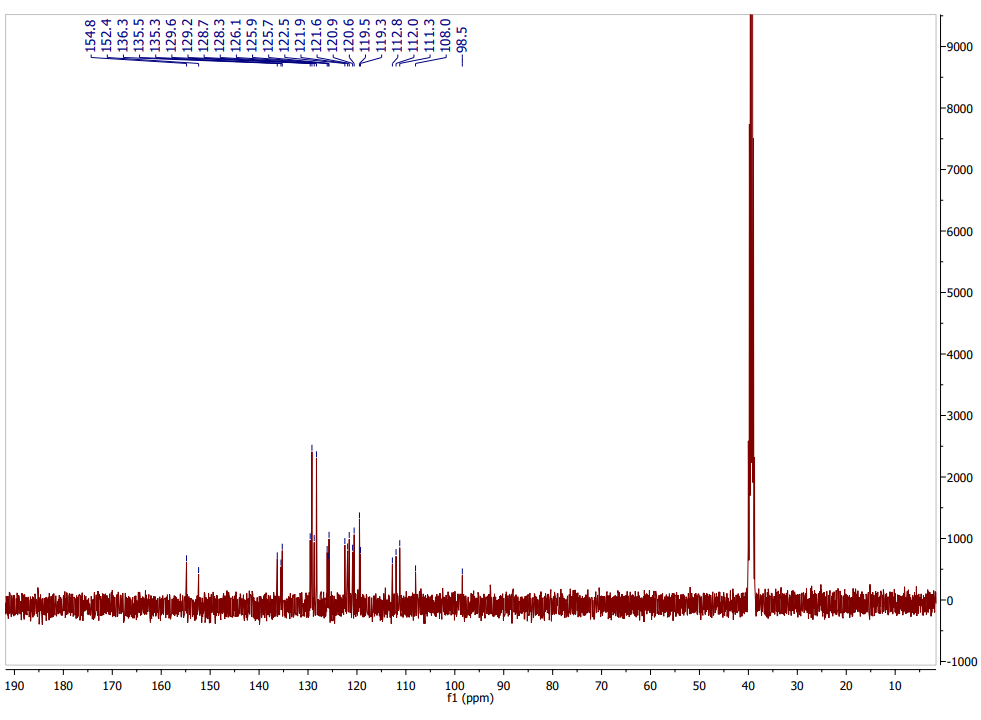
***

# ^13^C-NMR spectrum of 3,6-di(1H-indol-3-yl)-4-phenyl-1H-pyrazolo[3,4-b]pyridine-5-carbonitrile (C7).

***
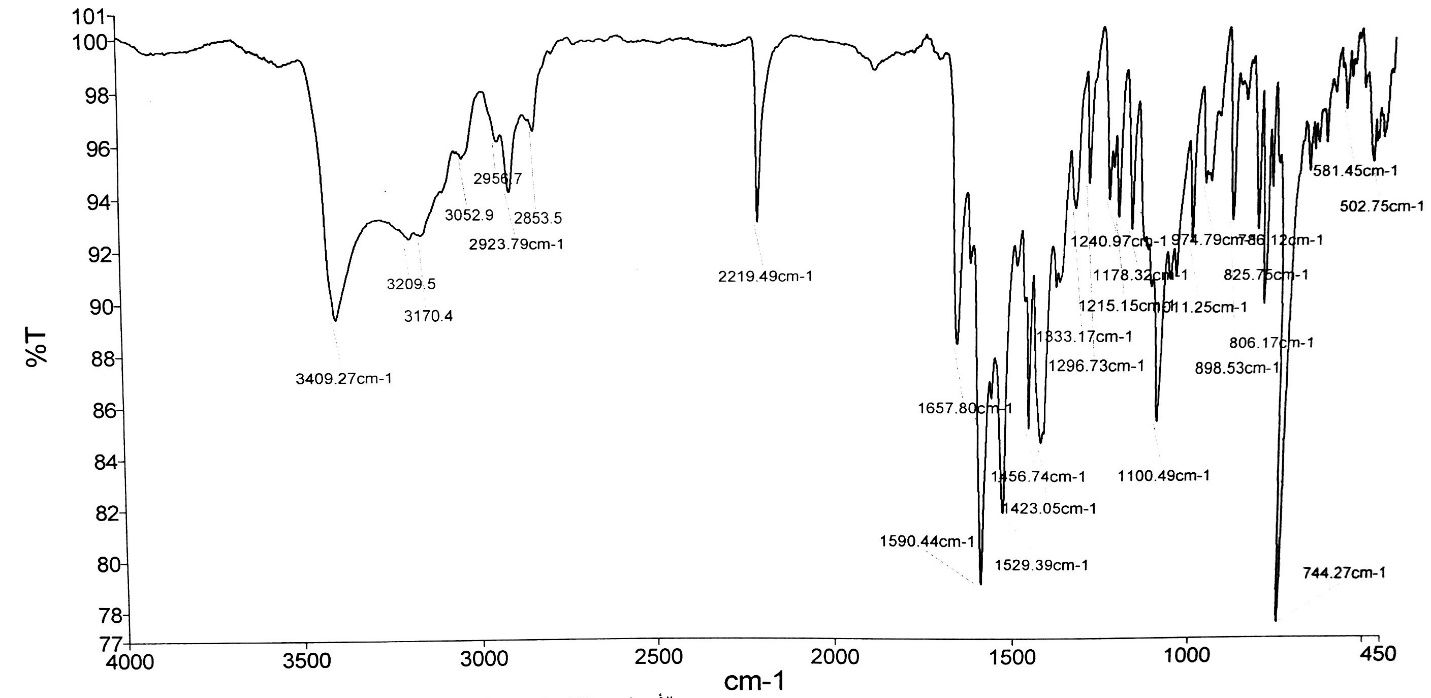
***

# FT-IR spectrum of 4-(4-hydroxyphenyl)-3,6-di(1H-indol-3-yl)-1H-pyrazolo[3,4-b]pyridine-5-carbonitrile (C8).

***
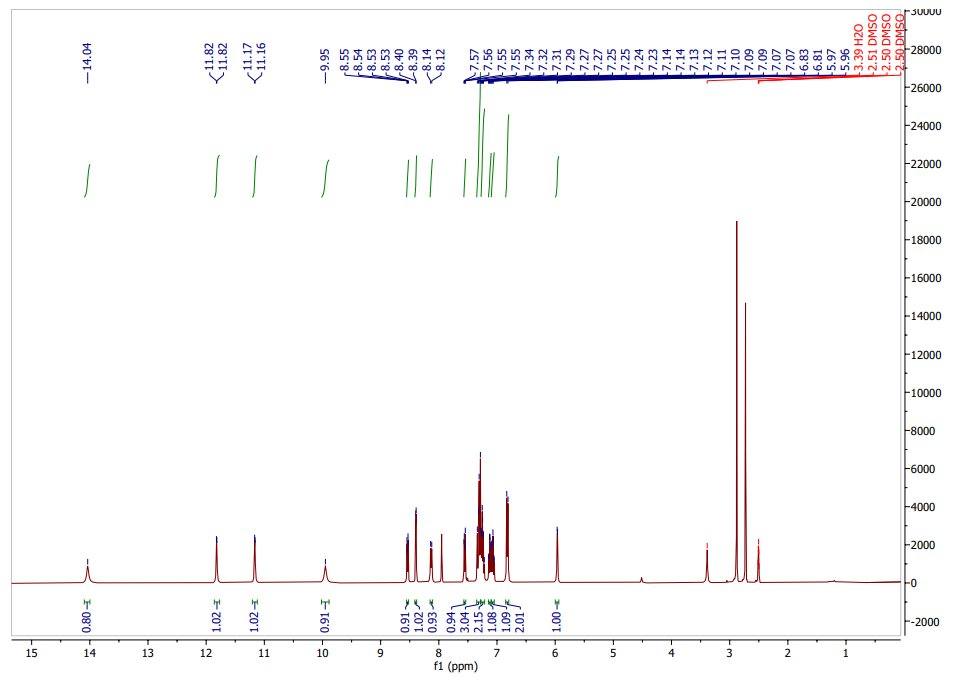
***

# ^1^H-NMR spectrum of 4-(4-hydroxyphenyl)-3,6-di(1H-indol-3-yl)-1H-pyrazolo[3,4-b]pyridine-5-carbonitrile (C8).

***
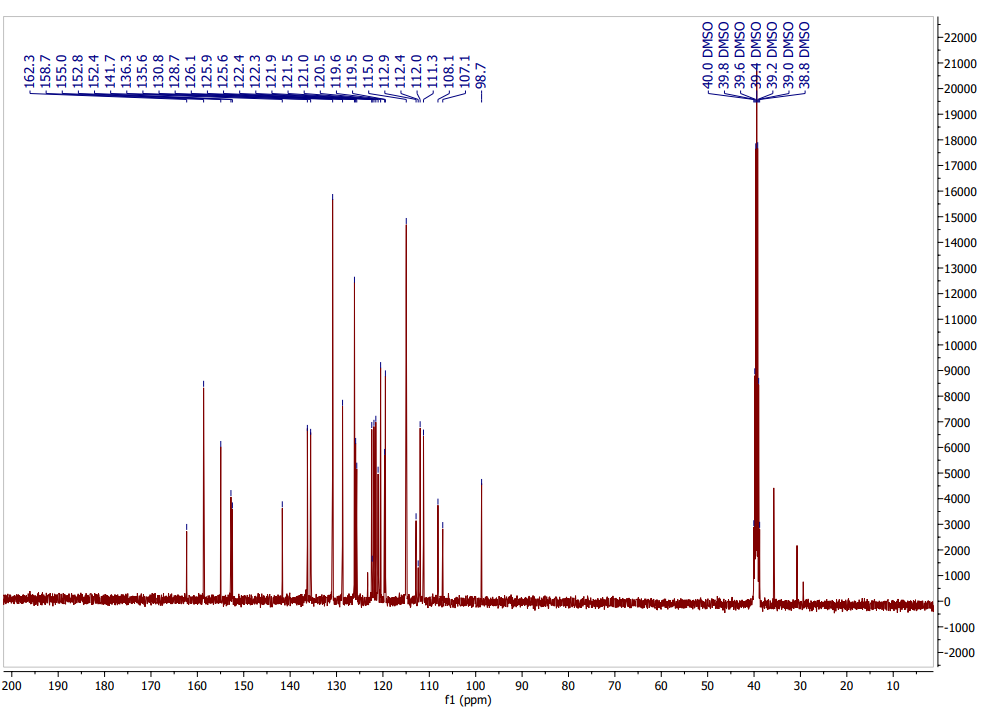
***

# ^13^C-NMR spectrum of 4-(4-hydroxyphenyl)-3,6-di(1H-indol-3-yl)-1H-pyrazolo[3,4-b]pyridine-5-carbonitrile (C8).

***
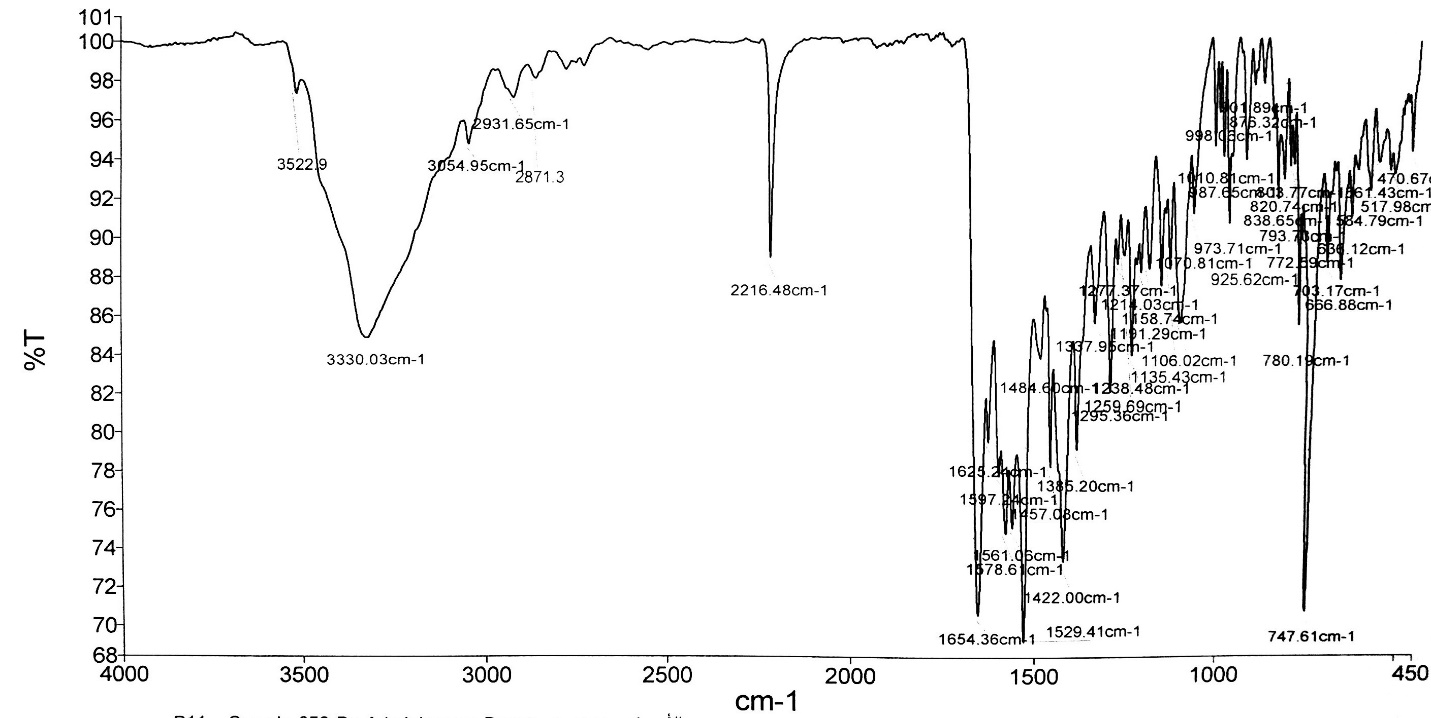
***

# FT-IR spectrum of 4-(3-hydroxyphenyl)-3,6-di(1H-indol-3-yl)-1H-pyrazolo[3,4-b]pyridine-5-carbonitrile (C9).

***
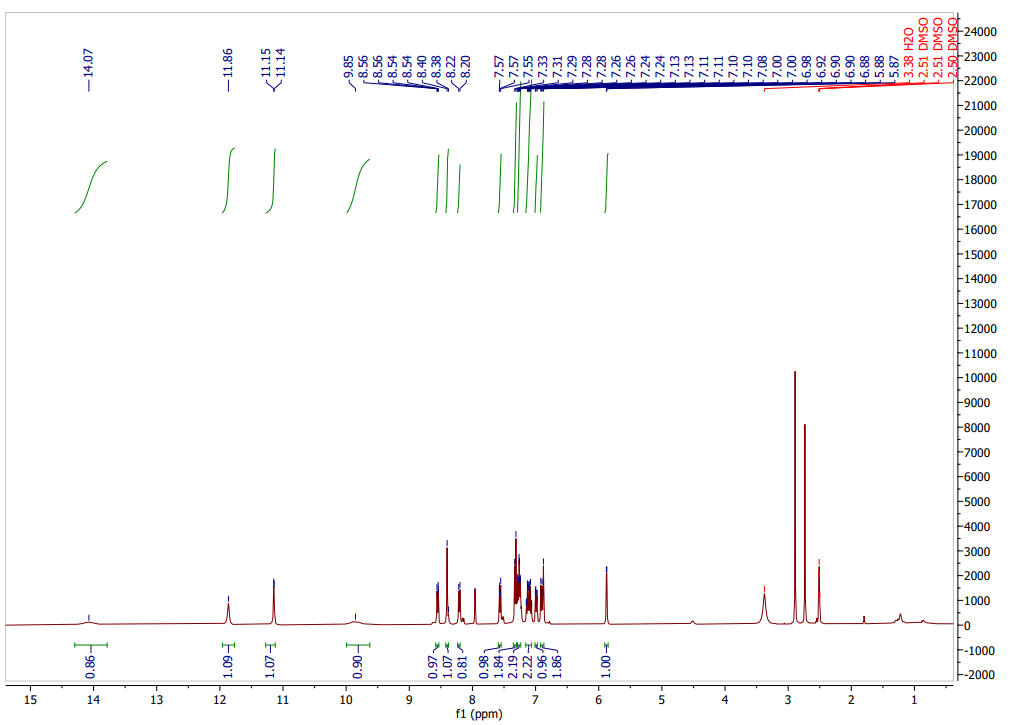
***

# ^1^H-NMR spectrum of 4-(3-hydroxyphenyl)-3,6-di(1H-indol-3-yl)-1H-pyrazolo[3,4-b]pyridine-5-carbonitrile (C9).

***
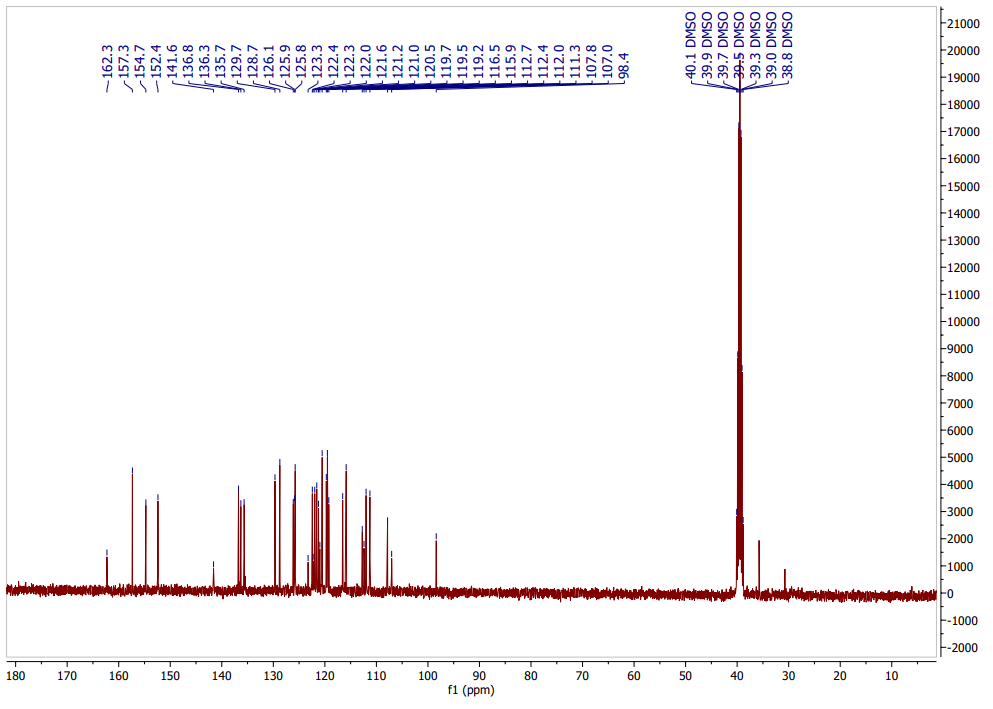
***

# ^13^C-NMR spectrum of 4-(3-hydroxyphenyl)-3,6-di(1H-indol-3-yl)-1H-pyrazolo[3,4-b]pyridine-5-carbonitrile (C9).

***
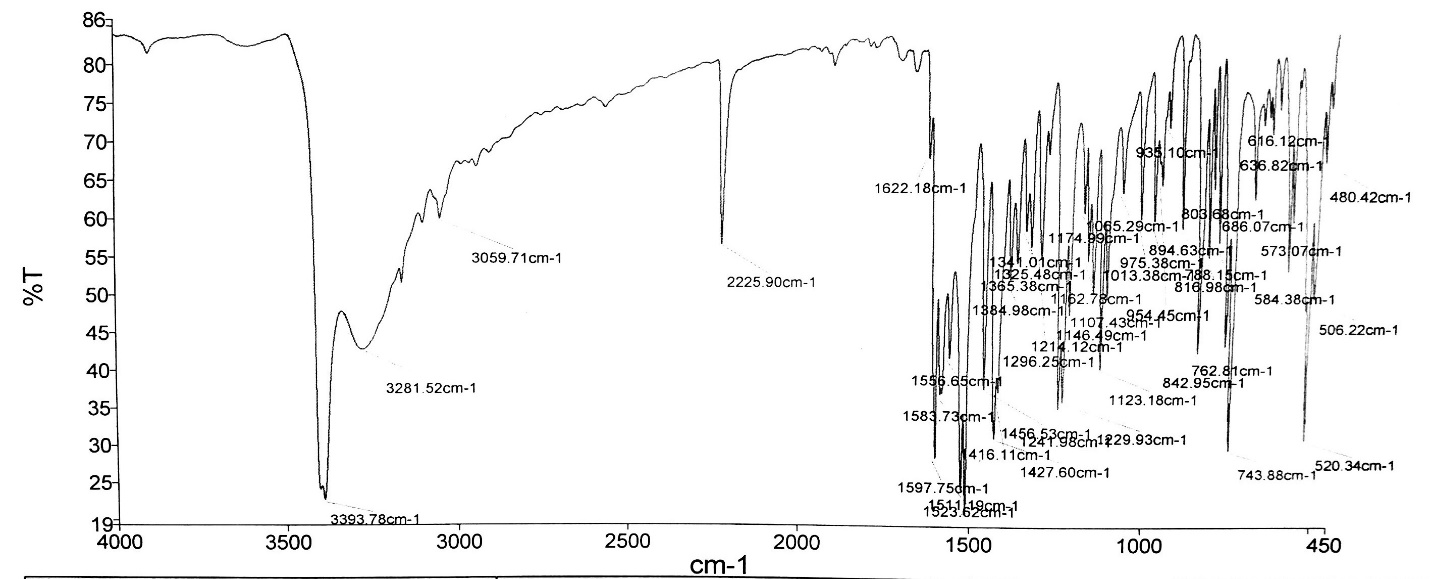
***

# FT-IR spectrum of 4-(4-fluorophenyl)-3,6-di(1H-indol-3-yl)-1H-pyrazolo[3,4-b]pyridine-5-carbonitrile (C10).

***
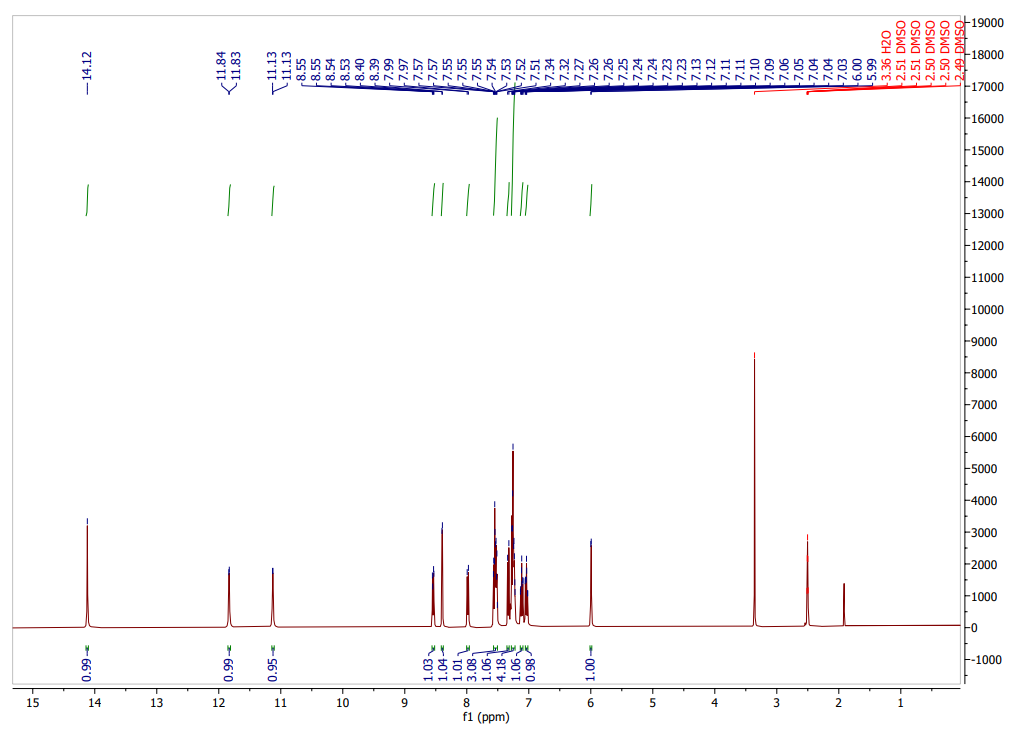
***

# ^1^H-NMR spectrum of 4-(4-fluorophenyl)-3,6-di(1H-indol-3-yl)-1H-pyrazolo[3,4-b]pyridine-5-carbonitrile (C10).

***
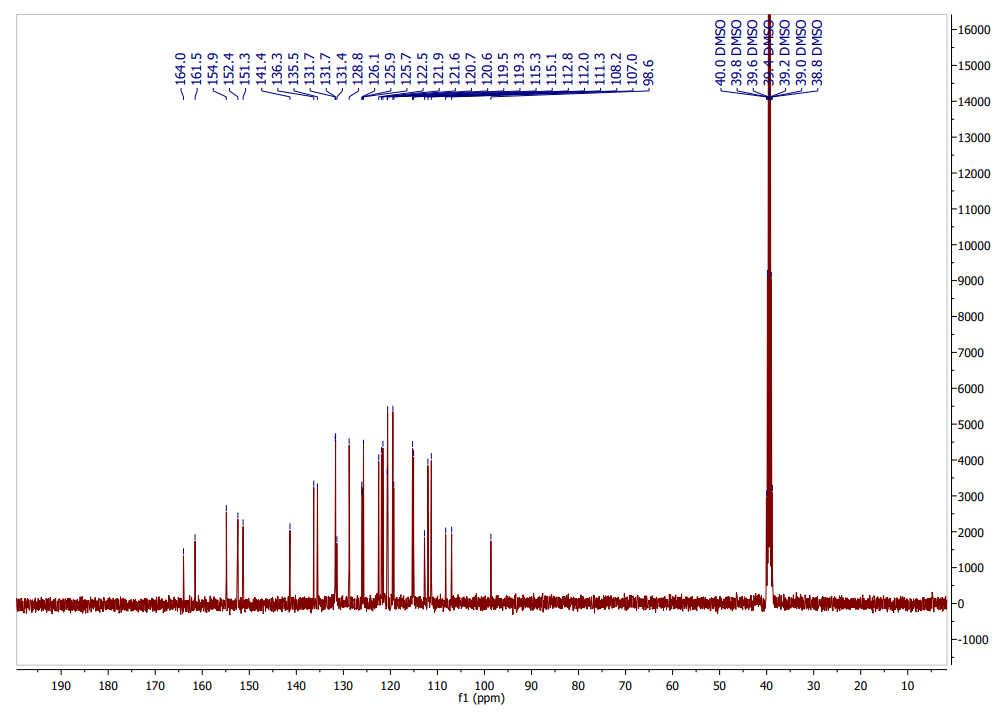
***

# ^13^C-NMR spectrum of 4-(4-fluorophenyl)-3,6-di(1H-indol-3-yl)-1H-pyrazolo[3,4-b]pyridine-5-carbonitrile (C10).

***
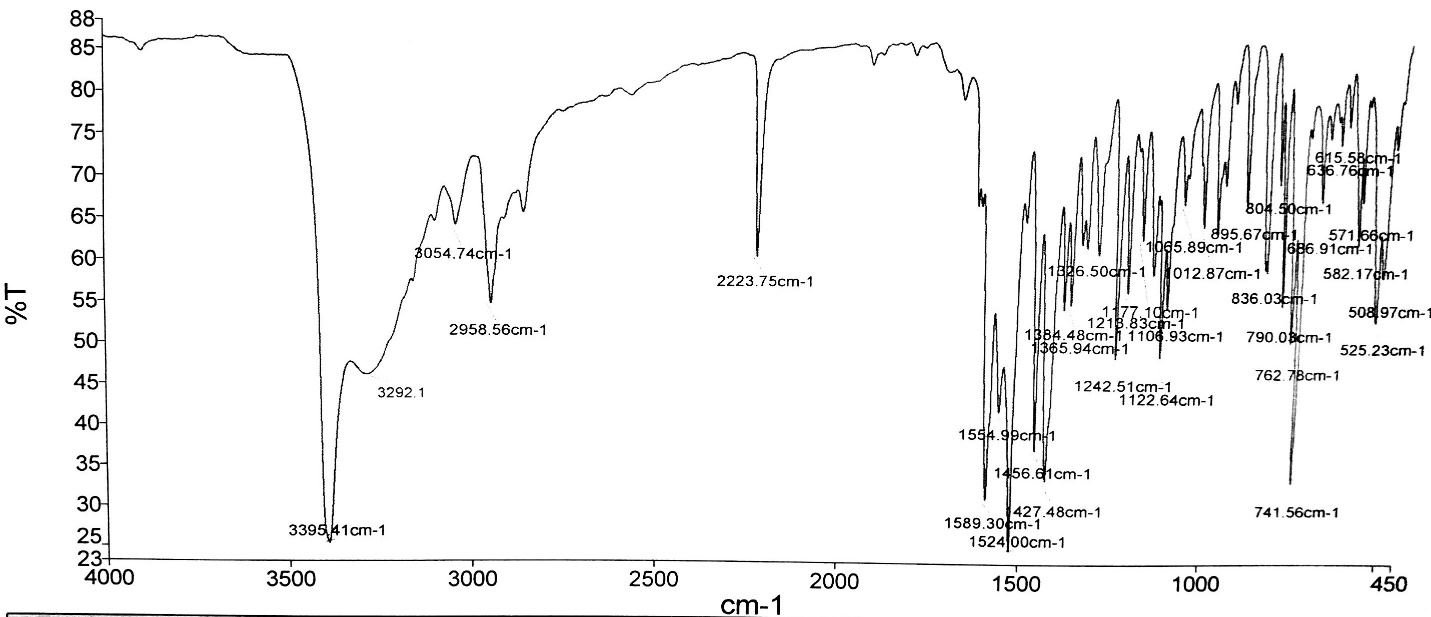
***

# FT-IR spectrum of 3,6-di(1H-indol-3-yl)-4-(4-isopropylphenyl)-1H-pyrazolo[3,4-b]pyridine-5-carbonitrile (C11).

***
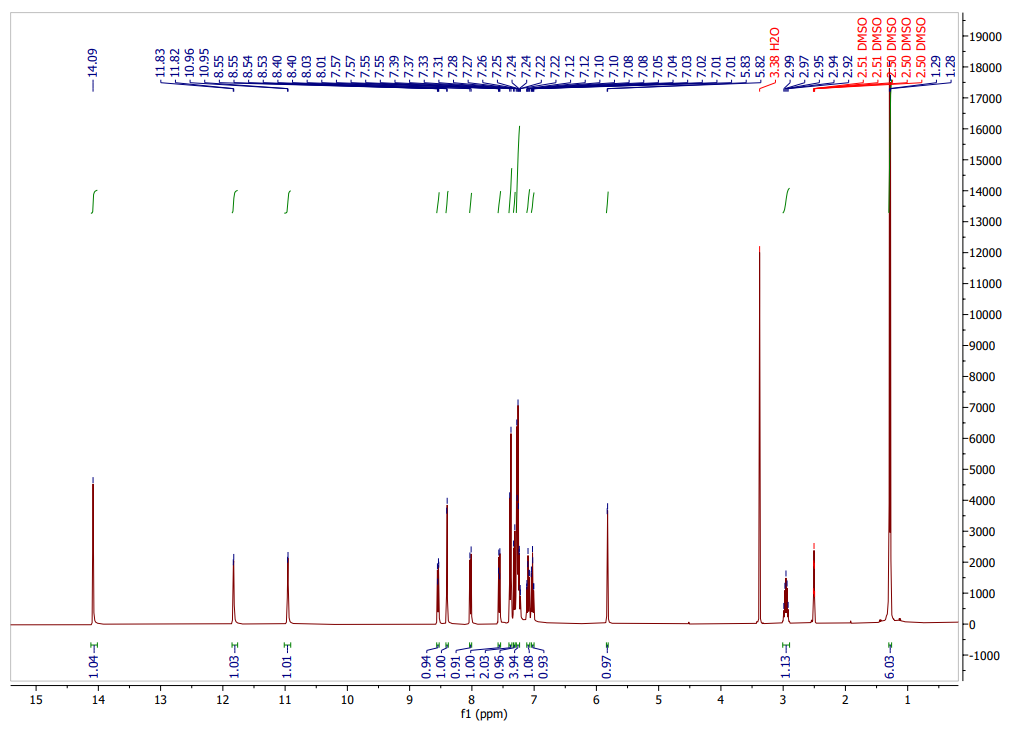
***

# ^1^H-NMR spectrum of 3,6-di(1H-indol-3-yl)-4-(4-isopropylphenyl)-1H-pyrazolo[3,4-b]pyridine-5-carbonitrile (C11).

***
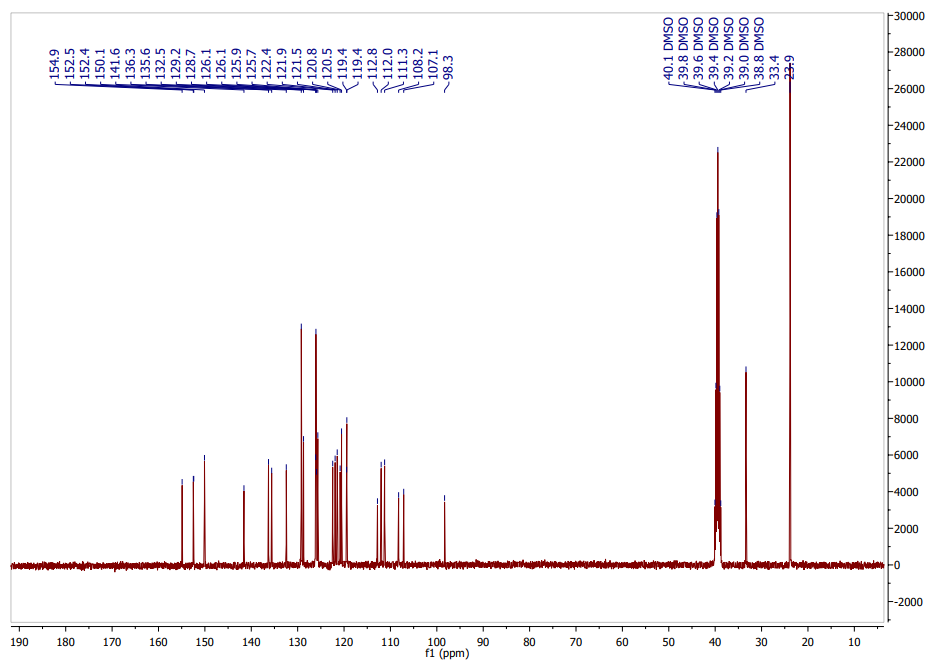
***

# ^13^C-NMR spectrum of 3,6-di(1H-indol-3-yl)-4-(4-isopropylphenyl)-1H-pyrazolo[3,4-b]pyridine-5-carbonitrile (C11).

***
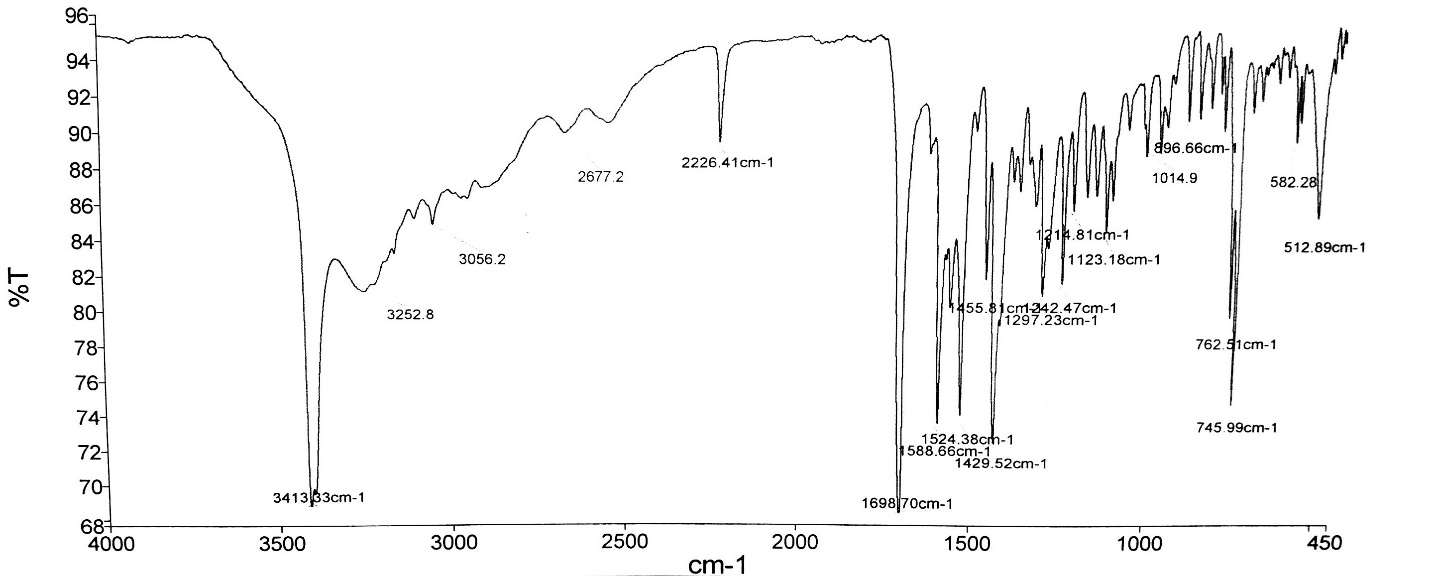
***

# FT-IR spectrum of 4-(5-cyano-3,6-di(1H-indol-3-yl)-1H-pyrazolo[3,4-b]pyridin-4-yl)benzoic acid (C12).

***
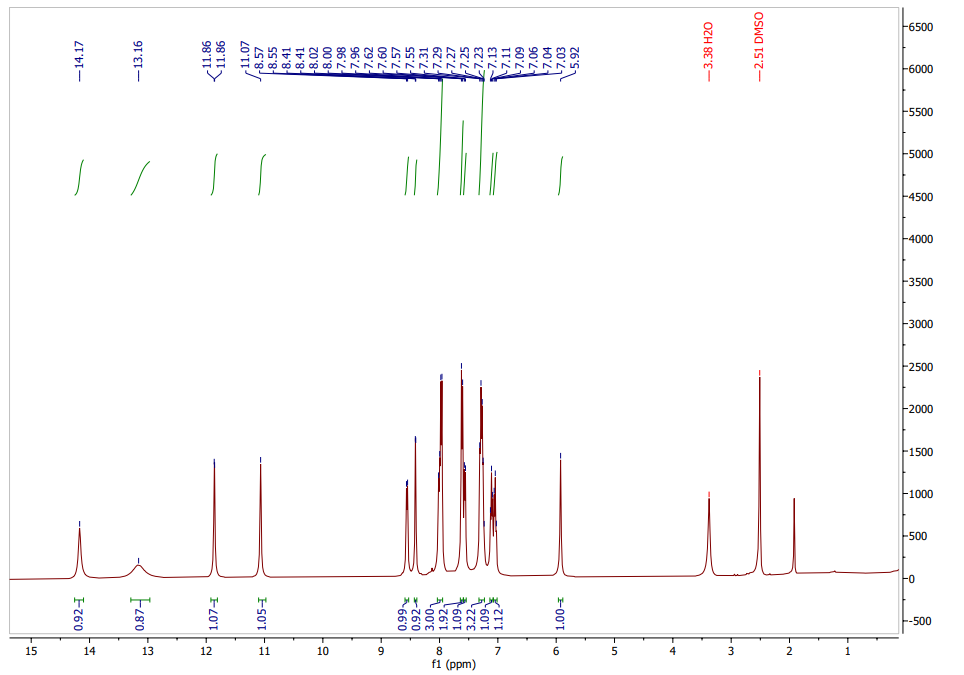
***

# ^1^H-NMR spectrum of 4-(5-cyano-3,6-di(1H-indol-3-yl)-1H-pyrazolo[3,4-b]pyridin-4-yl)benzoic acid (C12).

***
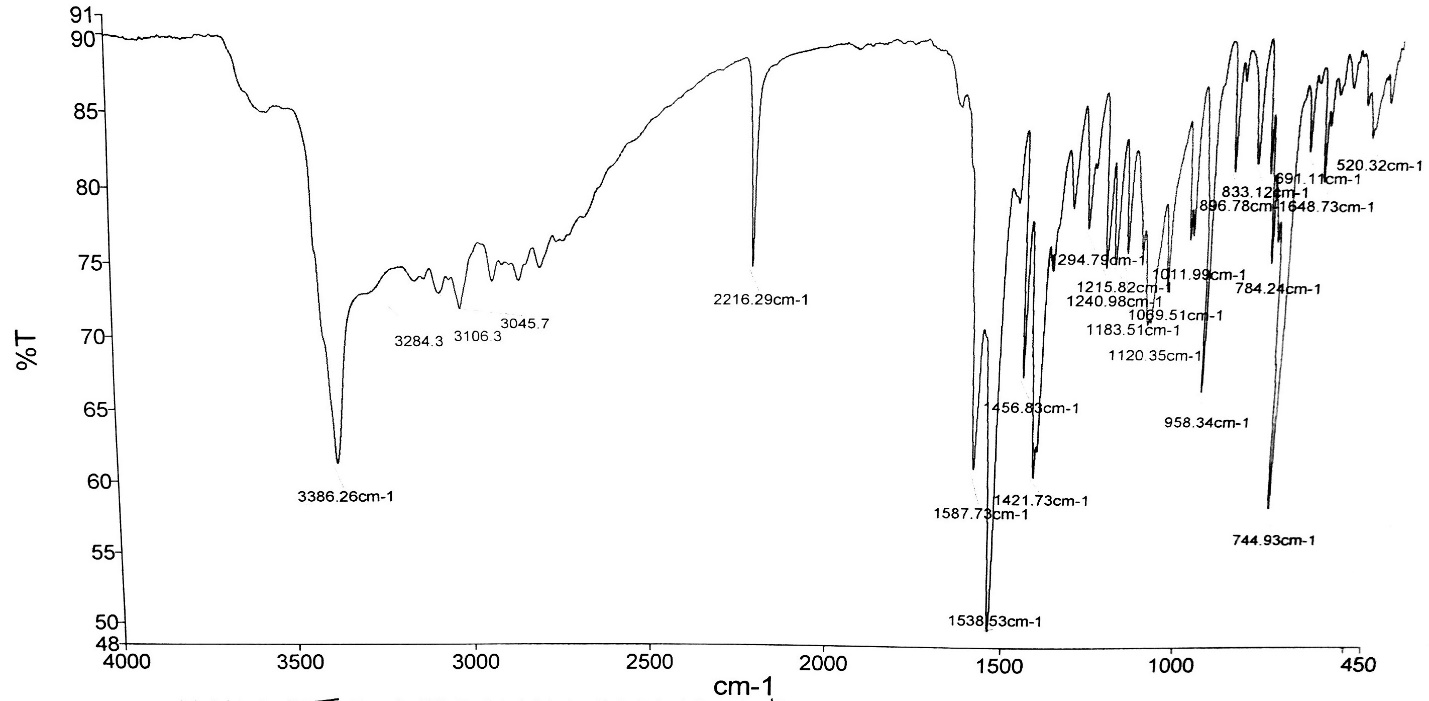
***

# FT-IR spectrum of 3,6-di(1H-indol-3-yl)-4-(pyridin-4-yl)-1H-pyrazolo[3,4-b]pyridine-5-carbonitrile (C13).

***
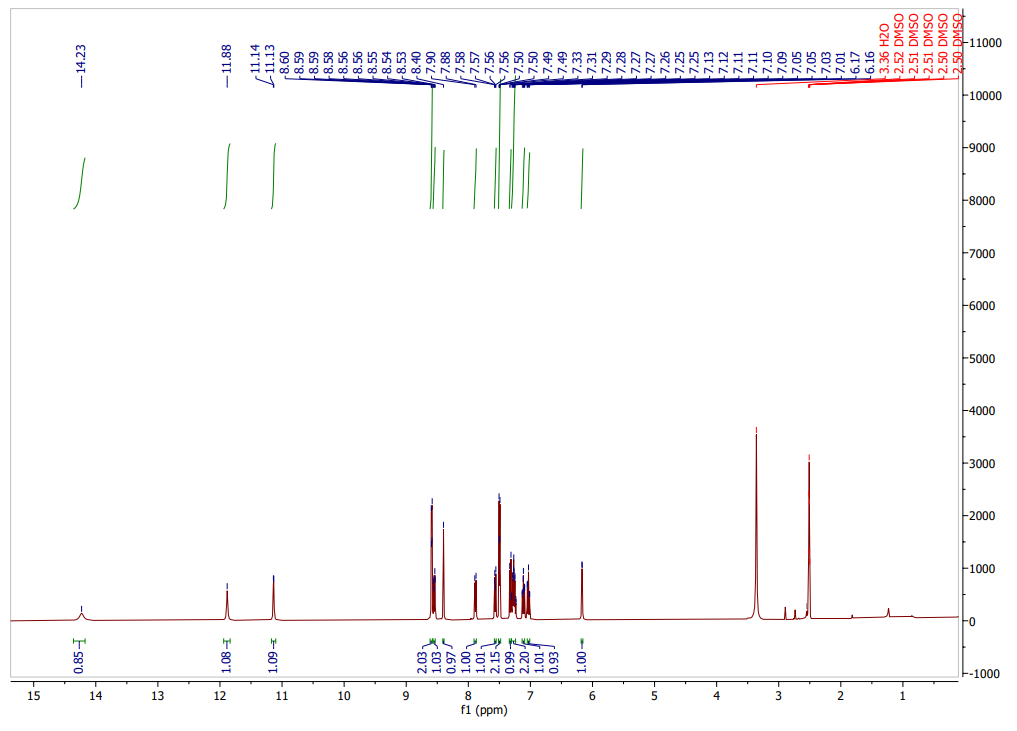
***

# ^1^H-NMR spectrum of 3,6-di(1H-indol-3-yl)-4-(pyridin-4-yl)-1H-pyrazolo[3,4-b]pyridine-5-carbonitrile (C13).

***
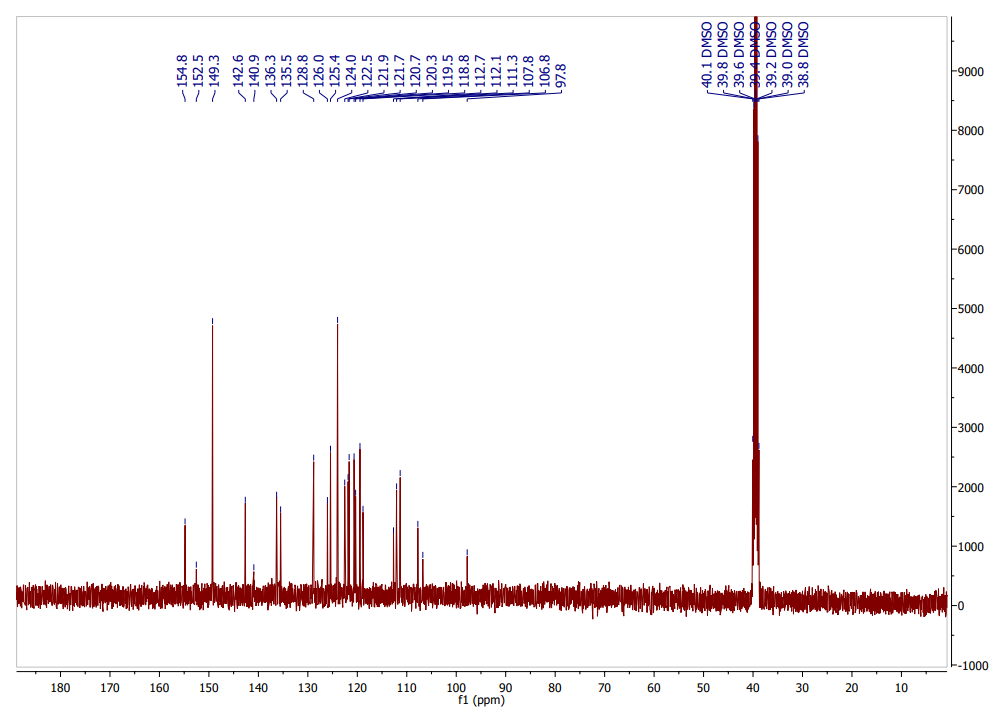
***

# ^13^C-NMR spectrum of 3,6-di(1H-indol-3-yl)-4-(pyridin-4-yl)-1H-pyrazolo[3,4-b]pyridine-5-carbonitrile (C13).

***
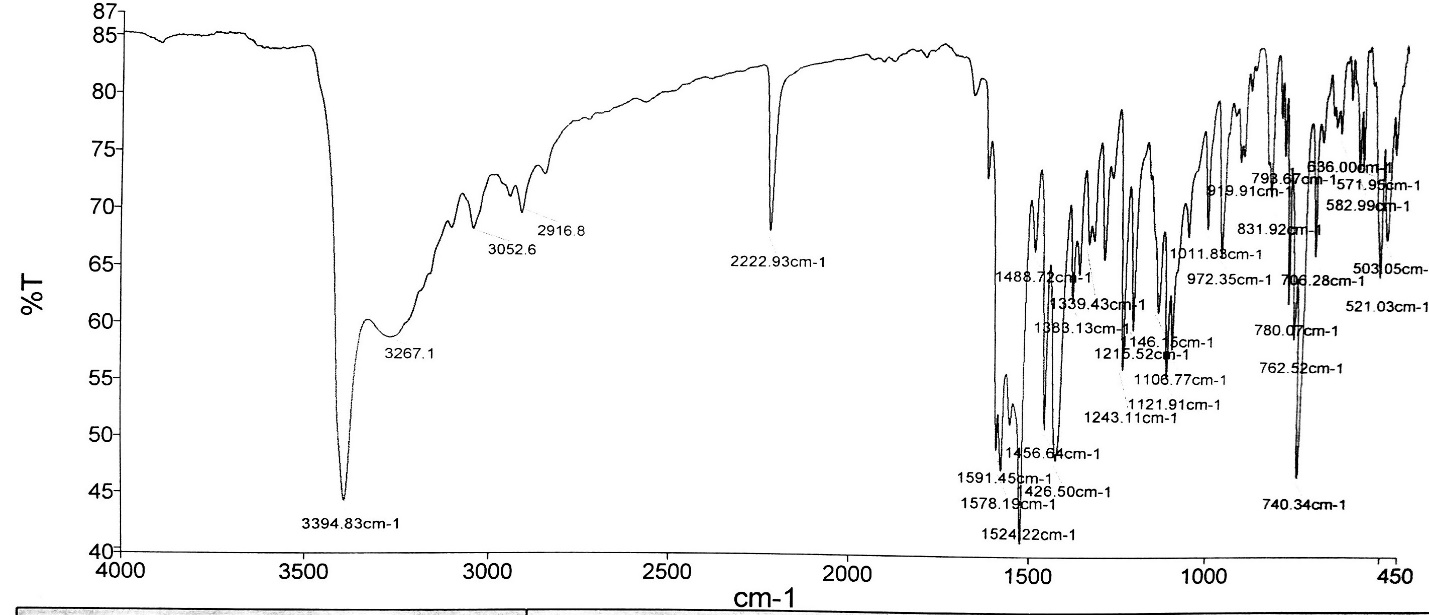
***

# FT-IR spectrum of 3,6-di(1H-indol-3-yl)-4-(m-tolyl)-1H-pyrazolo[3,4-b]pyridine-5-carbonitrile (C14).

***
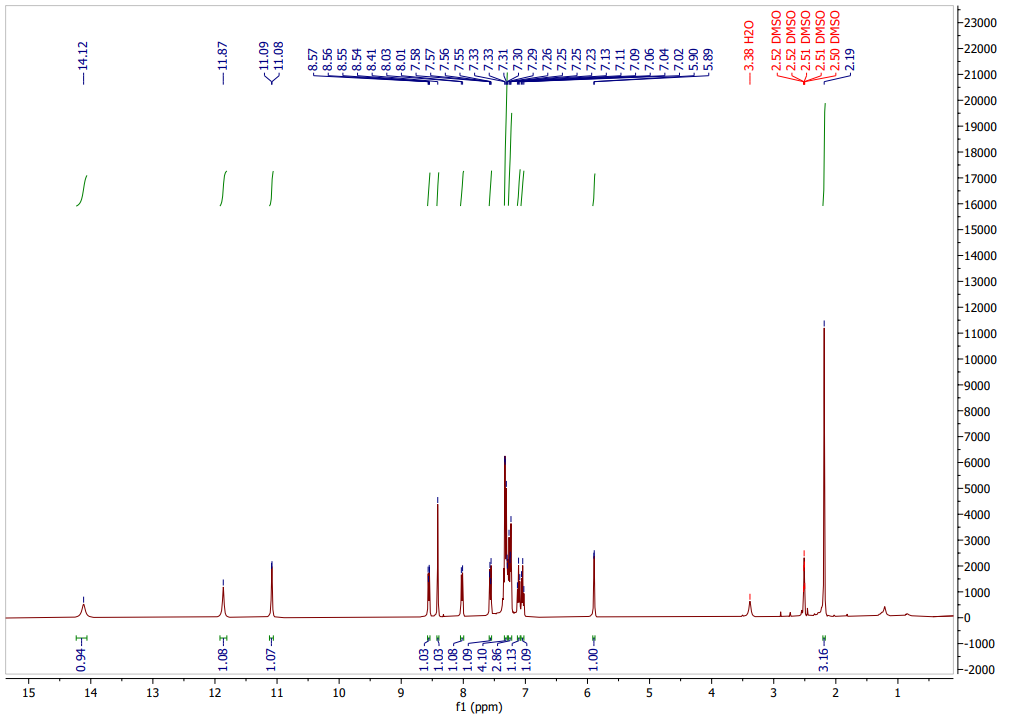
***

# ^1^H-NMR spectrum of 3,6-di(1H-indol-3-yl)-4-(m-tolyl)-1H-pyrazolo[3,4-b]pyridine-5-carbonitrile (C14).


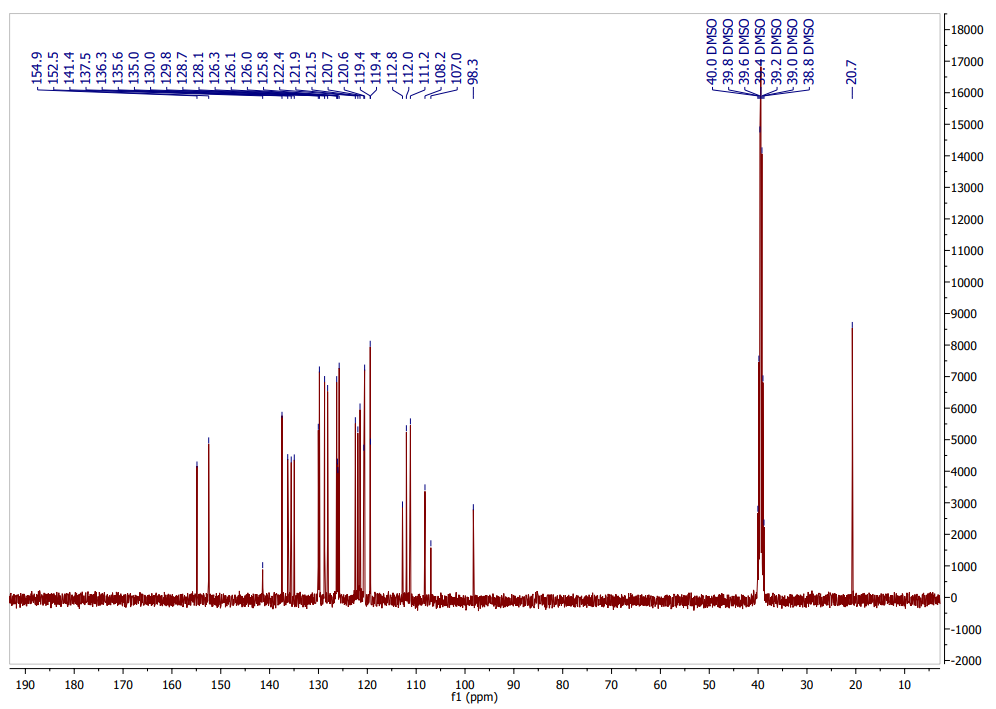


# ^13^C-NMR spectrum of 3,6-di(1H-indol-3-yl)-4-(m-tolyl)-1H-pyrazolo[3,4-b]pyridine-5-carbonitrile (C14).

***
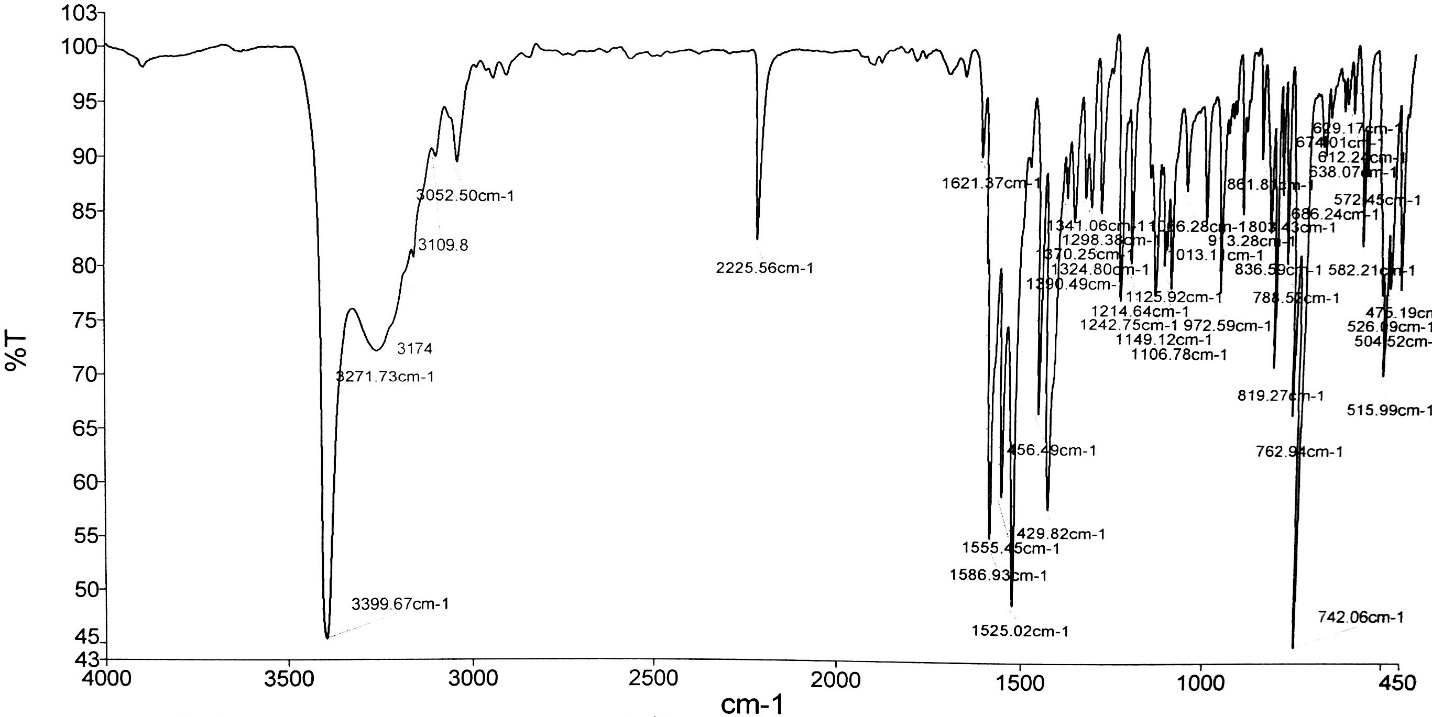
***

# FT-IR spectrum of 3,6-di(1H-indol-3-yl)-4-(naphthalen-2-yl)-1H-pyrazolo[3,4-b]pyridine-5-carbonitrile (C15).

***
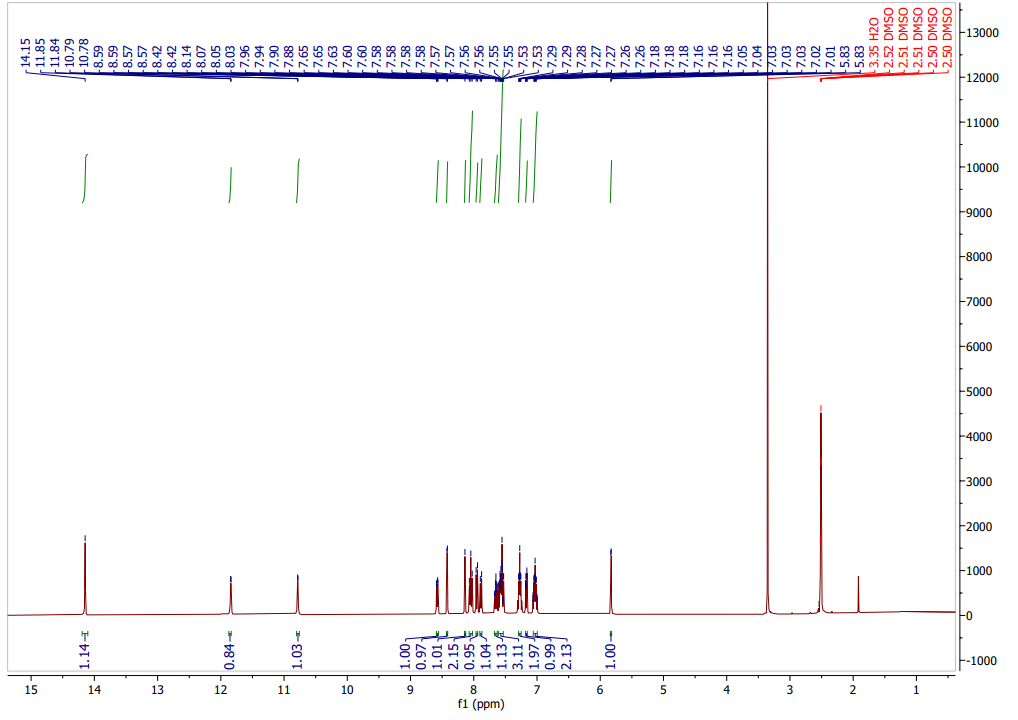
***

# ^1^H-NMR spectrum of 3,6-di(1H-indol-3-yl)-4-(naphthalen-2-yl)-1H-pyrazolo[3,4-b]pyridine-5-carbonitrile (C15).

***
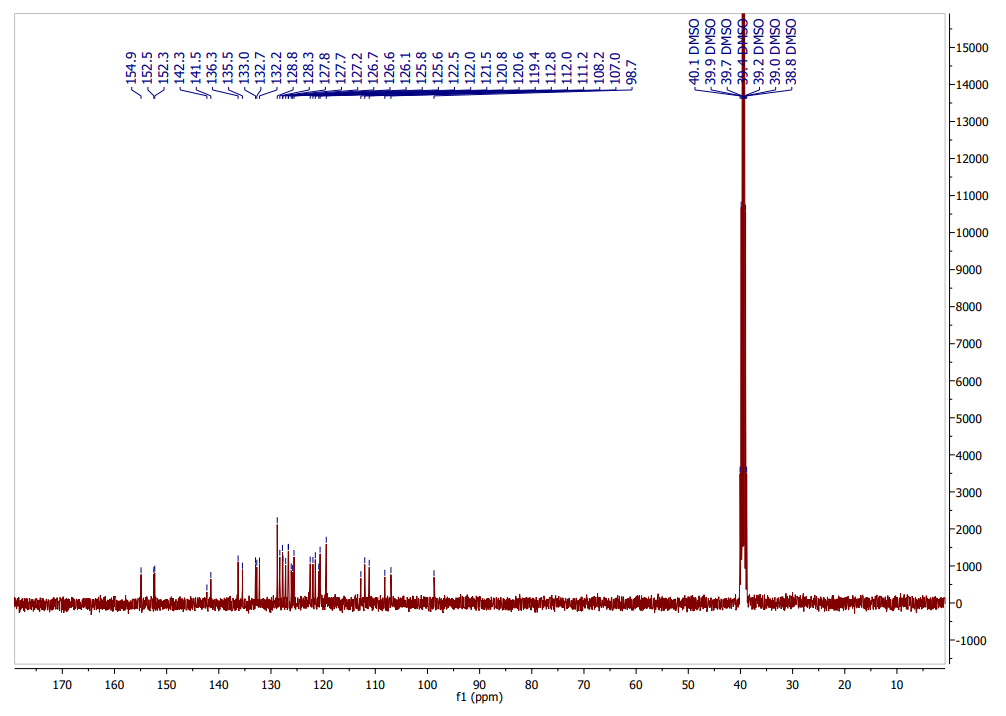
***

# ^13^C-NMR spectrum of 3,6-di(1H-indol-3-yl)-4-(naphthalen-2-yl)-1H-pyrazolo[3,4-b]pyridine-5-carbonitrile (C15).

***
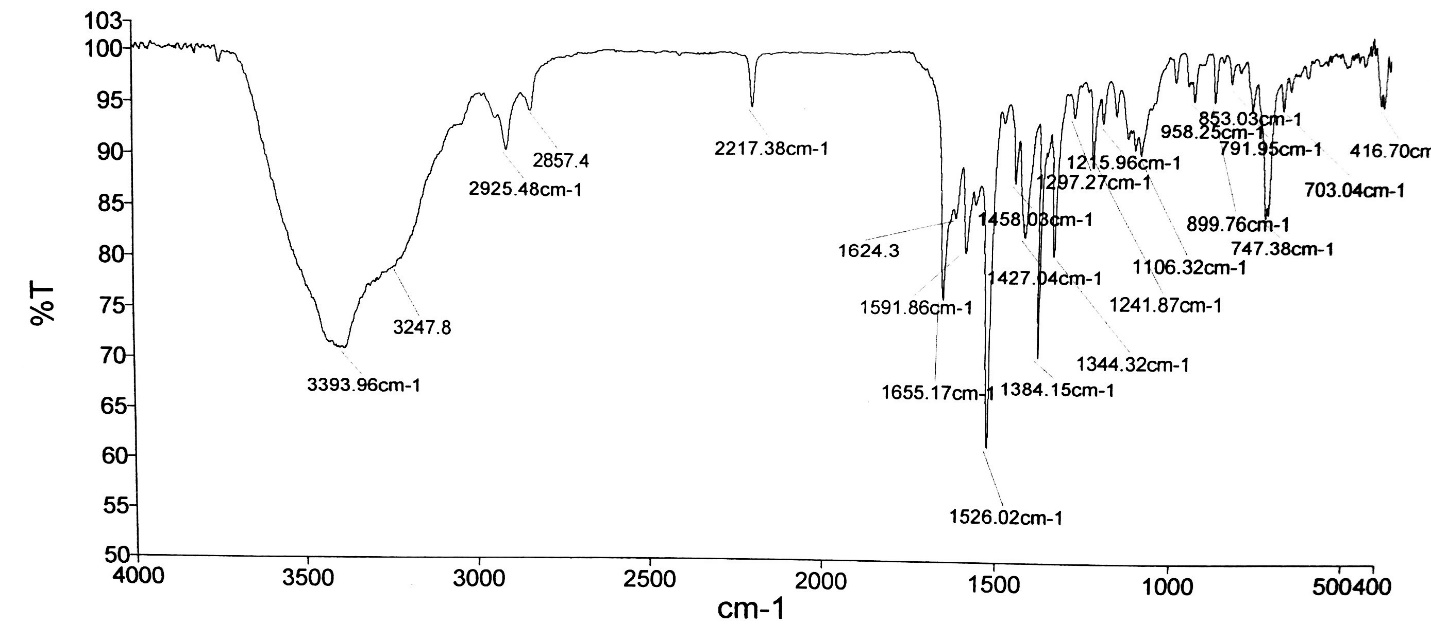
***

# FT-IR spectrum of 3,6-di(1H-indol-3-yl)-4-(naphthalen-1-yl)-1H-pyrazolo[3,4-b]pyridine-5-carbonitrile (C16).

***
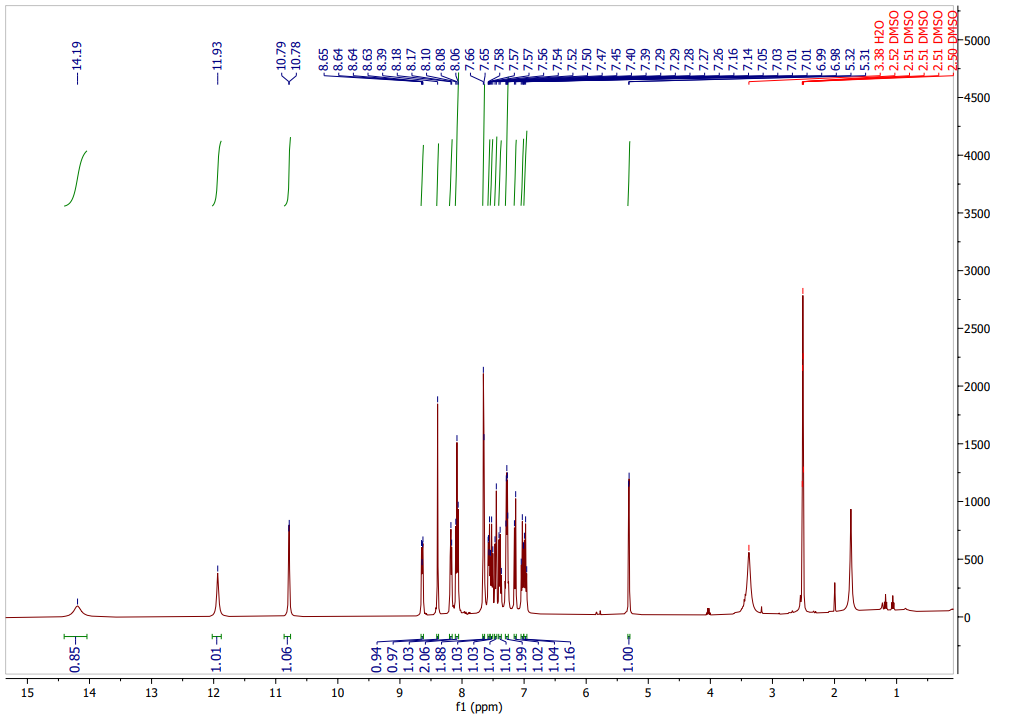
***

# ^1^H-NMR spectrum of 3,6-di(1H-indol-3-yl)-4-(naphthalen-1-yl)-1H-pyrazolo[3,4-b]pyridine-5-carbonitrile (C16).

***
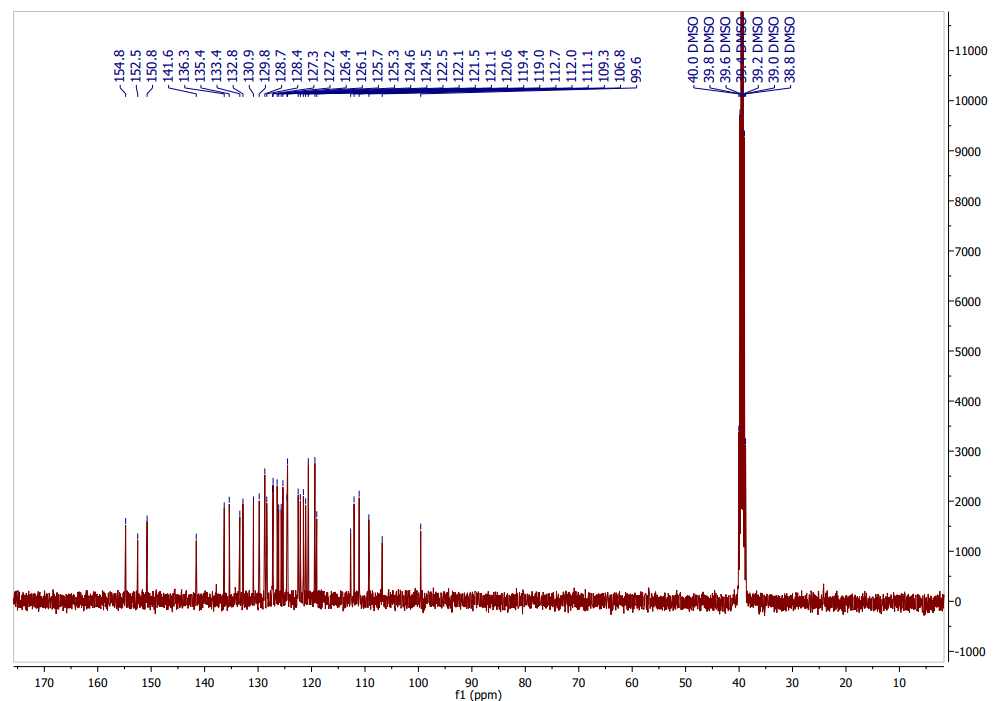
***

# ^13^C-NMR spectrum of 3,6-di(1H-indol-3-yl)-4-(naphthalen-1-yl)-1H-pyrazolo[3,4-b]pyridine-5-carbonitrile (C16).

***
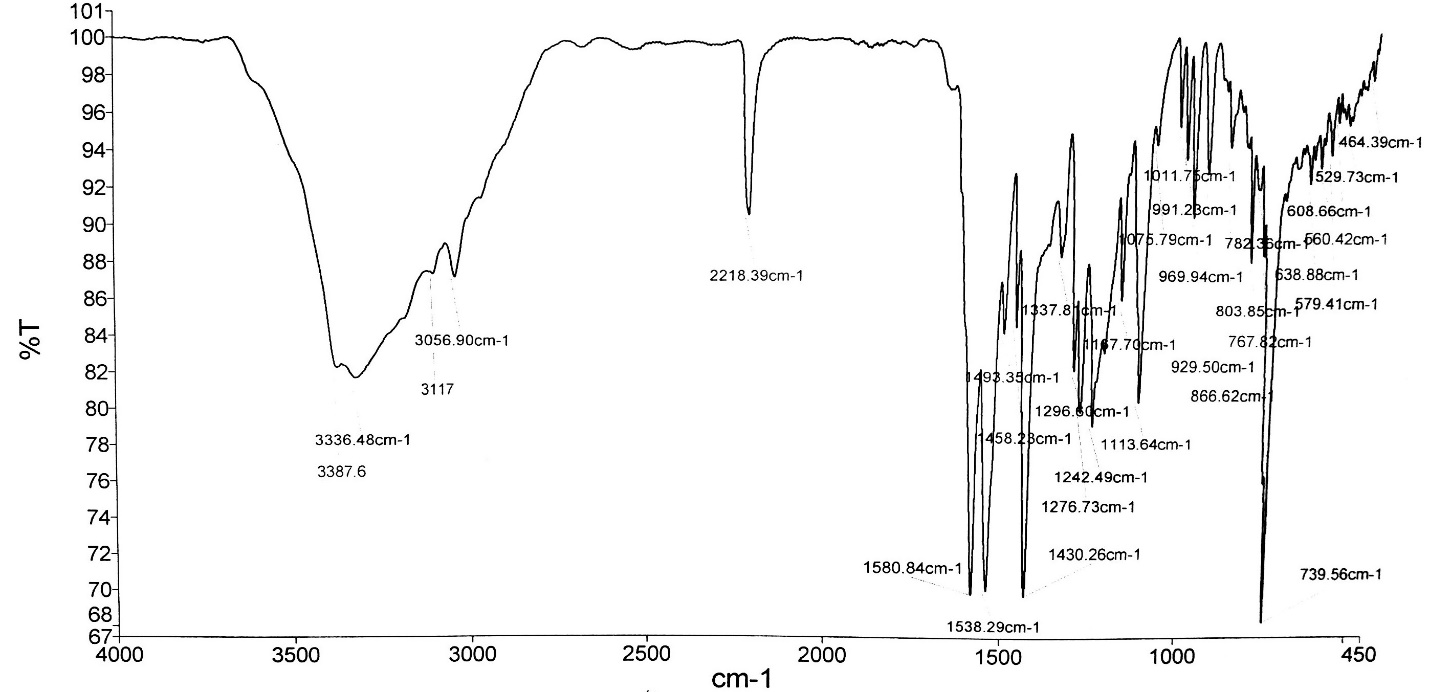
***

# FT-IR spectrum of 4-(3,4-dihydroxyphenyl)-3,6-di(1H-indol-3-yl)-1H-pyrazolo[3,4-b]pyridine-5-carbonitrile (C17).

***
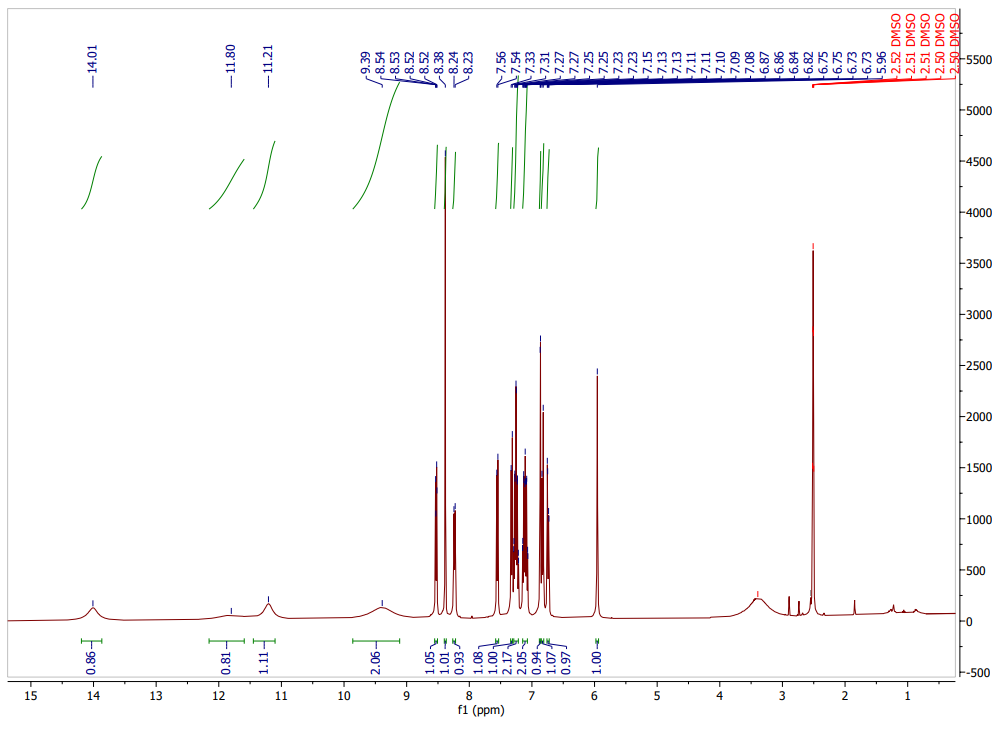
***

# ^1^H-NMR spectrum of 4-(3,4-dihydroxyphenyl)-3,6-di(1H-indol-3-yl)-1H-pyrazolo[3,4-b]pyridine-5-carbonitrile (C17).

***
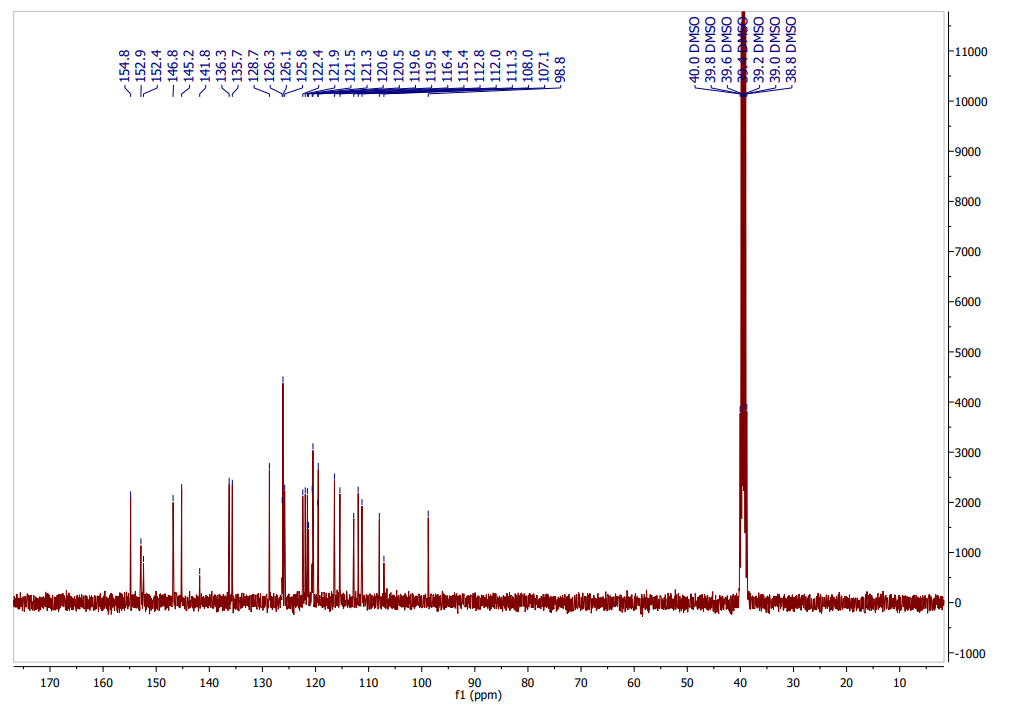
***

# ^13^C-NMR spectrum of 4-(3,4-dihydroxyphenyl)-3,6-di(1H-indol-3-yl)-1H-pyrazolo[3,4-b]pyridine-5-carbonitrile (C17).

***
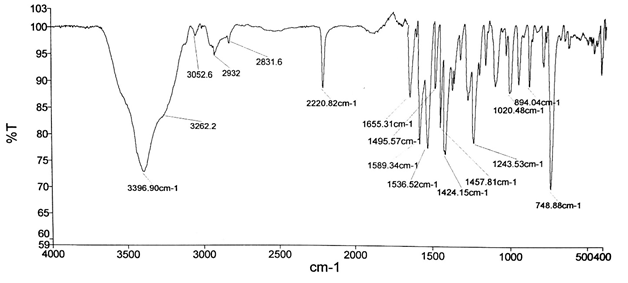
***

# FT-IR spectrum of 3,6-di(1H-indol-3-yl)-4-(2-methoxyphenyl)-1H-pyrazolo[3,4-b]pyridine-5-carbonitrile (C18).

***
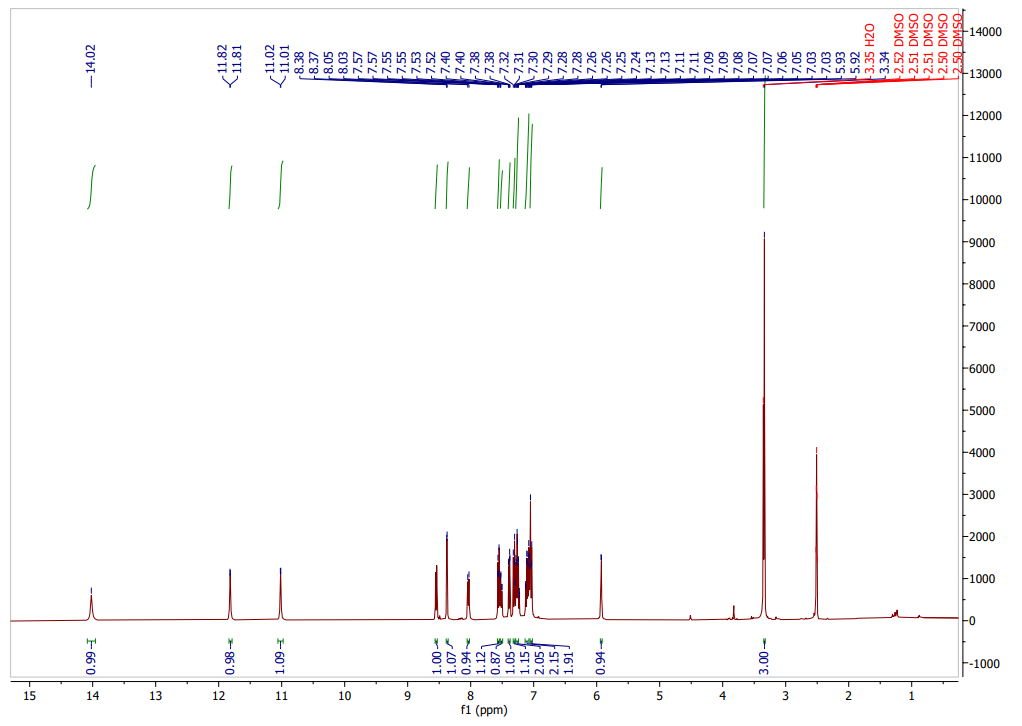
***

# ^1^H-NMR spectrum of 3,6-di(1H-indol-3-yl)-4-(2-methoxyphenyl)-1H-pyrazolo[3,4-b]pyridine-5-carbonitrile (C18).

***
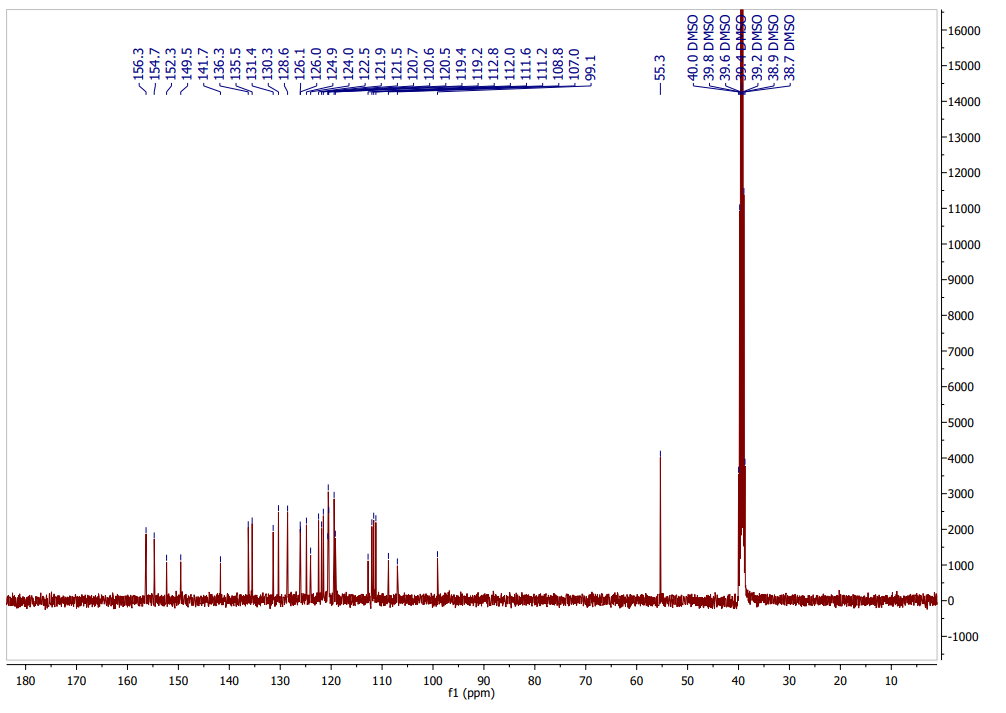
***

# ^13^C-NMR spectrum of 3,6-di(1H-indol-3-yl)-4-(2-methoxyphenyl)-1H-pyrazolo[3,4-b]pyridine-5-carbonitrile (C18).

***
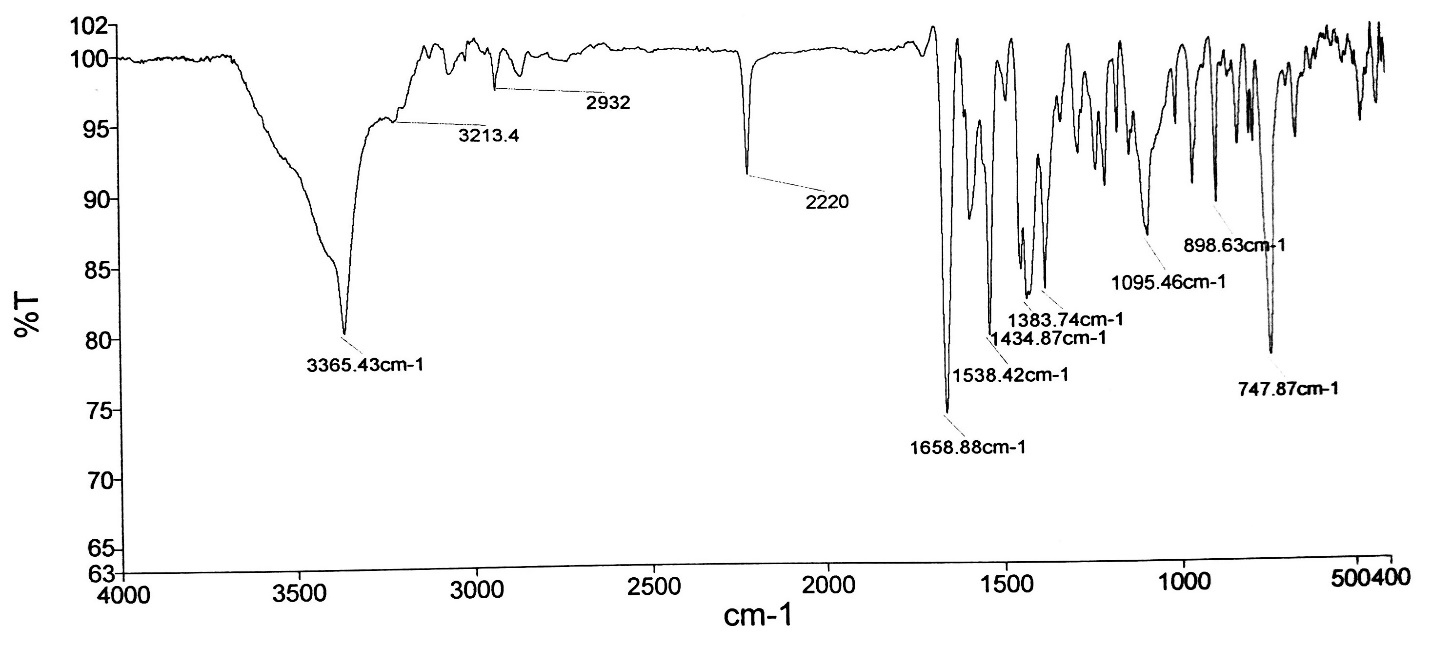
***

# FT-IR spectrum of 4-(2-hydroxyphenyl)-3,6-di(1H-indol-3-yl)-1H-pyrazolo[3,4-b]pyridine-5-carbonitrile (C19).

***
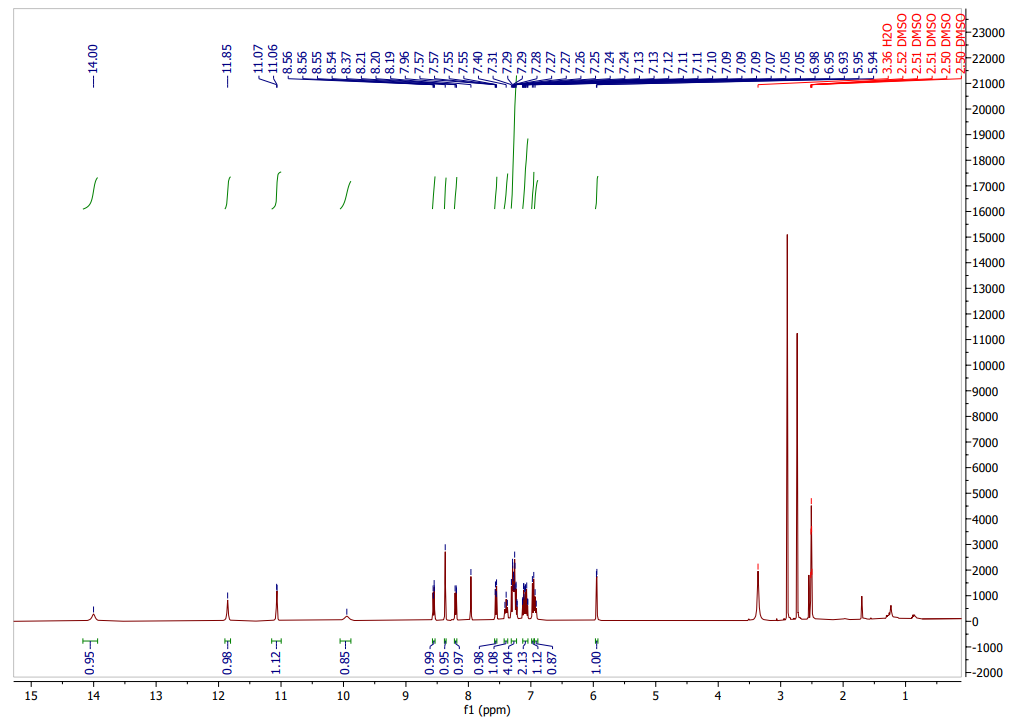
***

# ^1^H-NMR spectrum of 4-(2-hydroxyphenyl)-3,6-di(1H-indol-3-yl)-1H-pyrazolo[3,4-b]pyridine-5-carbonitrile (C19

***
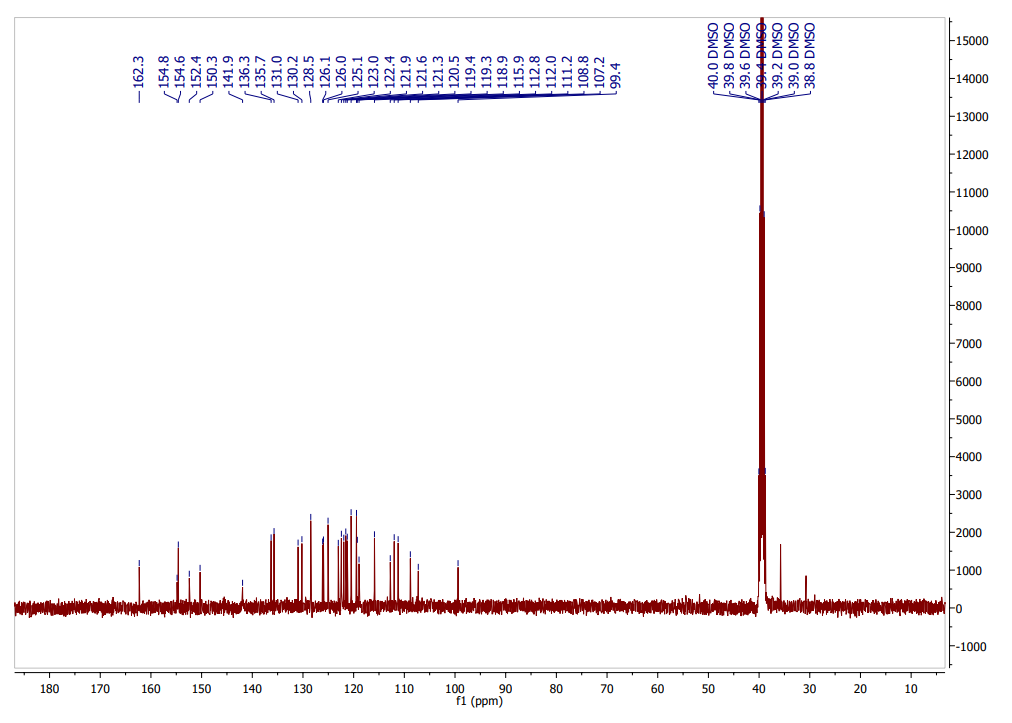
***

# ^13^C-NMR spectrum of 4-(2-hydroxyphenyl)-3,6-di(1H-indol-3-yl)-1H-pyrazolo[3,4-b]pyridine-5-carbonitrile (C19).

***
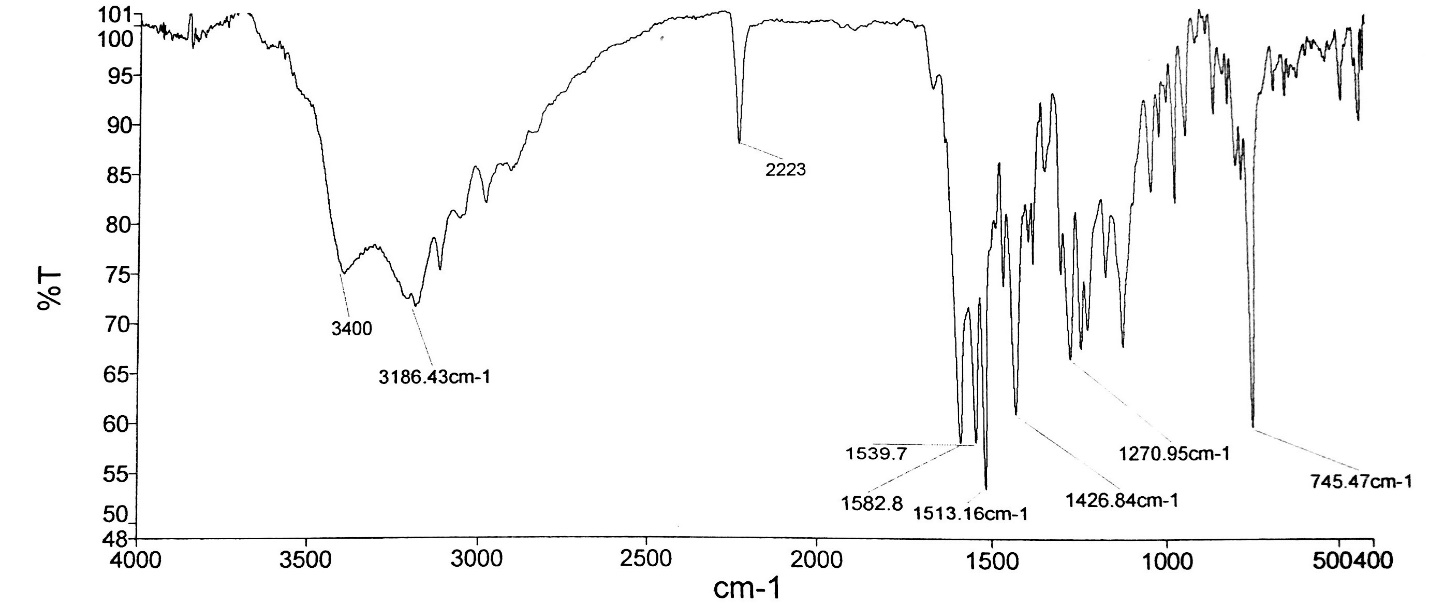
***

# FT-IR spectrum of 4-(3-ethoxy-4-hydroxyphenyl)-3,6-di(1H-indol-3-yl)-1H-pyrazolo[3,4-b]pyridine-5-carbonitrile (C20).

*
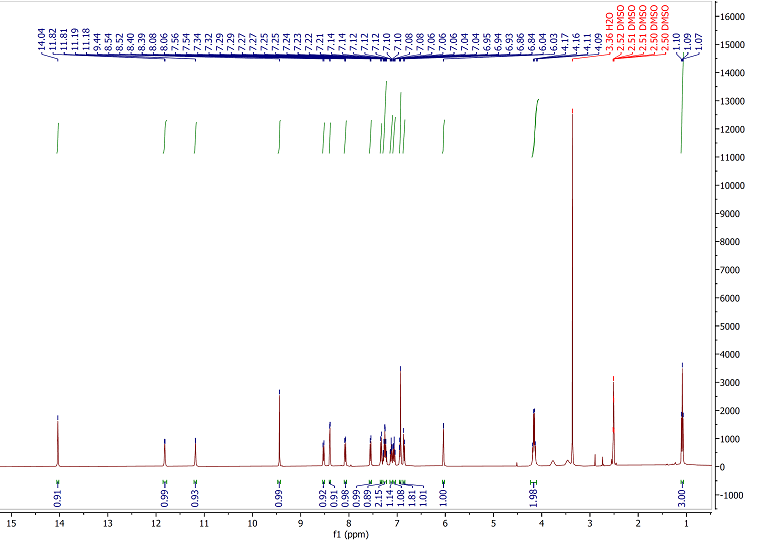
*

# ^1^H-NMR spectrum of 4-(3-ethoxy-4-hydroxyphenyl)-3,6-di(1H-indol-3-yl)-1H-pyrazolo[3,4-b]pyridine-5-carbonitrile (C20).

***
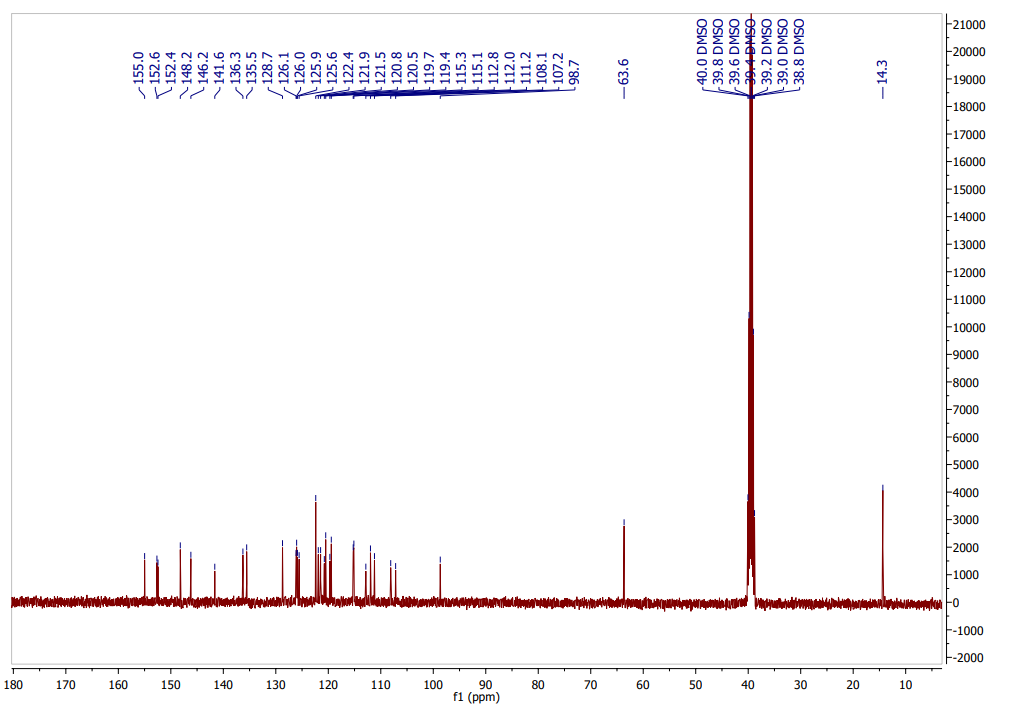
***

# ^13^C-NMR spectrum of 4-(3-ethoxy-4-hydroxyphenyl)-3,6-di(1H-indol-3-yl)-1H-pyrazolo[3,4-b]pyridine-5-carbonitrile (C20).

***
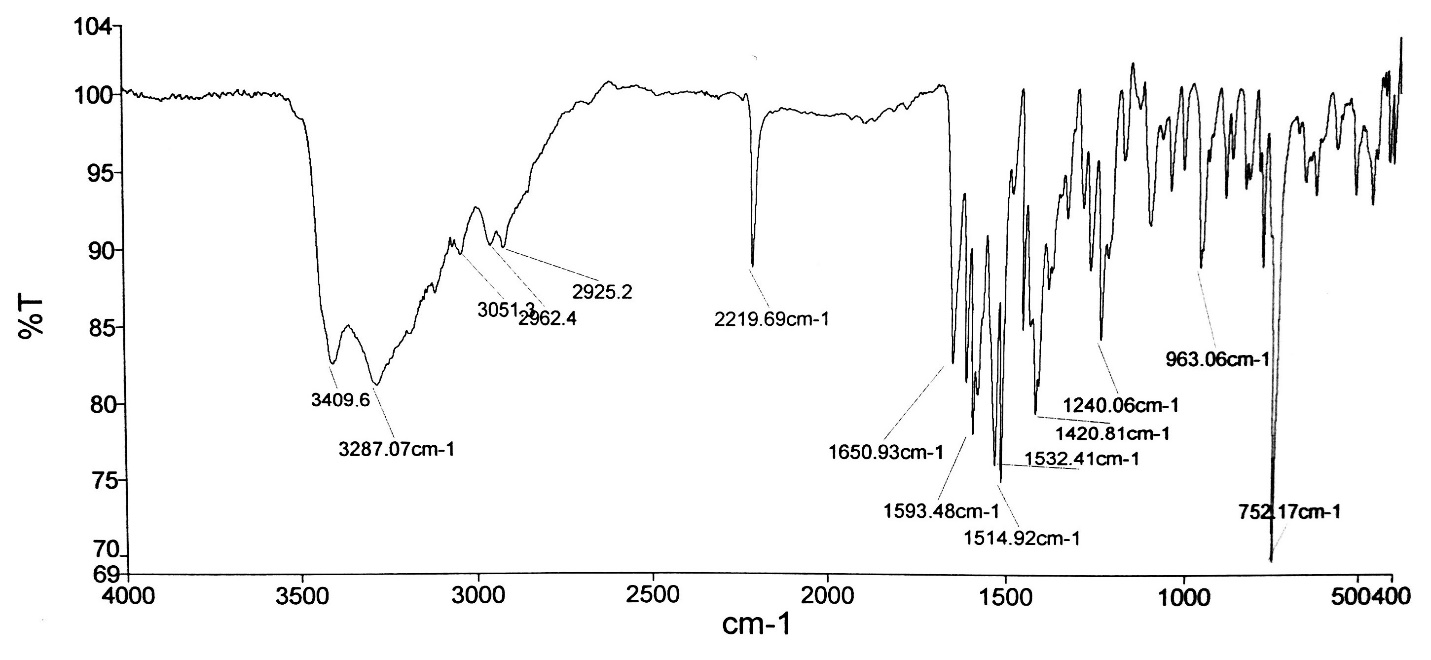
***

# FT-IR spectrum of 4,4',4''-(((1,3,5-triazine-2,4,6-triyl)tris(oxy))tris(benzene-4,1-diyl))tris(3,6-di(1H-indol-3-yl)-4,7-dihydro-1H-pyrazolo[3,4-b]pyridine-5-carbonitrile) (C21).

***
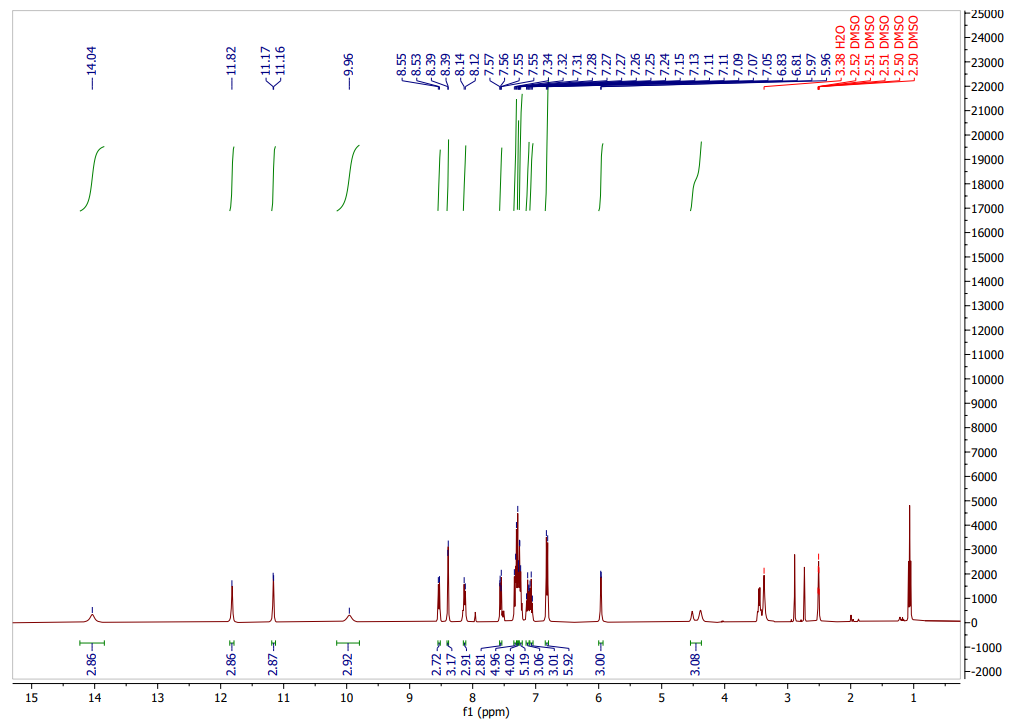
***

# ^1^H-NMR spectrum of 4,4',4''-(((1,3,5-triazine-2,4,6-triyl)tris(oxy))tris(benzene-4,1-diyl))tris(3,6-di(1H-indol-3-yl)-4,7-dihydro-1H-pyrazolo[3,4-b]pyridine-5-carbonitrile) (C21).

***
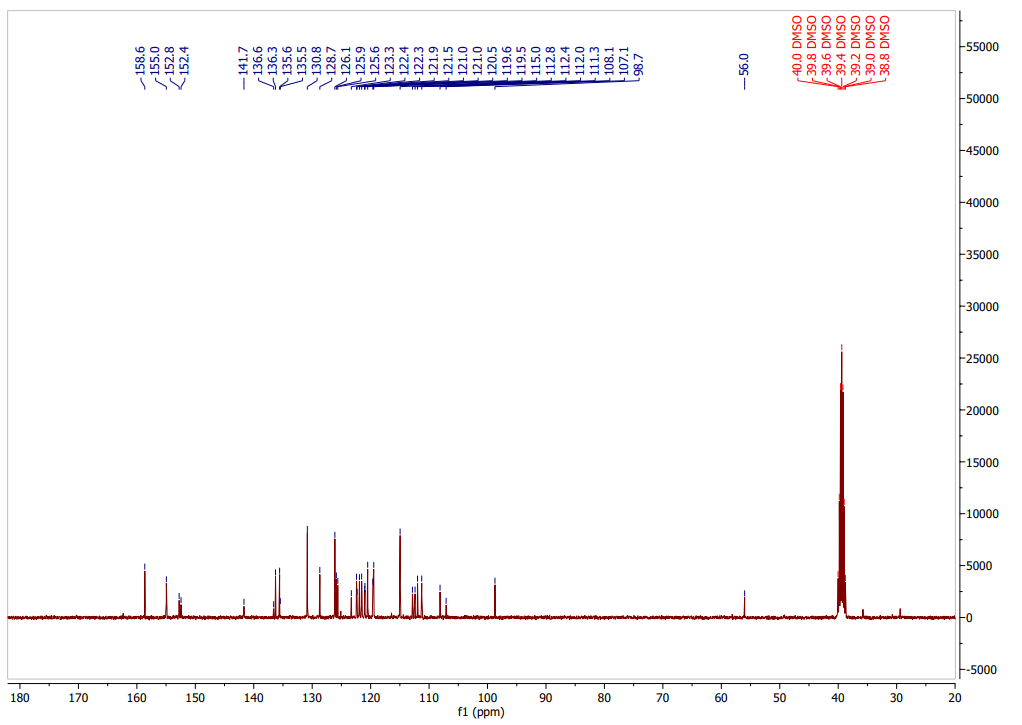
***

# ^13^C-NMR spectrum of 4,4',4''-(((1,3,5-triazine-2,4,6-triyl)tris(oxy))tris(benzene-4,1-diyl))tris(3,6-di(1H-indol-3-yl)-4,7-dihydro-1H-pyrazolo[3,4-b]pyridine-5-carbonitrile) (C21).

***
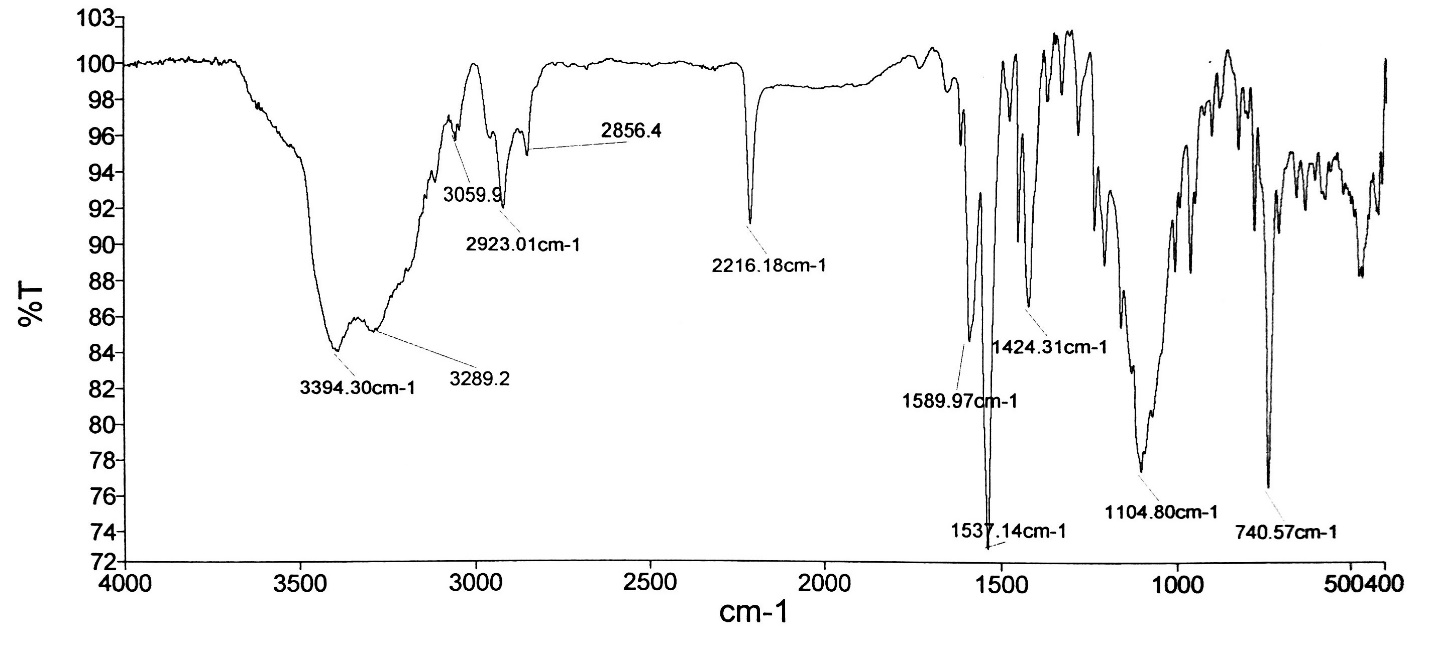
***

# FT-IR spectrum of 4,4'-(1,3-phenylene)bis(3,6-di(1H-indol-3-yl)-1H-pyrazolo[3,4-b]pyridine-5-carbonitrile) (C22).

***
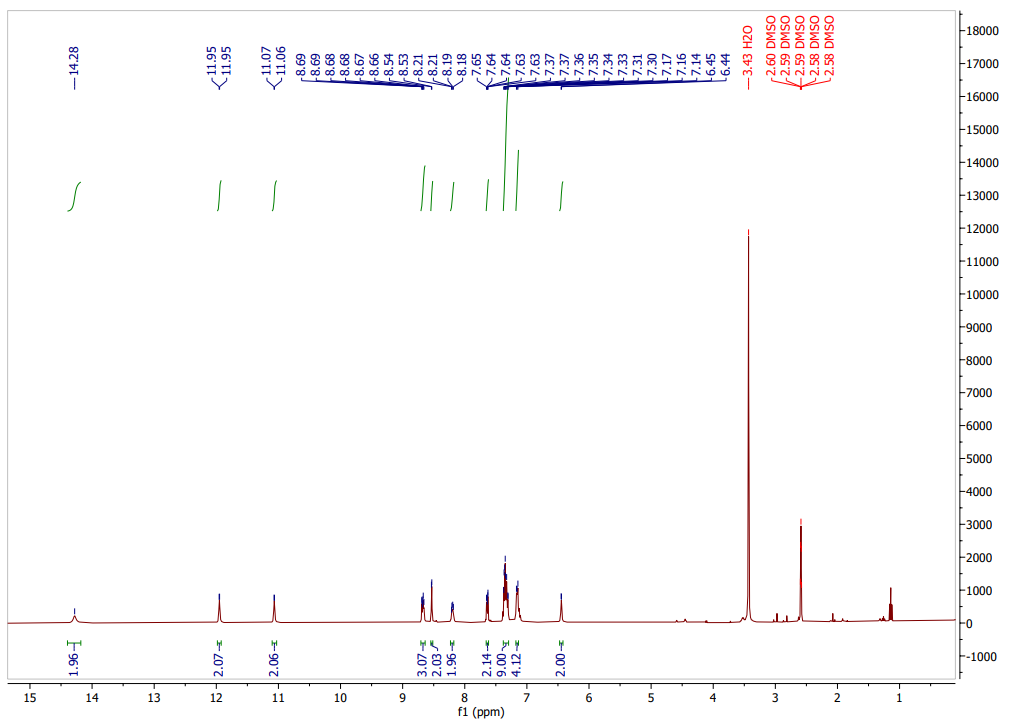
***

# ^1^H-NMR spectrum of 4,4'-(1,3-phenylene)bis(3,6-di(1H-indol-3-yl)-1H-pyrazolo[3,4-b]pyridine-5-carbonitrile) (C22).

***
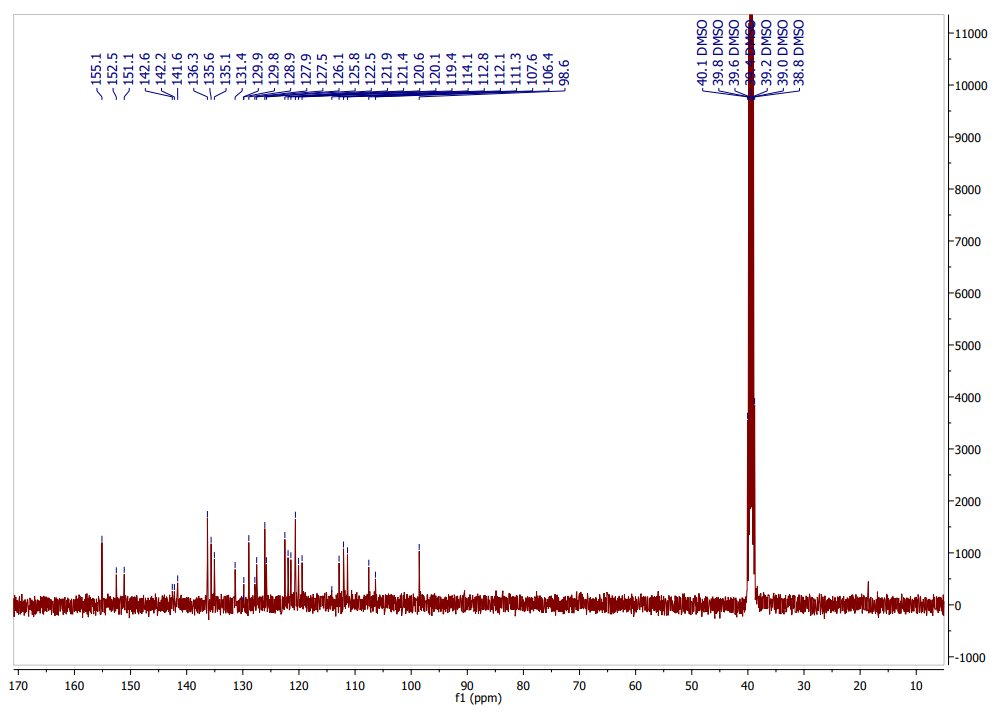
***

# ^13^C-NMR spectrum of 4,4'-(1,3-phenylene)bis(3,6-di(1H-indol-3-yl)-1H-pyrazolo[3,4-b]pyridine-5-carbonitrile) (C22).
